# Large meta-analysis of genome-wide association studies identifies five loci for lean body mass

**DOI:** 10.1038/s41467-017-00031-7

**Published:** 2017-07-19

**Authors:** M. Carola Zillikens, Serkalem Demissie, Yi-Hsiang Hsu, Laura M. Yerges-Armstrong, Wen-Chi Chou, Lisette Stolk, Gregory Livshits, Linda Broer, Toby Johnson, Daniel L. Koller, Zoltán Kutalik, Jian’an Luan, Ida Malkin, Janina S. Ried, Albert V. Smith, Gudmar Thorleifsson, Liesbeth Vandenput, Jing Hua Zhao, Weihua Zhang, Ali Aghdassi, Kristina Åkesson, Najaf Amin, Leslie J. Baier, Inês Barroso, David A. Bennett, Lars Bertram, Rainer Biffar, Murielle Bochud, Michael Boehnke, Ingrid B. Borecki, Aron S. Buchman, Liisa Byberg, Harry Campbell, Natalia Campos Obanda, Jane A. Cauley, Peggy M. Cawthon, Henna Cederberg, Zhao Chen, Nam H. Cho, Hyung Jin Choi, Melina Claussnitzer, Francis Collins, Steven R. Cummings, Philip L. De Jager, Ilja Demuth, Rosalie A. M. Dhonukshe-Rutten, Luda Diatchenko, Gudny Eiriksdottir, Anke W. Enneman, Mike Erdos, Johan G. Eriksson, Joel Eriksson, Karol Estrada, Daniel S. Evans, Mary F. Feitosa, Mao Fu, Melissa Garcia, Christian Gieger, Thomas Girke, Nicole L. Glazer, Harald Grallert, Jagvir Grewal, Bok-Ghee Han, Robert L. Hanson, Caroline Hayward, Albert Hofman, Eric P. Hoffman, Georg Homuth, Wen-Chi Hsueh, Monica J. Hubal, Alan Hubbard, Kim M. Huffman, Lise B. Husted, Thomas Illig, Erik Ingelsson, Till Ittermann, John-Olov Jansson, Joanne M. Jordan, Antti Jula, Magnus Karlsson, Kay-Tee Khaw, Tuomas O. Kilpeläinen, Norman Klopp, Jacqueline S. L. Kloth, Heikki A. Koistinen, William E. Kraus, Stephen Kritchevsky, Teemu Kuulasmaa, Johanna Kuusisto, Markku Laakso, Jari Lahti, Thomas Lang, Bente L. Langdahl, Lenore J. Launer, Jong-Young Lee, Markus M. Lerch, Joshua R. Lewis, Lars Lind, Cecilia Lindgren, Yongmei Liu, Tian Liu, Youfang Liu, Östen Ljunggren, Mattias Lorentzon, Robert N. Luben, William Maixner, Fiona E. McGuigan, Carolina Medina-Gomez, Thomas Meitinger, Håkan Melhus, Dan Mellström, Simon Melov, Karl Michaëlsson, Braxton D. Mitchell, Andrew P. Morris, Leif Mosekilde, Anne Newman, Carrie M. Nielson, Jeffrey R. O’Connell, Ben A. Oostra, Eric S. Orwoll, Aarno Palotie, Stephen C. J. Parker, Munro Peacock, Markus Perola, Annette Peters, Ozren Polasek, Richard L. Prince, Katri Räikkönen, Stuart H. Ralston, Samuli Ripatti, John A. Robbins, Jerome I. Rotter, Igor Rudan, Veikko Salomaa, Suzanne Satterfield, Eric E. Schadt, Sabine Schipf, Laura Scott, Joban Sehmi, Jian Shen, Chan Soo Shin, Gunnar Sigurdsson, Shad Smith, Nicole Soranzo, Alena Stančáková, Elisabeth Steinhagen-Thiessen, Elizabeth A. Streeten, Unnur Styrkarsdottir, Karin M. A. Swart, Sian-Tsung Tan, Mark A. Tarnopolsky, Patricia Thompson, Cynthia A. Thomson, Unnur Thorsteinsdottir, Emmi Tikkanen, Gregory J. Tranah, Jaakko Tuomilehto, Natasja M. van Schoor, Arjun Verma, Peter Vollenweider, Henry Völzke, Jean Wactawski-Wende, Mark Walker, Michael N. Weedon, Ryan Welch, H.-Erich Wichmann, Elisabeth Widen, Frances M. K. Williams, James F. Wilson, Nicole C. Wright, Weijia Xie, Lei Yu, Yanhua Zhou, John C. Chambers, Angela Döring, Cornelia M. van Duijn, Michael J. Econs, Vilmundur Gudnason, Jaspal S. Kooner, Bruce M. Psaty, Timothy D. Spector, Kari Stefansson, Fernando Rivadeneira, André G. Uitterlinden, Nicholas J. Wareham, Vicky Ossowski, Dawn Waterworth, Ruth J. F. Loos, David Karasik, Tamara B. Harris, Claes Ohlsson, Douglas P. Kiel

**Affiliations:** 1 Department of Internal Medicine, Erasmus MC, Rotterdam, 3000 The Netherlands; 2 Netherlands Genomics Initiative (NGI)-sponsored Netherlands Consortium for Healthy Aging (NCHA), Leiden, 2593 The Netherlands; 3 Department of Biostatistics, Boston University School of Public Health, Boston, MA 02118 USA; 4 Hebrew SeniorLife, Institute for Aging Research, Roslindale, MA 02131 USA; 5 Harvard Medical School, Boston, MA 02115 USA; 6 Molecular and Integrative Physiological Sciences Program, Harvard School of Public Health, Boston, MA 02115 USA; 7 Program in Personalized and Genomic Medicine, and Department of Medicine, Division of Endocrinology, Diabetes and Nutrition, University of Maryland School of Medicine, Baltimore, MD 21201 USA; 8 Broad Institute, Cambridge, MA 02142 USA; 9 Sackler Faculty of Medicine, Department of Anatomy and Anthropology, Tel Aviv University, Tel Aviv, 6997801 Israel; 10 Department of Twin Research and Genetic Epidemiology, King’s College London, St Thomas’ Campus, London, WC2R 2LS UK; 11 Department of Epidemiology, Erasmus MC, Rotterdam, 3000 The Netherlands; 12 Department of Medical Genetics, University of Lausanne, Lausanne, 1011 Switzerland; 13 Swiss Institute of Bioinformatics, Lausanne, 1015 Switzerland; 14 Centre Hospitalier Universitaire (CHUV), University Institute for Social and Preventive Medicine, Lausanne, 1010 Switzerland; 15 Department of Medical and Molecular Genetics, Indiana University School of Medicine, Indianapolis, IN 46202 USA; 16 MRC Epidemiology Unit, University of Cambridge School of Clinical Medicine, Cambridge Biomedical Campus, Cambridge, CB2 OQQ UK; 17 Institute of Epidemiology II, Helmholtz Zentrum München – German Research Center for Environmental Health, Neuherberg, 85764 Germany; 18 Icelandic Heart Association, Kopavogur, 201 Iceland; 19 Faculty of Medicine, University of Iceland, Reykjavik, 101 Iceland; 20 deCODE Genetics, Reykjavik, 101 Iceland; 21 Department of Internal Medicine, Institute of Medicine, Sahlgrenska Academy, University of Gothenburg, Gothenburg, SE-405 30 Sweden; 22 Department Epidemiology and Biostatistics, School of Public Health, Imperial College, London, SW7 2AZ UK; 23 Cardiology Department, Ealing Hospital NHS Trust, Middlesex, UB1 3HW UK; 24 Department of Medicine A, University of Greifswald, Greifswald, 17489 Germany; 25 Department of Clinical Sciences, Lund University, Malmö, 22362 Sweden; 26 Department of Orthopedics, Skåne University Hospital, Malmö, S-205 02 Sweden; 27 Phoenix Epidemiology and Clinical Research Branch, National Institute of Diabetes and Digestive and Kidney Diseases, NIH, Phoenix, AZ 85014 USA; 28 Wellcome Trust Sanger Institute, Wellcome Trust Genome Campus, Hinxton, CB10 1SA UK; 29 NIHR Cambridge Biomedical Research Centre, Institute of Metabolic Science, Addenbrooke’s Hospital, Cambridge, CB2 OQQ UK; 30 Institute of Metabolic Science, Addenbrooke’s Hospital, University of Cambridge Metabolic Research Laboratories, Cambridge, CB2 OQQ UK; 31 Rush Alzheimer’s Disease Center, Rush University Medical Center, Chicago, IL 60612 USA; 32 Lübeck Interdisciplinary Platform for Genome Analytics, Institutes of Neurogenetics and Experimental & Integrative Genomics, University of Lübeck, Lübeck, 23562 Germany; 33 School of Public Health, Faculty of Medicine, Imperial College London, London, W6 8RP UK; 34 Centre of Oral Health, Department of Prosthetic Dentistry, Gerodontology and Biomaterials, University of Greifswald, Greifswald, 17489 Germany; 35 Department of Biostatistics and Center for Statistical Genetics, University of Michigan, Ann Arbor, MI 48109 USA; 36 Division of Statistical Genomics, Department of Genetics, Washington University School of Medicine, St Louis, MO 63110 USA; 37 Division of Biostatistics, Washington University School of Medicine, St Louis, MO 63110 USA; 38 Department of Surgical Sciences, Uppsala University, Uppsala, 75185 Sweden; 39 Usher Institute of Population Health Sciences and Informatics, University of Edinburgh, Edinburgh, Scotland EH8 9AG UK; 40 Department of Epidemiology Graduate School of Public Health, University of Pittsburgh, Pittsburgh, PA 15261 USA; 41 California Pacific Medical Center Research Institute, San Francisco, CA 94107 USA; 42 Department of Medicine, University of Eastern Finland and Kuopio University Hospital, Kuopio, 70210 Finland; 43 Mel and Enid Zuckerman College of Public Health, University of Arizona, Tucson, AZ 85714 USA; 44 Department of Preventive Medicine, Ajou University School of Medicine, Youngtong-Gu, Suwon 16499 Korea; 45 Department of Internal Medicine, Seoul National University College of Medicine, Seoul, 03080 Korea; 46 Department of Internal Medicine, Chungbuk National University Hospital, Cheongju Si, Korea; 47 Computer Science and Artificial Intelligence Laboratory, MIT, Cambridge, MA 02139 USA; 48 Institute of Human Genetics, MRI, Technische Universität München, Munich, 81675 Germany; 49 Beth Israel Deaconess Medical Center, Boston, MA 02215 USA; 50 Medical Genomics and Metabolic Genetics Branch, National Human Genome Research Institute, Bethesda, MD 20892 USA; 51 Program in Translational NeuroPsychiatric Genomics, Department of Neurology, Brigham and Women’s Hospital, Boston, MA 02115 USA; 52 Program in Medical and Population Genetics, Broad Institute, Cambridge, MA 02142 USA; 53 Lipid Clinic at the Interdisciplinary Metabolism Center, Charité – Universitätsmedizin Berlin, corporate member of Freie Universität Berlin, Humboldt-Universität zu Berlin, and Berlin Institute of Health, Berlin, 13353 Germany; 54 Institute of Medical and Human Genetics, Charité – Universitätsmedizin Berlin, Berlin, 13353 Germany; 55 Department of Human Nutrition, Wageningen University, PO Box 17, Wageningen, 6700 AA The Netherlands; 56 Alan Edwards Centre for Research on Pain, McGill University, Montreal, H3A 0G1 Canada; 57 Regional Center for Neurosensory Disorders, School of Dentistry, University of North Carolina at Chapel Hill, Chapel Hill, NC 27599 USA; 58 Department of General Practice and Primary Health Care, University of Helsinki, Helsinki, 00014 Finland; 59 Unit of General Practice, Helsinki University Central Hospital, Helsinki, 00014 Finland; 60 Folkhalsan Research Centre, Helsinki, 00250 Finland; 61 Vasa Central Hospital, Vasa, 65130 Finland; 62 National Institute for Health and Welfare, Helsinki, 00271 Finland; 63 Laboratory of Epidemiology and Population Sciences, Intramural Research Program, National Institute for Aging, Bethesda, MD 20892 USA; 64 Research Unit of Molecular Epidemiology, Helmholtz Zentrum München - German Research Center for Environmental Health, Neuherberg, 85764 Germany; 65 Institute of Genetic Epidemiology, Helmholtz Zentrum München - German Research Center for Environmental Health, Neuherberg, 85764 Germany; 66 Institute for Integrative Genome Biology, University of California, Riverside, CA 92521 USA; 67 Department of Botany and Plant Sciences, University of California, Riverside, CA 92521 USA; 68 Departments of Medicine and Epidemiology, Boston University School of Medicine and Public Health, Boston, MA 02118 USA; 69 German Center for Diabetes Research (DZD), Neuherberg, Germany; 70 CCG Type 2 Diabetes, Helmholtz Zentrum München, Neuherberg, 85764 Germany; 71 CCG Nutrigenomics and Type 2 Diabetes. Helmholtz Zentrum München, Neuherberg, 85764 Germany; 72 National Heart and Lung Institute, Imperial College London, London, SW3 6LY UK; 73 Center for Genome Science, National Institute of Health, Osong Health Technology Administration Complex, Chungcheongbuk-do, 28159 Korea; 74 MRC Human Genetics Unit, IGMM, University of Edinburgh, Edinburgh, Scotland EH4 2XU UK; 75 Department of Pharmaceutical Sciences, SUNY Binghamton, Binghamton, NY 13902 USA; 76 Interfaculty Institute for Genetics and Functional Genomics, University of Greifswald, Greifswald, 17487 Germany; 77 Department of Exercise and Nutrition Sciences, George Washington University, Washington, DC 20052 USA; 78 Research Center for Genetic Medicine, Children’s National Medical Center, Washington, DC 20052 USA; 79 Division of Biostatistics, School of Public Health, University of California, Berkeley, CA 94720 USA; 80 Division of Rheumatology, Department of Medicine, Duke Molecular Physiology Institute, Duke University School of Medicine, Durham, NC 27710 USA; 81 Endocrinology and Internal Medicine, Aarhus University Hospital, Aarhus, DK 8000 Denmark; 82 Department of Human Genetics, Hannover Medical School, Hannover, 30625 Germany; 83 Hannover Unified Biobank, Hannover Medical School, Hannover, 30625 Germany; 84 Department of Medical Sciences, Uppsala University, Uppsala, 75185 Sweden; 85 Division of Cardiovascular Medicine, Department of Medicine, Stanford University School of Medicine, Stanford, CA 94305 USA; 86 Institute for Community Medicine, University of Greifswald, Greifswald, 17489 Germany; 87 Department of Physiology, Institute of Neuroscience and Physiology, Sahlgrenska Academy, University of Gothenburg, Gothenburg, SE 405 30 Sweden; 88 Thurston Arthritis Research Center, University of North Carolina at Chapel Hill, Chapel Hill, NC 27517 USA; 89 Department of Clinical Sciences and Orthopaedics, Lund University, Skåne University Hospital SUS, Malmö, 22362 Sweden; 90 Department of Public Health and Primary Care, University of Cambridge, Cambridge, CB1 8RN UK; 91 The Novo Nordisk Foundation Center for Basic Metabolic Research, Section of Metabolic Genetics, University of Copenhagen, Copenhagen, 2100 Denmark; 92 Department of Environmental Medicine and Public Health, Icahn School of Medicine at Mount Sinai, New York, NY 10029 USA; 93 Department of Medicine, University of Helsinki and Helsinki University Central Hospital, Helsinki, 00029 Finland; 94 Endocrinology, Abdominal Center, University of Helsinki and Helsinki University Central Hospital, Helsinki, 00029 Finland; 95 Department of Health, National Institute for Health and Welfare, Helsinki, 00271 Finland; 96 Minerva Foundation Institute for Medical Research, Helsinki, 00290 Finland; 97 Division of Cardiology, Department of Medicine, Duke Molecular Physiology Institute, Duke University School of Medicine, Durham, NC 27710 USA; 98 Sticht Center on Aging, Wake Forest School of Medicine, Winston-Salem, NC 27157 USA; 99 Institute of Behavioural Sciences, University of Helsinki, Helsinki, FI00014 Finland; 100 University of California San Francisco, San Francisco, CA 94143 USA; 101 School of Medicine and Pharmacology, University of Western Australia, Perth, 6009 Australia; 102 Centre for Kidney Research, School of Public Health, University of Sydney, Sydney, 2006 Australia; 103 Wellcome Trust Centre for Human Genetics, Oxford University, Oxford, OX3 7BN UK; 104 Department of Epidemiology and Prevention, Wake Forest School of Medicine, Winston-Salem, NC 27517 USA; 105 Max Planck Institute for Molecular Genetics, Berlin, 14195 Germany; 106 Max Planck Institute for Human Development, Berlin, 14195 Germany; 107 Institute of Human Genetics, Helmholtz Zentrum München - German Research Center for Environmental Health, Neuherberg, 85764 Germany; 108 Buck Institute for Research on Aging, Novato, CA 94945 USA; 109 Leonard Davis School of Gerontology, University of Southern California, LA, CA 90089 USA; 110 Geriatrics Research and Education Clinical Center, Baltimore Veterans Administration Medical Center, Baltimore, MD 21201 USA; 111 Institute of Translational Medicine, University of Liverpool, Liverpool, L69 3BX UK; 112 Center for Aging and Population Health, University of Pittsburgh, Pittsburgh, PA 15261 USA; 113 Oregon Health & Science University, Portland, OR 97239 USA; 114 Department of Clinical Genetics, Erasmus MC, Rotterdam, 300 CA The Netherlands; 115 Centre for Medical Systems Biology and Netherlands Consortium on Healthy Aging, Leiden, RC2300 The Netherlands; 116 Institute for Molecular Medicine Finland (FIMM), University of Helsinki, Helsinki, 00251 Finland; 117 Department of Medical Genetics, University of Helsinki and University Central Hospital, Helsinki, FI00014 Finland; 118 Human Genetics and Computational Medicine and Bioinformatics, University of Michigan, Ann Arbor, MI 48109 USA; 119 Department of Medicine, Indiana University School of Medicine, Indianapolis, IN 46202 USA; 120 Diabetes and Obesity Research Program, University of Helsinki, Helsinki, FI00014 Finland; 121 Estonian Genome Center, University of Tartu, Tartu, Estonia; 122 Faculty of Medicine, Department of Public Health, University of Split, Split, 21000 Croatia; 123 Department of Endocrinology and Diabetes, Sir Charles Gardiner Hospital, Perth, 6009 Australia; 124 Molecular Medicine Centre, MRC Institute of Genetics and Molecular Medicine, Western General Hospital, Edinburgh, Scotland EH4 2XU UK; 125 Hjelt Institute, University of Helsinki, Helsinki, Finland; 126 Wellcome Trust Sanger Institute, Wellcome Trust Genome Campus, Hinxton, CB10 1SA UK; 127 Department of Medicine, University of California at Davis, Sacramento, CA 95817 USA; 128 Institute for Translational Genomic and Population Sciences, Los Angeles Biomedical Research Institute and Department of Pediatrics, Harbor UCLA Medical Center, Torrance, CA 90502 USA; 129 Department of Preventive Medicine, University of Tennessee Health Science Center, Memphis, TN 38163 USA; 130 Department of Genetics and Genomic Science, Institute of Genomics and Multiscale Biology, Icahn School of Medicine at Mount Sinai, New York, NY 10029 USA; 131 Department of Endocrinology and Metabolism, Landspitali, The National University Hospital of Iceland, Reykjavik, 101 Iceland; 132 Center for Translational Pain Medicine, Department of Anesthiology, Duke University Medical Center, Durham, NC 27110 USA; 133 Geriatric Research and Education Clinical Center (GRECC) - Veterans Administration Medical Center, Baltimore, MD 21201 USA; 134 Department of Epidemiology and Biostatistics, and the EMGO Institute, VU University Medical Center, Amsterdam, BT1081 The Netherlands; 135 Department of Medicine, McMaster University Medical Center, Hamilton, ON Canada L8N 3Z5; 136 Department of Pathology, Stony Brook School of Medicine, Stony Brook, NY 11794 USA; 137 Department of Neuroscience and Preventive Medicine, Danube-University Krems, Krems, 3500 Austria; 138 Diabetes Research Group, King Abdulaziz University, Jeddah, 12589 Saudi Arabia; 139 Dasman Diabetes Institute, Dasman, 15462 Kuwait; 140 Department of Medicine and Internal Medicine, Centre Hospitalier Universitaire Vaudois (CHUV), Lausanne, CH-1011 Switzerland; 141 Department of Epidemiology and Environmental Health, University at Buffalo, State University of New York, Buffalo, NY 14214 USA; 142 Institute of Cellular Medicine, Newcastle University, Newcastle upon Tyne, NE2 4HH UK; 143 Genetics of Complex Traits, University of Exeter Medical School, Exeter, EX1 2LU UK; 144 Institute of Medical Informatics, Biometry and Epidemiology, Chair of Epidemiology, Ludwig-Maximilians-Universität, Munich, 81377 Germany; 145 Institute of Medical Statistics and Epidemiology, Technical University, Munich, 81675 Germany; 146 Department of Epidemiology, University of Alabama at Birmingham, Birmingham, AL 35294 USA; 147 NIHR Cardiovascular Biomedical Research Unit, Royal Brompton and Harefield NHS Foundation Trust and Imperial College, London, SW3 6NP UK; 148 Imperial College Healthcare NHS Trust, London, W2 1NY UK; 149 Institute of Epidemiology I, Helmholtz Zentrum München - German Research Center for Environmental Health, Neuherberg, 85764 Germany; 150 Department of Medicine and Department of Medical and Molecular Genetics, Indiana University School of Medicine, Indianapolis, IN 46202 USA; 151 Departments of Medicine, Epidemiology, and Health Services, Cardiovascular Health Research Unit, University of Washington, Seattle, WA 98101 USA; 152 Kaiser Permanente Washington Health Research Institute, Washington, Seattle, WA 98101 USA; 153 Medical Genetics, GlaxoSmithKline, Philadelphia, PA 19112 USA; 154 The Charles Bronfman Institute for Personalized Medicine, Icahn School of Medicine at Mount Sinai, New York, NY 10029 USA; 155 Institute of Child Health and Development, Icahn School of Medicine at Mount Sinai, New York, NY 10029 USA; 156 The Genetics of Obesity and Related Traits Program, Icahn School of Medicine at Mount Sinai, New York, NY 10029 USA; 157 Department of Preventive Medicine, Icahn School of Medicine at Mount Sinai, New York, NY 10029 USA; 158 Faculty of Medicine in the Galilee, Bar-Ilan University, Safed, 1311502 Israel

## Abstract

Lean body mass, consisting mostly of skeletal muscle, is important for healthy aging. We performed a genome-wide association study for whole body (20 cohorts of European ancestry with *n* = 38,292) and appendicular (arms and legs) lean body mass (*n* = 28,330) measured using dual energy X-ray absorptiometry or bioelectrical impedance analysis, adjusted for sex, age, height, and fat mass. Twenty-one single-nucleotide polymorphisms were significantly associated with lean body mass either genome wide (*p* < 5 × 10^−8^) or suggestively genome wide (*p* < 2.3 × 10^−6^). Replication in 63,475 (47,227 of European ancestry) individuals from 33 cohorts for whole body lean body mass and in 45,090 (42,360 of European ancestry) subjects from 25 cohorts for appendicular lean body mass was successful for five single-nucleotide polymorphisms in/near *HSD17B11, VCAN, ADAMTSL3*, *IRS1*, and *FTO* for total lean body mass and for three single-nucleotide polymorphisms in/near *VCAN*, *ADAMTSL3*, and *IRS1* for appendicular lean body mass. Our findings provide new insight into the genetics of lean body mass.

## Abstract (web summary)

Lean body mass is a highly heritable trait and is associated with various health conditions. Here, Kiel and colleagues perform a meta-analysis of genome-wide association studies for whole body lean body mass and find five novel genetic loci to be significantly associated.

## Introduction

Lean body mass consists primarily of skeletal muscle, is an important contributor to physical strength, mobility, stamina, and balance^1–3^, and has been a very recent focus of an effort to define “sarcopenia” (loss of muscle tissue) for clinical care and drug development^4^. The determinants of adult skeletal muscle mass have not been well characterized. It is known, for example, that exercise produces increases in muscle mass, and there is some evidence that protein intake is directly associated with lean mass^5^. Heavier people have increased muscle mass^6^, which may be due to the loading effect of increased fat mass or may reflect a common genetic background between muscle and adipose tissue^7^. With aging, there is a progressive loss of skeletal muscle mass, and a concurrent increase in fatty infiltration and fibrosis of muscle. This loss of muscle mass may reach a critical point at which time functional impairment and even disability occurs^8^. In fact, the annual healthcare costs of sarcopenia in the United States are estimated to be in excess of 18 billion dollars^9^.

As estimated from family and twin studies, lean mass is a highly heritable phenotype with heritability estimates of 0.52–0.60^10, 11^. While there have been previous studies related to the genetic background of BMI and fat mass, few studies have searched for genes associated with lean mass^12–15, 16, 17^. To date, no single-nucleotide polymorphisms (SNPs) have been found to be associated at a genome-wide significance level with lean mass (*p*-values < 5 × 10^−8^)^18, 19^. A copy number variation located in the *GREM1* gene was reported to be associated with lean body mass in a genome-wide association study (GWAS) of 1627 Chinese^20^. Guo et al.^19^ identified in 1627 Chinese and replicated in 2286 European ancestry individuals, a locus near *CNTF* and *GLYAT* genes at 11q12.1 in a bivariate GWAS for bone-size phenotypes and appendicular lean mass. Most recently in a study of Japanese women, the *PRDM16* gene was suggested to be associated with lean mass^21^.

With the advent of relatively simple, inexpensive methods of measuring the fat and lean compartments of the body using dual energy X-ray absorptiometry (DXA) and bioelectrical impedance analysis (BIA), multiple cohort studies have accumulated phenotypic information on body composition that permits large-scale GWAS to be performed. While whole body lean mass incorporates all of the non-fat soft tissue including the internal organs, appendicular lean mass, estimated by DXA and BIA, may be a better reflection of skeletal muscle mass^22–24^. To identify genetic loci associated with whole body and appendicular lean mass, we performed a large-scale GWAS meta-analysis in over 100,000 participants from 53 studies yielding sufficient power to identify common variants with small to moderate effect sizes.

## Results

### GWAS meta-analyses for discovery and replication

Descriptions and characteristics of the study populations in the discovery stage and the replication stage are shown in Supplementary Tables 4 and 5 and Supplementary Note 2.8. The age of the participants ranged from 18 to 100 years. In the GWAS discovery set, comprising 38,292 participants for whole body lean mass and 28,330 participants for appendicular lean mass, a substantial excess of low *p*-values compared to the null distribution was observed after genomic control adjustment of the individual studies prior to meta-analysis: *λ*_GC_ = 1.076 and *λ*_GC_ = 1.075, for whole body and appendicular lean mass, respectively (Supplementary Fig. 1)

Meta-analyses were conducted using the METAL package (www.sph.umich.edu/csg/abecasis/metal/). We used the inverse variance weighting and fixed-effect model approach. Supplementary Table 1 shows the genome-wide significant (GWS) and suggestive (sGWS) results in the discovery set. For whole body lean mass, we observed one GWS result in/near *HSD17B11* and 12 sGWS results (in/near *VCAN*, *ADAMTSL3*, *IRS1*, *FTO* (*two SNPs*), *MOV10*, *HMCN1*, *RHOC*, *FRK*, *AKR1B1*, *CALCR*, and *KLF12*). For appendicular lean mass, one result was GWS (intronic SNP in *PKIB*) and seven were sGWS (in/near *VCAN*, *ADAMTSL3*, *HSD17B11*, *IRS1*, *FRK*, *TXN*, and *CTNNA3*).

We selected 21 associations (13 for whole body lean mass and 8 for appendicular lean mass; a total of 16 discovery SNPs with 5 SNPs overlapping between the two phenotypes) (Supplementary Table 1) to conduct a replication study in a set of 33 cohorts comprising up to 48,125 participants of European descent for whole body lean mass and 43,258 participants for appendicular lean mass. Both in silico replication and de novo genotyping for replication was conducted. Table 1 shows the results for successfully replicated SNPs in participants of European ancestry, including the discovery phase, replication phase, and the combined results.

**Table 1:** Results for the successfully replicated SNPs in discovery, replication and combined sample

SNP ID	Chrom	Position	Closest gene	Allele ½	EAF	Discovery (***n*** = 38,292)			Replication EU (***n*** = 47,227)			Combined EU (***n*** = 85,519)		
						Beta	SE	*p*-value	Beta	SE	*p*-value	Beta	SE	*p*-value
*Whole body lean mass*														
rs2943656	2	226830162	*IRSI*	A/G	0.38	−0.17	0.03	2.5 × 10^−7^	−0.13	0.03	8.0 × 10^−6^	−0.14	0.02	1.5 × 10^−11^
rs9991501	4	88477507	*HSD17B11*	T/C	0.04	−0.61	0.01	2.9 × 10^−8^	−0.26	0.08	1.9 × 10^−3^	−0.39	0.07	5.8 × 10^−9^
rs2287926	5	82851164	*VCAN*	A/G	0.12	0.24	0.05	8.6 × 10^−7^	0.15	0.04	8.5 × 10^−4^	0.19	0.03	7.5 × 10^−9^
rs4842924	15	82378611	*ADAMTSL3*	T/C	0.52	−0.17	0.03	1.4 × 10^−7^	−0.08	0.03	3.9 × 10^−3^	−0.12	0.02	1.4 × 10^−8^
rs9936385	16	52376670	*FTO*	T/C	0.61	−0.17	0.03	1.1 × 10^−6^	−0.11	0.03	1.6 × 10^−4^	−0.14	0.02	1.4 × 10^−9^

**SNP ID**	**Chrom**	**Position**	**Closest gene**	**Allele ½**	**EAF**	Discovery (***n*** = 28,330)			Replication EU (***n*** = 42,360)			Combined EU (***n*** = 70,690)		
						**Beta**	**SE**	***p*** **-value**	**Beta**	**SE**	***p*** **-value**	**Beta**	**SE**	***p*** **-value**
*Appendicular lean mass*														
rs2943656	2	226830162	*IRS1*	A/G	0.38	−0.10	0.02	1.1 × 10^−6^	−0.06	0.01	2.2 × 10^−5^	−0.07	0.01	2.9 × 10^−10^
rs2287926	5	82851164	*VCAN*	A/G	0.13	0.14	0.03	8.1 × 10^−7^	0.08	0.02	3.5 × 10^−4^	0.10	0.02	4.5 × 10^−9^
rs4842924	15	82378611	*ADAMTSL3*	T/C	0.52	−0.09	0.02	1.2 × 10^−6^	−0.05	0.02	1.6 × 10^−3^	−0.06	0.01	5.0 × 10^−8^

All results reflect analyses in participants of European ancestry

No significant heterogeneity was observed at *α* = 0.00625 (0.05/8)

Only mild heterogeneity was indicated in two associations for whole body lean mass when using an uncorrected threshold of *α* = 0.05, *FTO*/rs9936385 (*p* = 0.018, *I* ^2^ = 34%) and *HSD17B11*/ rs9991501 (*p* = 0.04, *I* ^2^ = 31%)

All results were adjusted for the following covariates: sex, age, height and fat mass (kg)

For whole body lean mass, joint analysis of the discovery and replication cohorts successfully replicated five SNPs in/near *HSD17B11*, *VCAN*, *ADAMTSL3, IRS1*, and *FTO* (*p*-values between 1.4 × 10^−8^ and 1.5 × 10^−11^ and lower than discovery *p*-values). Three of these five SNPs (located in/near *VCAN, ADAMTSL3*, and *IRS1*) were also successfully replicated (*p*-values between 5 × 10^−8^ and 2.9 × 10^−10^) for appendicular lean mass. None of the eight replicated associations (five for whole body and three appendicular lean mass) had significant heterogeneity at *α* = 0.00625 (0.05/8, Bonferroni-corrected for eight tests). Only mild heterogeneity was indicated in two whole body lean mass SNPs when using an uncorrected threshold of *α* = 0.05: FTO (*p* = 0.018, *I* ^2^ = 34%) and HSD17B11 (*p* = 0.04, *I* ^2^ = 31%). For appendicular lean mass, *p*-values for heterogeneity were >0.05 for all three replicated SNPs.

Supplementary Table 1 shows the results for all participants including those of non-European descent as well. Results were similar showing low heterogeneity using “transethnic meta-analysis” (MANTRA)^25^ for inclusion of both cohorts of European ancestry and replication cohorts with Asians or African Americans. Heterogeneity probability values were below 0.5 for all replicated SNPs both for whole body and appendicular lean mass. Furthermore, in this combined analysis, except for the *VCAN* locus, log_10_ Bayes’ factors were >6.0 and *p*-values were smaller than those found in European-only ancestry analysis.

Additional analyses stratified by sex failed to identify significant sex-specific associations or evidence of an interaction between SNPs and sex (Supplementary Table 2 and Supplementary Note 1). Similarly, we also found no evidence for heterogeneity between measurement techniques (BIA vs DXA; Supplementary Table 3 and Supplementary Note 2). Finally, we failed to replicate previously reported candidate genes for lean mass (Supplementary Note 2.4)

### Association with other anthropometric phenotypes

We looked for associations between the lead SNPs in the five replicated loci and other reported anthropometric phenotypes from the GIANT Consortium (Supplementary Table 10)^26–29^. There were no significant associations (*p* < 0.05) between the SNP in *HSD17B11* and any reported phenotypes. The allele associated with greater lean mass was associated with lower values of various anthropometric traits for the *IRS1* (hip circumference (HC), waist circumference (WC)), *VCAN* (hip ratio, WC adjusted for BMI, waist to hip ratio adjusted for BMI), and the *ADAMTSL3* locus (height, hip, and WC with the association becoming more significant for hip and waist adjusted for BMI). The replicated *FTO* SNP was very significantly associated with BMI (*p* = 2.7 × 10^−144^) and significantly associated with HC (*p* = 9.3 × 10^−81^) and WC (*p* = 1.3 × 10^−96^) in the same direction as the lean mass association (i.e., the higher lean mass allele was associated with higher values of anthropometric traits).

### Annotation and enrichment analysis of regulatory elements

Among five replicated SNPs, rs9991501 (*HSD17B11* locus) and rs2287926 (*VCAN* locus) are missense SNPs. rs2287926 was predicted as possibly damaging to the protein structure and/or function by PolyPhen-2^30^. Since the remaining GWAS SNPs are non-coding, to estimate whether these SNPs are located in regulatory elements in specific human tissue/cell types, we performed a tissue-specific regulatory-element enrichment analysis using experimental epigenetic evidence including DNAse hypersensitive sites, histone modifications, and transcription factor-binding sites in human cell lines and tissues from the ENCODE Project and the Epigenetic Roadmap Project. As shown in Table 2, 86 SNPs were in high LD (*r* ^2^ ≥ 0.8) with the GWAS lead SNP rs2943656 at the *IRS1* locus. The SNPs in this locus that were in high LD were enriched in enhancers estimated by ChromHMM^31^ (permutation *p*-values <0.05; after multiple testing corrections), especially enriched in fat and brain tissues, but not in skeletal muscle, smooth muscle, blood and gastrointestinal tract tissues in the ENCODE and Roadmap projects. Although the *IRS1* locus was not specifically enriched with skeletal muscle tissue enhancers, the lead SNP rs2943656 itself at the *IRS1* locus was actually located within a histone mark-identified promoter in an adult skeletal muscle sample and a histone mark-identified enhancer in several muscle samples (such as muscle satellite cultured cells, fetal skeletal muscle, skeletal muscle myoblasts, and skeletal muscle myotubes (Supplementary Figs. 6 and 7)). Based on the position weight matrices (PWMs) score from Chip-seq and other sequencing resources, rs2943656 was found to possibly alter regulatory motifs, including Irf, Foxo, Sox, and Zfp105 in skeletal muscle tissues with a PWM score *p*-value <4^−7^(≈6.1 × 10^−5^)^32^.

**Table 2:** Tissue-specific regulatory-element enrichment analyses of the GWAS loci (GWAS SNPs and SNPs in LD with the GWAS SNPs)

SNP ID	In/near gene	SNP functional role	Coding variant function by Polyphen2	Number of SNPs in LD^a^	*p*-value^b^ of tissue-specific regulatory element enrichment analyses^c^ in five tissues^d^					
					Skeletal muscle	Smooth muscle	Fat	Brain	Blood	Gastrointestinal tract
rs2943656	*IRS1*	Intergenic		86	0.14	0.38	1 × 10^−7^	0.04	0.82	1
rs9991501	*HSD17B11*	Exonic missense	Benign (Arg283Gln)	1	NA^e^	NA	NA	NA	NA	NA
rs2287926	*VCAN*	Exonic missense	Possibly damaging (Gly428Asp)	5	1 × 10^−7^	1 × 10^−7^	1 × 10^−7^	1 × 10^−7^	1	0.98
rs4842924	*ADAMTSL3*	Intronic		87	1 × 10^−7^	1 × 10^−7^	1 × 10^−7^	1 × 10^−7^	1	0.23
rs9936385	*FTO*	Intronic		91	0.78	0.38	0.49	0.45	1	0.51

^a^SNPs in LD: number of SNPs in LD (*r* ^2^ ≥ 0.8 and MAF ≥ 1%, based on CEU samples in the 1000 Genome Project) with the lead GWAS SNP in each locus

^b^Minimum *p*-value permutation tests: this analysis included all SNPs in LD with the GWS lead SNPs. Multiple testing correction was done by the minimum *p*-value permutation test. Permutation *p*-values <0.05 are considered as statistically significant.

^c^Enhancers and promoters (regulatory elements) in 25 chromatin states (retrieved from HaploReg4 database): SNPs are located within active regulatory elements, including promoter upstream TSS, promoter downstream TSS 1, promoter downstream TSS 2, transcribed and regulatory (prom/enh), transcribed 5ʹ preferential and enh, transcribed 3ʹ preferential and enh, transcribed and weak enhancer, active enhancer 1, active enhancer 2, active enhancer flank, weak enhancer 1, weak enhancer 2, primary H3K27ac possible enhancer, poised promoter, and bivalent promoter

^d^See Supplementary Note 2.6 for description of human primary cells and tissues that were included in each tissue group

^e^We did not perform enrichment analysis on rs9991501 because rs9991501 has no other SNPs in LD to obtain overlapping regulatory elements

For the *ADAMTSL3* locus, the GWAS lead SNP rs4842924 was located in a histone mark-identified enhancer in smooth muscle tissues (Supplementary Figs. 11 and 12). The promoter/enhancer enrichment analysis in the *ADAMTSL3* locus showed significant enrichments in skeletal muscle, smooth muscle, fat and brain tissues, but not in blood and gastrointestinal tract tissues. At the *FTO* locus, a few SNPs in high LD with the GWAS lead SNP, rs2287926, were located within a group of enhancers that are not muscle tissue-specific (Supplementary Fig. 13); therefore, no significant enrichment was found in any tissues listed in Table 2.

### Expression quantitative trait loci

We queried existing *cis*-expression quantitative trait loci (eQTLS) analyses on the five replicated GWAS SNPs rs2943656, rs9991501, rs2287926, rs4842924, and rs9936385 with transcripts within 2 Mb of the SNP position in skeletal muscle tissues as well as in subcutaneous adipose, omental adipose, liver tissue, lymphocytes, and primary osteoblasts (obtained from bone biopsies). Rs9936385 was associated with FTO expression in skeletal muscle tissues in the FUSION samples (*p* = 4.4 × 10^−11^) (Supplementary Table 10). However, in sequential conditional analysis, upon addition of the lead *FTO* eQTL SNP (rs11649091, association *p*-value with *FTO* gene expressio*n* = 5.1 × 10^−15^), rs9936385 was no longer significantly associated (*p* = 0.16), whereas rs11649091 remained significantly associated with *FTO* gene expression (*p* = 1.9 × 10^−5^). SNP rs11649091 could not be imputed using our HapMap imputation references; thus, we did not have association results between rs11649091 and lean mass in the present study. The T allele of rs9936385, associated with reduced lean mass in the present study, was significantly associated with lower *FTO* gene expression levels in the GTEx project (Supplementary Table 9 and Supplementary Fig. 14). For rs9936385, we also examined *IRX3* and *IRX5* expression in skeletal muscle tissues, as recent reports have implicated GWAS SNPs associated with obesity in intron 2 of the *FTO* gene as being associated with *IRX3* and *IRX5* gene expression in brain^33^ and adipose tissue^34^. SNP rs9936385 was not significantly associated with *IRX3* and *IRX5* gene expression in skeletal muscle tissues. For missense SNP rs9991501 in the *HSD17B11* locus, a significant association with *HSD17B11* gene expression was found in skeletal muscle from the GTEx project (*p* = 1.4 × 10^−4^). There were no significant GWAS SNPs in strong LD with this one; thus conditional analyses were not performed.

As shown in Supplementary Table 10, for GWAS lead SNP rs2943656 in the *IRS1* locus, significant eQTLs with the *IRS1* gene expression in omental (*p* = 4 × 10^−7^) and subcutaneous fat tissue (*p* = 6.44 × 10^−6^) were found.

Finally we found no evidence for differential expression of our five replicated genes in young vs old muscle biopsies (Supplementary Note 2.7 for methods and results).

## Discussion

In this first large-scale GWA meta-analysis study for lean mass that included most of the cohorts worldwide with lean mass phenotypes, we identified and successfully replicated five GWS loci (in/near *HSD17B11*, *VCAN, ADAMTSL3, IRS1*, and *FTO* genes) for whole body lean mass and three of these (in/near *VCAN, IRS1*, and *ADAMTSL3* genes) for appendicular lean mass, both important for sarcopenia diagnosis.

This study contributes to a better understanding of the biology underlying inter-individual variation in muscle mass, since lean body mass consists primarily of muscle mass (especially in the extremities). Genetic determinants of lean body mass cannot be studied specifically by using anthropometric measures such as height, waist circumference, hip circumferences, or BMI, as evidenced by our finding of associations between genetic loci and lean mass that were not observed in results from the GIANT consortium^26–29^. Four novel GWS loci for lean mass phenotypes harboring *ADAMTSL3, VCAN*, *HSD17B11*, and *IRS1* genes have biologic effects supporting their role in skeletal muscle. Although the functional involvement of the *ADAMTSL3* gene (a disintegrin-like and metalloprotease domain with thrombospondin type I motifs-like 3) remains unknown, it has been shown to be consistently associated with adult height in large human samples^26^, including individuals of African ancestry^35^. The gene is expressed ubiquitously, including in skeletal muscle but the lead GWAS SNP in this locus was not significantly associated with expression of *ADAMTSL3* in any of the tissues examined. One might hypothesize that our observed association between this gene and lean mass could be a reflection of an allometric relationship between muscle mass/size and body size^36^. Since our analyses were adjusted for height, it is plausible that variation in *ADAMTSL3* is associated with muscle mass directly. In fact, in a recent study that identified classes of potentially regulatory genomic elements that are enriched in GWAS loci, the height phenotype was almost exclusively enriched in DNAse sites in muscle^37^. In our study, we observed enrichment of regulatory genomic elements in this locus in both skeletal muscle and smooth muscle, which may not fully support a unique role of this gene in only skeletal muscle tissue.

Versican (VCAN) plays a role in intercellular signaling and in connecting cells with the extracellular matrix, which also is important for the skeletal muscle. Interestingly, VCAN facilitates chondrocyte differentiation and regulates joint morphogenesis^38^, and therefore has a wider role in the musculoskeletal health. The GWAS SNP in the *HSD17B11* locus was significantly associated with the *HSD17B11* gene expression in skeletal muscle. Hydroxysteroid (17-beta) dehydrogenase 11 (HSD17B11) functions in steroid biosynthesis, and most relevant to muscle tissue, contributes to androgen catabolic processes. Although not much is known about how variants in this gene associate with sex-hormone related human phenotypes, androgen metabolism is certainly a driver of muscle tissue anabolism and catabolism.

As for the *FTO* locus, variants in the *FTO* gene, which is known to regulate postnatal growth in mice^39^, have been found to be associated with adiposity/obesity^40^ and related traits, such as BMI^41^, metabolic syndrome/type 2 diabetes and even with menarche^42^. Apart from its role in adiposity, in two recent candidate gene studies, SNPs in *FTO* were found to associate both with DXA-derived fat and lean mass^43^, and in one, the association for lean mass was only slightly attenuated after fat mass adjustment^44^, similar to our results using fat mass-adjusted lean mass. Also, *FTO* knockout mice have been shown to have not only reduced fat mass, but also decrease in lean mass^45^. Recently, obesity associated non-coding sequences in the *FTO* gene were found to be functionally connected to the nearby gene, *IRX3*, by directly interacting with the promoters of this gene^33^, suggesting obesity SNPs located inside the *FTO* gene may regulate gene expression other than *FTO*. Our GWAS lead SNP rs9936385 in the *FTO* locus was in LD with the obesity GWAS SNP and located in the same haplotype. A denser fine-mapping study using sequencing of the *FTO* locus with a better resolution will be helpful in narrowing down the *FTO* region to identify potential causal variant(s). The actual function of the variants and the underlying mechanisms of *FTO*’s involvement in skeletal muscle biology still need to be further elucidated by in vitro and animal experiments.

The other body composition-related gene that was successfully replicated is the insulin receptor substrate 1 (*IRS1*), which belongs to the insulin signaling pathway and participates in growth hormone and adipocytokine signaling pathways. Besides being overexpressed in adipocytes, *IRS1* is also highly expressed in skeletal muscle^13^, *IRS1* polymorphisms have been associated with fasting insulin-related traits^46^, adiposity^13^, and serum triglycerides/HDL cholesterol^47^. Our GWAS lead SNP rs2943656 was associated with *IRS1* gene expression in skeletal muscle obtained from the GTEx project. Interestingly, another SNP, rs2943650, near *IRS1* in high LD with our lead SNP (*R* ^2^ = 0.854) was previously found to be associated with percent body fat in the opposite direction from the association that we find with lean mass^13^. The body fat percentage decreasing allele in this study was associated with lower IRS1 expression in omental and subcutaneous fat^13^. Another study reported an association between a SNP in high LD (rs2943641) with IRS-1 protein expression and insulin-induced phosphatidylinositol 3-OH kinase activity in skeletal muscle^13^. Also rs2943656 was associated with obesity traits in the GIANT Consortium; the allele associated with greater lean mass was inversely associated with obesity traits. Further understanding of the potential functional effects of these variants in the *IRS1* locus are needed to determine whether they have pleiotropic and opposite effects on fat and lean tissue. The same is true for variants in the *FTO* locus for which the allele that was associated with greater lean mass was previously reported to be associated with greater fat mass.

From the current eQTL data analysis, we have no definitive evidence that the non-coding GWAS SNPs (or variants in high LD) functionally influence gene expression of *IRS1*, *HSD17B1*, and *FTO* in skeletal muscle. Use of a larger reference panel for imputation in the GWAS sample, and analysis of larger tissue expression data sets coupled with conditional analysis will help reveal if underlying functional associations exist. It should be emphasized that with the current data we are not able to determine which SNPs may be functional and it is possible that the identified lean mass variants are not driving the associations with expression. Using ENCODE and Epigenetic Roadmap data, we found that the GWAS SNPs (or SNPs in LD with these SNPs) were significantly enriched in the predicted gene regulatory regions (in our case, enhancers) not only in skeletal muscle, but also in smooth muscle, fat and brain tissues. With the complexity of lean mass phenotypes, we cannot rule out the possibility that these genes are involved in regulating lean mass biology via tissues other than skeletal muscle.

Using results from the overall meta-analysis, the percent variance explained by the successfully replicated SNPs was 0.23% and 0.16% for whole body and appendicular lean mass, respectively. Estimates were slightly higher when we used individual level data from the Framingham Study cohorts (percent variance explained of 0.97% and 0.55% for whole body and appendicular lean mass, respectively). This relatively small percentage of explained variance is not dissimilar to other body composition measures such as bone mineral density of the femoral neck, for which 63 SNPs explained 5.8% of the variance^48^. To estimate the proportion of variance for lean mass explained by all genotyped SNPs across the genome in Framingham Study participants, we applied a GREML model implemented in the GCTA package^49^ with the assumption that all 550K genotyped SNPs captured ≥80% of the common sequence variance in the Framingham Study participants who are Caucasians of European ancestry. We estimated that the proportion of whole body lean mass and appendicular lean mass variance explained by all genotyped SNPs together was 43.3% (SE: 2.7%) and 44.2% (SE: 2.6%), respectively (after adjustment for age, age^2^, sex, height and fat mass), suggesting most of the heritability of lean mass was not detected in the current study due to the limitations of study design (only common variants in this study). This percent variance is similar to the 45% of the variance explained for height using this method^49^.

Because of the substantial sexual dimorphism in body composition with men having higher muscle mass compared with women^24^, we performed both sex-combined and sex-stratified analyses. We examined potential sex differences in the genetic associations. Lean mass is a highly heritable trait and the heritability is of similar magnitude in both genders^24, 50^. No formal interaction test between SNP and sex was significant for any of these SNPs. Thus, our findings do not support any substantial sex-specific genetic influence for any of the successfully replicated lean mass SNPs. We cannot rule out the possibility that the reported GWS SNPs are false-positive findings, although the chance of such false positives is extremely low due to the robustness of replication in our well-powered study. There are also other limitations to our study. Because lean mass is correlated with fat mass, we adjusted for fat mass to focus our search on genes contributing to lean mass independent of those regulating fat mass. A potential limitation with this strategy of adjusting for fat mass is that the power to identify genetic signals with a similar impact on lean and fat mass will be reduced. Nevertheless, the *FTO* signal was found to be significantly associated with lean mass after fat adjustment and the direction of this association was the same as the association with fat mass. Since androgens have a major impact on muscle mass, it is a limitation of the present study that the X chromosome, harboring the androgen receptor gene, was not included in the present meta-analysis. Another potential weakness of this study is our decision to meta-analyze body composition results using two different techniques (BIA and DXA). Nevertheless, the two methods are highly correlated (*r* = 0.83 for Framingham cohort participants), and by combining them power to detect GWS loci was greatly enhanced.

In conclusion, in this first large-scale meta-analysis of GWAS, five GWS variants in or near the *HSD17B11*, *VCAN*, *ADAMTSL3*, *IRS1*, and *FTO* genes were found to be robustly associated with lean body mass. Three of these loci were found to be significantly enriched in enhancers and promoters in muscle cells, suggesting that our signals have a potential functional role in muscle. Our findings shed light on pathophysiological mechanisms underlying lean mass variation and potential complex interrelations between the genetic architecture of muscle mass, fat mass, body height and metabolic disease.

## Methods

### Study summary

We focused on two phenotypes: (1) whole body lean mass; (2) appendicular lean mass. For these two phenotypes, we performed a genome-wide meta-analysis of the discovery cohorts (Stage I), then meta-analyzed the genotyped discovery SNPs in replication cohorts (Stage II), followed by a combined analysis with discovery and replication cohorts (Supplementary Fig. 1). The total sample size for the combined analysis was just over 100,000 from 53 studies. Our primary results are based on the ~85,000 individuals of European descent in 47 studies, as was pre-specified. Because whole body and appendicular lean mass are correlated with fat mass and height, analyses were adjusted for these potential confounders in addition to sex, age, age^2^, and other study-specific covariates to focus our search on genes contributing to lean mass independent of those of body height and fat mass.

### Study population

The Stage I Discovery sample comprised 38,292 individuals of European ancestry drawn from 20 cohorts with a variety of epidemiological designs and participant characteristics (Supplementary Tables 4–6 and Supplementary Note 2.8). Whole body lean mass was measured using DXA (10 cohorts, *n* = 21,074) and BIA (10 cohorts, *n* = 17,218). Of the 20 cohorts, 15 consisted of male and female subjects, while 2 had male and 3 had female subjects only. In total, the cohorts included 22,705 women and 15,587 men. Appendicular lean mass was estimated in 28,330 subjects from a subset of 15 cohorts (9 using DXA and 6 using BIA).

Subjects from 33 additional studies were used for replication with a total sample size of 63,475 individuals. Of these 63,475, the majority was of European ancestry (*n* = 47,227 in 27 cohorts), and the remaining 16,248 were of African American, South Asian, or Korean ancestry (Supplementary Table 4). All these 33 cohorts had data for whole body lean mass. Among them, 16 studies had DXA measurements (*n* = 23,718) and 17 studies had BIA measurements (*n* = 39,757). Twenty-five cohorts had data for appendicular lean mass in a total of 45,090 individuals (16 cohorts with DXA (*n* = 23,718) and 9 with BIA (*n* = 21,372)). Of these, 42,360 individuals (23 cohorts) were of European ancestry and the remaining 2730 of African American and Korean descent. Our a priori aim was to perform replication in cohorts with European subjects only and to explore if adding non-European cohorts would increase power or show evidence of heterogeneity due to ethnicity. The Stage II Replication included cohorts with existing GWAS data that were unavailable at the time of the Stage I Discovery, and cohorts who agreed to undergo de novo genotyping. All studies were approved by their institutional ethics review committees and all participants provided written informed consent.

### Lean mass measurements

Lean mass was measured in all cohorts using either DXA or BIA. DXA provides body composition as three materials based on specific X-ray attenuation properties; bone mineral, lipid (triglycerides, phospholipid membranes, etc.) and lipid-free soft tissue. For each pixel on the DXA scan, these three materials are quantified. For the cohorts with DXA measures, the phenotype used for these analyses was the lipid free, soft tissue compartment that is referred to as lean mass, and is the sum of body water, protein, glycerol, and soft tissue mineral mass. Two lean mass phenotypes were used: whole body lean mass and appendicular lean mass. The latter was obtained by considering only pixels in the arms and legs collectively, which has been demonstrated to be a valid measure of skeletal muscle mass^51^.

Some of the cohorts estimated body composition using BIA, which relies on the geometrical relationship between impedance (*Z*), length (*L*), and volume (*V*) of an electrical conductor. Adapted to the human body, *V* corresponds to the volume of fat-free mass (FFM) and *L* to the height of the subject. *Z* is composed of the pure resistance (*R*) of the conductor, the FFM, and the reactance (Xc), produced by the capacitance of cellular membranes, tissue interfaces and non-ionic tissues: *Z* ^2^ = *R* ^2^ + Xc^2^. A variety of BIA machines were used by the various cohorts (summarized in Supplementary Table 5), and in some cohorts, the specific resistance and reactance measures were not available because the manufacturers provided only summary output on FFM. For BIA cohorts with specific resistance and reactance measures, we used the validated equation from Kyle et al.^52^ with an *R* ^2^ of 0.95 between BIA and DXA to calculate the appendicular lean mass.

### Stage 1: genome-wide association analyses in discovery cohorts

#### Genotyping and imputation

Genome-wide genotyping was done by each study on a variety of platforms following standard manufacturer protocols. Quality control was performed independently for each study. To facilitate meta-analysis, each group performed genotype imputation with IMPUTE^53^ or MACH^54^ software using HapMap Phase II release 22 reference panels (CEU or CHB/JPT as appropriate). Overall imputation quality scores for each SNP were obtained from IMPUTE (“proper_info”) or MACH (“rsq_hat”). Details on the genotyping platform used, genotype quality control procedures and software for imputation employed for each study are presented in Supplementary Table 6.

#### Study-specific genome-wide association analyses with lean mass

In each study, a multiple linear regression model with additive genetic effect was applied to test for phenotype–genotype association using ~2.0 to 2.5 million genotyped and/or imputed autosomal SNPs. Other covariates adjusted in the model included ancestral genetic background, sex, age, age^2^, height, fat mass measured by the body composition device (kg) and study-specific covariates when appropriate such as clinical center for multi-center cohorts. Adjustment for ancestral background was done within cohorts using principal component analyses as necessary. Furthermore, for family-based studies, including the Framingham Study, ERF, UK-Twins, Old Order Amish Study and the Indiana cohort, familial relatedness was taken into account in the statistical analysis within their cohorts by: (1) linear mixed-effects models that specified fixed genotypic and covariate effects and a random polygenic effect to account for familial correlations (the R Kinship package; http://cran.r-project.org/web/packages/) in the Framingham Study; (2) the GenABEL^55^ in the ERF and UK-Twins cohorts; (3) the Mixed Model Analysis for Pedigrees (MMAP) program (http://edn.som.umaryland.edu/mmap/index.php) in the Amish cohort; and GWAF, an R package for genome-wide association analyses with family data in the Indiana cohort^56^.

#### Meta-analyses

Meta-analyses were conducted using the METAL package (www.sph.umich.edu/csg/abecasis/metal/). We used the inverse variance weighting and fixed-effect model approach. Prior to meta-analysis, we filtered out SNPs with low minor allele frequency, MAF (<1%) and poor imputation quality (proper_info<0.4 for IMPUTE and rsq_hat<0.3) and applied genomic control correction where the genomic control parameter lambda (*λ*_GC_) was >1.0.

We used quantile–quantile (Q–Q) plots of observed vs. expected –log_10_ (*p*-value) to examine the genome-wide distribution of *p*-values for signs of excessive false-positive results. We generated Manhattan plots to report genome-wide *p*-values, regional plots for genomic regions within 100 Kb of top hits, and forest plots for meta-analyses and study-specific results of the most significant SNP associations. A threshold of *p* < 5 × 10^−8^ was pre-specified as being genome-wide significant (GWS), while a threshold of *p* < 2.3 × 10^−6^ was used to select SNPs for a replication study (suggestive genome-wide significant, sGWS).

### Stage 2: replication

In each GWS or sGWS locus, we selected the lead SNP with the lowest *p*-value for replication. In addition, GWS or sGWS SNPs that had low-linkage disequilibrium with the lead SNPs (LD < 0.5) were also selected for replication. Both in silico replication and de novo genotyping for replication was conducted. In silico replication was done in 24 cohorts with GWAS SNP chip genotyping that did not have data available at the time of the initial discovery efforts (Supplementary Table 7). De-novo replication genotyping was done using: KBioScience Allele-Specific Polymorphism (KASP) SNP genotyping system (in OPRA, PEAK25, AGES, CAIFOS, DOPS cohorts), TaqMan (METSIM), Illumina OmniExpress + Illumina Metabochip (PIVUS and ULSAM), or Sequenom’s iPLEX (WHI) (Supplementary Table 8). Samples and SNPs that did not meet the quality control criteria defined by each individual study were excluded. Minimum genotyping quality-control criteria were defined as: SNP call rate > 90% and Hardy–Weinberg equilibrium *p* > 1 × 10^−4^.

### Meta-analysis of replication and discovery studies

In the replication stage, we meta-analyzed results from: (1) individuals of European descent only (Rep-EUR); and (2) all replication cohorts with multiple ethnicities (Rep-All). Likewise we meta-analyzed results from discovery cohorts and European-descent-only replication cohorts (“Combined EUR”) from discovery cohorts and all replication cohorts (“Combined All”). To investigate and account for potential heterogeneities in allelic effects between studies, we also performed “trans-ethnic meta-analysis” using MANTRA^25^ in the replication sample that included all ethnic groups (“Rep-All”) and in the combined analysis of the discovery and all ethnic groups in the replication sample (“Combined All”).

A successful replication was considered if: (1) the association *p*-value in the cumulative-meta-analysis (Combined EUR) was genome-wide significant (*p* < 5 × 10^−8^) and less than the discovery meta-analysis *p*-value; or (2) the association *p*-value in the meta-analysis of replication-cohorts only (Rep-EUR) was less than *p* = 0.0024 (a Bonferroni-adjusted threshold at *p* = 0.05/21 since there were a total of 21 tests performed for whole body and appendicular lean mass in Rep-EUR cohorts during replication). Using the METAL package we also estimated *I* ^2^ to quantify heterogeneity and *p*-values to assess statistical significance for a total of eight associations that were replicated in the cumulative-meta-analysis (combined EUR, five SNPs for whole body and three for appendicular lean mass).

To estimate the phenotypic variance explained by the genotyped SNPs in the Framingham Heart Study (FHS), we used a restricted maximum likelihood model implemented in the GCTA (Genome-wide Complex Trait Analysis) tool package^57, 58^ and adjusted for the same set of covariates included in our GWAS.

Finally, we examined associations between all imputed SNPs in/near five genes (*THRH*, *GLYAT*, *GREM1*, *CNTF*, and *PRDM16* including 60 kB up and downstream of the gene) and lean mass, as these genes have been implicated to have associations with lean mass in previous association studies^18–20^.

### Annotation and enrichment analysis of regulatory elements

For coding variants, we predicted their function by PolyPhen-2. For all variants, we annotated potential regulatory functions of our replicated GWAS SNPs and loci based on experimental epigenetic evidence including DNAse hypersensitive sites, histone modifications, and transcription factor-binding sites in human cell lines and tissues from the ENCODE Project and the Epigenetic Roadmap Project. We first selected SNPs in high LD (*r* ^2^ ≥ 0.8) with GWAS lead SNPs based on the approach of Trynka et al.^59^ We then identified potential enhancers and promoters in the GWAS loci (GWAS SNPs and SNPs in LD with the GWAS SNPs) across 127 healthy human tissues/normal cell lines available in the ENCODE Project and the Epigenetic Roadmap Project from the HaploReg4 web browser^60^ using ChromHMM^31^. To evaluate whether replicated GWAS loci were enriched with regulatory elements in skeletal muscle tissue, we performed a hypergeometric test. Specifically we tested whether estimated tissue-specific promoters and enhancers in a GWAS locus were enriched in eight relevant skeletal muscle tissues/cell lines vs enrichment in non-skeletal muscle tissues (119 tissues/cell lines). The permutation with minimum *p*-value approach was performed to correct for multiple testing. Permutation *p*-values <0.05 were considered statistically significant. In addition, we also performed enrichment analyses in smooth muscle tissues/cells, fat tissue, brain, blood cells and gastrointestinal tract tissues. The eight skeletal muscle relevant tissues/cells were excluded when conducting enrichment analyses for other tissue types. The detailed information for tissue types and chromatin state estimation is described in the Supplementary Materials.

### *cis*-eQTL

We conducted *cis*-eQTL analyses on the five replicated GWS loci, SNPs rs2943656, rs9991501, rs2287926, rs4842924, and rs9936385, with gene expression within 2 Mb of the SNP position. A linear regression model was applied to examine associations between SNP and gene expression. The eQTL analyses were performed in five studies with available human skeletal muscle tissues, including: GTEx^61^, STRRIDE^62, 63^, a study with chest wall muscle biopsies from patients who underwent thoracic surgery for lung and cardiac diseases^64^, the Finland-United States Investigation of NIDDM Genetics (FUSION) Study^65^, and a study of Pima Indians^66^. In addition, eQTL analyses were also conducted in studies with other human tissues, including subcutaneous adipose^67, 68^, omental adipose^67, 68^, liver tissue^67^, lymphocytes^69^, and primary osteoblasts^70^ (obtained from bone biopsies). These five GWAS SNPs were either genotyped or imputed in each sample. The detailed methods are described in the Supplementary Materials. Multiple testing was corrected by using false discovery rate (FDR *q*-value <0.05) to account for all pairs of SNP-gene expression analyses in multiple tissues and studies.

### Data availability

All relevant data are available from the authors and summary level results are available on dbGaP.

## Electronic supplementary material

Supplementary Information

## References

[1] Janssen I (2006). Influence of sarcopenia on the development of physical disability: the Cardiovascular Health Study. J. Am. Geriatr. Soc..

[2] Janssen I, Heymsfield SB, Ross R (2002). Low relative skeletal muscle mass (sarcopenia) in older persons is associated with functional impairment and physical disability. J. Am. Geriatr. Soc..

[3] Visser M (2002). Leg muscle mass and composition in relation to lower extremity performance in men and women aged 70 to 79: the health, aging and body composition study. J. Am. Geriatr. Soc..

[4] Studenski SA (2014). The FNIH sarcopenia project: rationale, study description, conference recommendations, and final estimates. J. Gerontol. A Biol. Sci. Med. Sci..

[5] Houston DK (2008). Dietary protein intake is associated with lean mass change in older, community-dwelling adults: the Health, Aging, and Body Composition (Health ABC) Study. Am. J. Clin. Nutr..

[6] Lillioja S, Bogardus C (1988). Obesity and insulin resistance: lessons learned from the Pima Indians. Diabetes Metab. Rev..

[7] Devaney JM, Tosi LL, Fritz DT (2009). Differences in fat and muscle mass associated with a functional human polymorphism in a post-transcriptional BMP2 gene regulatory element. J. Cell Biochem..

[8] Janssen I, Baumgartner RN, Ross R, Rosenberg IH, Roubenoff R (2004). Skeletal muscle cutpoints associated with elevated physical disability risk in older men and women. Am. J. Epidemiol..

[9] Janssen I, Shepard DS, Katzmarzyk PT, Roubenoff R (2004). The healthcare costs of sarcopenia in the United States. J. Am. Geriatr. Soc..

[10] Arden NK, Spector TD (1997). Genetic influences on muscle strength, lean body mass, and bone mineral density: a twin study. J. Bone Miner. Res..

[11] Hsu FC (2005). Heritability of body composition measured by DXA in the diabetes heart study. Obes. Res..

[12] Berndt SI (2013). Genome-wide meta-analysis identifies 11 new loci for anthropometric traits and provides insights into genetic architecture. Nat. Genet..

[13] Kilpelainen TO (2011). Genetic variation near IRS1 associates with reduced adiposity and an impaired metabolic profile. Nat. Genet..

[14] Monda KL (2013). A meta-analysis identifies new loci associated with body mass index in individuals of African ancestry. Nat. Genet..

[15] Randall JC (2013). Sex-stratified genome-wide association studies including 270,000 individuals show sexual dimorphism in genetic loci for anthropometric traits. PLoS Genet..

[16] Chagnon YC, Borecki IB, Perusse L (2000). Genome-wide search for genes related to the fat-free body mass in the Quebec family study. Metabolism.

[17] Livshits G, Kato BS, Wilson SG, Spector TD (2007). Linkage of genes to total lean body mass in normal women. J. Clin. Endocrinol. Metab..

[18] Liu XG (2009). Genome-wide association and replication studies identified TRHR as an important gene for lean body mass. Am. J. Hum. Genet..

[19] Guo YF (2013). Suggestion of GLYAT gene underlying variation of bone size and body lean mass as revealed by a bivariate genome-wide association study. Hum. Genet..

[20] Hai R (2012). Genome-wide association study of copy number variation identified gremlin1 as a candidate gene for lean body mass. J. Hum. Genet..

[21] Urano T, Shiraki M, Sasaki N, Ouchi Y, Inoue S (2014). Large-scale analysis reveals a functional single-nucleotide polymorphism in the 5’-flanking region of PRDM16 gene associated with lean body mass. Aging Cell.

[22] Lee RC, Wang ZM, Heymsfield SB (2001). Skeletal muscle mass and aging: regional and whole-body measurement methods. Can. J. Appl. Physiol..

[23] Heymsfield SB, Gallagher D, Visser M, Nunez C, Wang ZM (1995). Measurement of skeletal muscle: laboratory and epidemiological methods. J. Gerontol. A Biol. Sci. Med. Sci..

[24] Zillikens MC (2008). Sex-specific genetic effects influence variation in body composition. Diabetologia.

[25] Morris AP (2011). Transethnic meta-analysis of genomewide association studies. Genet. Epidemiol..

[26] Lango Allen H (2010). Hundreds of variants clustered in genomic loci and biological pathways affect human height. Nature.

[27] Lindgren CM (2009). Genome-wide association scan meta-analysis identifies three loci influencing adiposity and fat distribution. PLoS Genet..

[28] Winkler TW (2015). The influence of age and sex on genetic associations with adult body size and shape: a large-scale genome-wide interaction study. PLoS Genet..

[29] Wood AR (2014). Defining the role of common variation in the genomic and biological architecture of adult human height. Nat. Genet..

[30] Adzhubei IA (2010). A method and server for predicting damaging missense mutations. Nat. Methods.

[31] Ernst J, Kellis M (2012). ChromHMM: automating chromatin-state discovery and characterization. Nat. Methods.

[32] Touzet H, Varre JS (2007). Efficient and accurate P-value computation for position weight matrices. Algorithms Mol. Biol..

[33] Smemo S (2014). Obesity-associated variants within FTO form long-range functional connections with IRX3. Nature.

[34] Claussnitzer M (2015). FTO obesity variant circuitry and adipocyte browning in humans. N. Engl. J. Med..

[35] N’Diaye A (2011). Identification, replication, and fine-mapping of loci associated with adult height in individuals of african ancestry. PLoS Genet..

[36] Handsfield GG, Meyer CH, Hart JM, Abel MF, Blemker SS (2014). Relationships of 35 lower limb muscles to height and body mass quantified using MRI. J. Biomech..

[37] Pickrell JK (2014). Joint analysis of functional genomic data and genome-wide association studies of 18 human traits. Am. J. Hum. Genet..

[38] Choocheep K (2010). Versican facilitates chondrocyte differentiation and regulates joint morphogenesis. J. Biol. Chem..

[39] Gao X (2010). The fat mass and obesity associated gene FTO functions in the brain to regulate postnatal growth in mice. PLoS ONE.

[40] Hinney A (2007). Genome wide association (GWA) study for early onset extreme obesity supports the role of fat mass and obesity associated gene (FTO) variants. PLoS ONE.

[41] Yang J (2012). FTO genotype is associated with phenotypic variability of body mass index. Nature.

[42] Elks CE (2010). Thirty new loci for age at menarche identified by a meta-analysis of genome-wide association studies. Nat. Genet..

[43] Livshits G, Malkin I, Moayyeri A, Spector TD, Hammond CJ (2012). Association of FTO gene variants with body composition in UK twins. Ann. Hum. Genet..

[44] Sonestedt E (2011). Association between fat intake, physical activity and mortality depending on genetic variation in FTO. Int. J. Obes..

[45] Fischer J (2009). Inactivation of the Fto gene protects from obesity. Nature.

[46] Manning AK (2012). A genome-wide approach accounting for body mass index identifies genetic variants influencing fasting glycemic traits and insulin resistance. Nat. Genet..

[47] Sharma R (2011). The type 2 diabetes and insulin-resistance locus near IRS1 is a determinant of HDL cholesterol and triglycerides levels among diabetic subjects. Atherosclerosis.

[48] Estrada K (2012). Genome-wide meta-analysis identifies 56 bone mineral density loci and reveals 14 loci associated with risk of fracture. Nat. Genet..

[49] Yang J (2010). Common SNPs explain a large proportion of the heritability for human height. Nat. Genet..

[50] Schousboe K (2004). Twin study of genetic and environmental influences on adult body size, shape, and composition. Int. J. Obes. Relat. Metab. Disord..

[51] Chen Z (2007). Dual-energy X-ray absorptiometry is a valid tool for assessing skeletal muscle mass in older women. J. Nutr..

[52] Kyle UG, Genton L, Hans D, Pichard C (2003). Validation of a bioelectrical impedance analysis equation to predict appendicular skeletal muscle mass (ASMM). Clin. Nutr..

[53] Howie BN, Donnelly P, Marchini J (2009). A flexible and accurate genotype imputation method for the next generation of genome-wide association studies. PLoS Genet..

[54] Li Y, Abecasis GR (2006). Mach 1.0: rapid haplotype reconstruction and missing genotype inference. Am. J. Hum. Genet..

[55] Aulchenko YS, Ripke S, Isaacs A, van Duijn CM (2007). GenABEL: an R library for genome-wide association analysis. Bioinformatics.

[56] Chen MH, Yang Q (2010). GWAF: an R package for genome-wide association analyses with family data. Bioinformatics.

[57] Yang B, Sun H, Wang H (2010). The downstream effects of vitamin D in spermatozoa needs further study. Hum. Reprod..

[58] Yang J, Lee SH, Goddard ME, Visscher PM (2011). GCTA: a tool for genome-wide complex trait analysis. Am. J. Hum. Genet..

[59] Trynka G (2013). Chromatin marks identify critical cell types for fine mapping complex trait variants. Nat. Genet..

[60] Ward LD, Kellis M (2012). HaploReg: a resource for exploring chromatin states, conservation, and regulatory motif alterations within sets of genetically linked variants. Nucleic Acids Res..

[61] Consortium GT (2013). The Genotype-Tissue Expression (GTEx) project. Nat. Genet..

[62] Kraus WE (2001). Studies of a targeted risk reduction intervention through defined exercise (STRRIDE). Med. Sci. Sports Exerc..

[63] Slentz CA (2011). Effects of aerobic vs. resistance training on visceral and liver fat stores, liver enzymes, and insulin resistance by HOMA in overweight adults from STRRIDE AT/RT. Am. J. Physiol. Endocrinol. Metab..

[64] Schadt E. A data driven approach to diagnosing and treating disease. In *Proc. 20th ACM SIGKDD International Conference on Knowledge Discovery and Data Mining*. 3 (ACM, 2014).

[65] Valle T (1998). Mapping genes for NIDDM. Design of the Finland-United States Investigation of NIDDM Genetics (FUSION) Study. Diabetes Care.

[66] Hanson RL (2014). A genome-wide association study in American Indians implicates DNER as a susceptibility locus for type 2 diabetes. Diabetes.

[67] Schadt EE (2008). Mapping the genetic architecture of gene expression in human liver. PLoS Biol..

[68] Greenawalt DM (2011). A survey of the genetics of stomach, liver, and adipose gene expression from a morbidly obese cohort. Genome Res..

[69] Dixon AL (2007). A genome-wide association study of global gene expression. Nat. Genet..

[70] Grundberg E (2009). Population genomics in a disease targeted primary cell model. Genome Res..

