# Erratum: Large meta-analysis of genome-wide association studies identifies five loci for lean body mass

**DOI:** 10.1038/s41467-017-01008-2

**Published:** 2017-11-07

**Authors:** M. Carola Zillikens, Serkalem Demissie, Yi-Hsiang Hsu, Laura M. Yerges-Armstrong, Wen-Chi Chou, Lisette Stolk, Gregory Livshits, Linda Broer, Toby Johnson, Daniel L. Koller, Zoltán Kutalik, Jian’an Luan, Ida Malkin, Janina S. Ried, Albert V. Smith, Gudmar Thorleifsson, Liesbeth Vandenput, Jing Hua Zhao, Weihua Zhang, Ali Aghdassi, Kristina Åkesson, Najaf Amin, Leslie J. Baier, Inês Barroso, David A. Bennett, Lars Bertram, Rainer Biffar, Murielle Bochud, Michael Boehnke, Ingrid B. Borecki, Aron S. Buchman, Liisa Byberg, Harry Campbell, Natalia Campos Obanda, Jane A. Cauley, Peggy M. Cawthon, Henna Cederberg, Zhao Chen, Nam H. Cho, Hyung Jin Choi, Melina Claussnitzer, Francis Collins, Steven R. Cummings, Philip L. De Jager, Ilja Demuth, Rosalie A. M. Dhonukshe-Rutten, Luda Diatchenko, Gudny Eiriksdottir, Anke W. Enneman, Mike Erdos, Johan G. Eriksson, Joel Eriksson, Karol Estrada, Daniel S. Evans, Mary F. Feitosa, Mao Fu, Melissa Garcia, Christian Gieger, Thomas Girke, Nicole L. Glazer, Harald Grallert, Jagvir Grewal, Bok-Ghee Han, Robert L. Hanson, Caroline Hayward, Albert Hofman, Eric P. Hoffman, Georg Homuth, Wen-Chi Hsueh, Monica J. Hubal, Alan Hubbard, Kim M. Huffman, Lise B. Husted, Thomas Illig, Erik Ingelsson, Till Ittermann, John-Olov Jansson, Joanne M. Jordan, Antti Jula, Magnus Karlsson, Kay-Tee Khaw, Tuomas O. Kilpeläinen, Norman Klopp, Jacqueline S. L. Kloth, Heikki A. Koistinen, William E. Kraus, Stephen Kritchevsky, Teemu Kuulasmaa, Johanna Kuusisto, Markku Laakso, Jari Lahti, Thomas Lang, Bente L. Langdahl, Lenore J. Launer, Jong-Young Lee, Markus M. Lerch, Joshua R. Lewis, Lars Lind, Cecilia Lindgren, Yongmei Liu, Tian Liu, Youfang Liu, Östen Ljunggren, Mattias Lorentzon, Robert N. Luben, William Maixner, Fiona E. McGuigan, Carolina Medina-Gomez, Thomas Meitinger, Håkan Melhus, Dan Mellström, Simon Melov, Karl Michaëlsson, Braxton D. Mitchell, Andrew P. Morris, Leif Mosekilde, Anne Newman, Carrie M. Nielson, Jeffrey R. O’Connell, Ben A. Oostra, Eric S. Orwoll, Aarno Palotie, Stephen C. J. Parker, Munro Peacock, Markus Perola, Annette Peters, Ozren Polasek, Richard L. Prince, Katri Räikkönen, Stuart H. Ralston, Samuli Ripatti, John A. Robbins, Jerome I. Rotter, Igor Rudan, Veikko Salomaa, Suzanne Satterfield, Eric E. Schadt, Sabine Schipf, Laura Scott, Joban Sehmi, Jian Shen, Chan Soo Shin, Gunnar Sigurdsson, Shad Smith, Nicole Soranzo, Alena Stančáková, Elisabeth Steinhagen-Thiessen, Elizabeth A. Streeten, Unnur Styrkarsdottir, Karin M. A. Swart, Sian-Tsung Tan, Mark A. Tarnopolsky, Patricia Thompson, Cynthia A. Thomson, Unnur Thorsteinsdottir, Emmi Tikkanen, Gregory J. Tranah, Jaakko Tuomilehto, Natasja M. van Schoor, Arjun Verma, Peter Vollenweider, Henry Völzke, Jean Wactawski-Wende, Mark Walker, Michael N. Weedon, Ryan Welch, H.-Erich Wichmann, Elisabeth Widen, Frances M. K. Williams, James F. Wilson, Nicole C. Wright, Weijia Xie, Lei Yu, Yanhua Zhou, John C. Chambers, Angela Döring, Cornelia M. van Duijn, Michael J. Econs, Vilmundur Gudnason, Jaspal S. Kooner, Bruce M. Psaty, Timothy D. Spector, Kari Stefansson, Fernando Rivadeneira, André G. Uitterlinden, Nicholas J. Wareham, Vicky Ossowski, Dawn Waterworth, Ruth J. F. Loos, David Karasik, Tamara B. Harris, Claes Ohlsson, Douglas P. Kiel

**Affiliations:** 1 Department of Internal Medicine, Erasmus MC, Rotterdam, 3000 The Netherlands; 2 Netherlands Genomics Initiative (NGI)-sponsored Netherlands Consortium for Healthy Aging (NCHA), Leiden, 2593 The Netherlands; 3 Department of Biostatistics, Boston University School of Public Health, Boston, MA 02118 USA; 4 Hebrew SeniorLife, Institute for Aging Research, Roslindale, MA 02131 USA; 5 Harvard Medical School, Boston, MA 02115 USA; 6 Molecular and Integrative Physiological Sciences Program, Harvard School of Public Health, Boston, MA 02115 USA; 7 Program in Personalized and Genomic Medicine, and Department of Medicine, Division of Endocrinology, Diabetes and Nutrition, University of Maryland School of Medicine, Baltimore, MD 21201 USA; 8 Broad Institute, Cambridge, MA 02142 USA; 9 Sackler Faculty of Medicine, Department of Anatomy and Anthropology, Tel Aviv University, Tel Aviv, 6997801 Israel; 10 Department of Twin Research and Genetic Epidemiology, King’s College London, St Thomas’ Campus, London, WC2R 2LS UK; 11 Department of Epidemiology, Erasmus MC, Rotterdam, 3000 The Netherlands; 12 Department of Medical Genetics, University of Lausanne, Lausanne, 1011 Switzerland; 13 Swiss Institute of Bioinformatics, Lausanne, 1015 Switzerland; 14 Centre Hospitalier Universitaire (CHUV), University Institute for Social and Preventive Medicine, Lausanne, 1010 Switzerland; 15 Department of Medical and Molecular Genetics, Indiana University School of Medicine, Indianapolis, IN 46202 USA; 16 MRC Epidemiology Unit, University of Cambridge School of Clinical Medicine, Cambridge Biomedical Campus, Cambridge, CB2 OQQ UK; 17 Institute of Epidemiology II, Helmholtz Zentrum München – German Research Center for Environmental Health, Neuherberg, 85764 Germany; 18 Icelandic Heart Association, Kopavogur, 201 Iceland; 19 Faculty of Medicine, University of Iceland, Reykjavik, 101 Iceland; 20 deCODE Genetics, Reykjavik, 101 Iceland; 21 Department of Internal Medicine, Institute of Medicine, Sahlgrenska Academy, University of Gothenburg, Gothenburg, SE-405 30 Sweden; 22 Department Epidemiology and Biostatistics, School of Public Health, Imperial College, London, SW7 2AZ UK; 23 Cardiology Department, Ealing Hospital NHS Trust, Middlesex, UB1 3HW UK; 24 Department of Medicine A, University of Greifswald, Greifswald, 17489 Germany; 25 Department of Clinical Sciences, Lund University, Malmö, 22362 Sweden; 26 Department of Orthopedics, Skåne University Hospital, Malmö, S-205 02 Sweden; 27 Phoenix Epidemiology and Clinical Research Branch, National Institute of Diabetes and Digestive and Kidney Diseases, NIH, Phoenix, AZ 85014 USA; 28 Wellcome Trust Sanger Institute, Wellcome Trust Genome Campus, Hinxton, CB10 1SA UK; 29 NIHR Cambridge Biomedical Research Centre, Institute of Metabolic Science, Addenbrooke’s Hospital, Cambridge, CB2 OQQ UK; 30 Institute of Metabolic Science, Addenbrooke’s Hospital, University of Cambridge Metabolic Research Laboratories, Cambridge, CB2 OQQ UK; 31 Rush Alzheimer’s Disease Center, Rush University Medical Center, Chicago, IL 60612 USA; 32 Lübeck Interdisciplinary Platform for Genome Analytics, Institutes of Neurogenetics and Experimental & Integrative Genomics, University of Lübeck, Lübeck, 23562 Germany; 33 School of Public Health, Faculty of Medicine, Imperial College London, London, W6 8RP UK; 34 Centre of Oral Health, Department of Prosthetic Dentistry, Gerodontology and Biomaterials, University of Greifswald, Greifswald, 17489 Germany; 35 Department of Biostatistics and Center for Statistical Genetics, University of Michigan, Ann Arbor, MI 48109 USA; 36 Division of Statistical Genomics, Department of Genetics, Washington University School of Medicine, St Louis, MO 63110 USA; 37 Division of Biostatistics, Washington University School of Medicine, St Louis, MO 63110 USA; 38 Department of Surgical Sciences, Uppsala University, Uppsala, 75185 Sweden; 39 Usher Institute of Population Health Sciences and Informatics, University of Edinburgh, Edinburgh, Scotland EH8 9AG UK; 40 Department of Epidemiology Graduate School of Public Health, University of Pittsburgh, Pittsburgh, PA 15261 USA; 41 California Pacific Medical Center Research Institute, San Francisco, CA 94107 USA; 42 Department of Medicine, University of Eastern Finland and Kuopio University Hospital, Kuopio, 70210 Finland; 43 Mel and Enid Zuckerman College of Public Health, University of Arizona, Tucson, AZ 85714 USA; 44 Department of Preventive Medicine, Ajou University School of Medicine, Youngtong-Gu, Suwon 16499 Korea; 45 Department of Internal Medicine, Seoul National University College of Medicine, Seoul, 03080 Korea; 46 Department of Internal Medicine, Chungbuk National University Hospital, Cheongju Si, Korea; 47 Computer Science and Artificial Intelligence Laboratory, MIT, Cambridge, MA 02139 USA; 48 Institute of Human Genetics, MRI, Technische Universität München, Munich, 81675 Germany; 49 Beth Israel Deaconess Medical Center, Boston, MA 02215 USA; 50 Medical Genomics and Metabolic Genetics Branch, National Human Genome Research Institute, Bethesda, MD 20892 USA; 51 Program in Translational NeuroPsychiatric Genomics, Department of Neurology, Brigham and Women’s Hospital, Boston, MA 02115 USA; 52 Program in Medical and Population Genetics, Broad Institute, Cambridge, MA 02142 USA; 53 Lipid Clinic at the Interdisciplinary Metabolism Center, Charité – Universitätsmedizin Berlin, corporate member of Freie Universität Berlin, Humboldt-Universität zu Berlin, and Berlin Institute of Health, Berlin, 13353 Germany; 54 Institute of Medical and Human Genetics, Charité – Universitätsmedizin Berlin, Berlin, 13353 Germany; 55 Department of Human Nutrition, Wageningen University, PO Box 17, Wageningen, 6700 AA The Netherlands; 56 Alan Edwards Centre for Research on Pain, McGill University, Montreal, H3A 0G1 Canada; 57 Regional Center for Neurosensory Disorders, School of Dentistry, University of North Carolina at Chapel Hill, Chapel Hill, NC 27599 USA; 58 Department of General Practice and Primary Health Care, University of Helsinki, Helsinki, 00014 Finland; 59 Unit of General Practice, Helsinki University Central Hospital, Helsinki, 00014 Finland; 60 Folkhalsan Research Centre, Helsinki, 00250 Finland; 61 Vasa Central Hospital, Vasa, 65130 Finland; 62 National Institute for Health and Welfare, Helsinki, 00271 Finland; 63 Laboratory of Epidemiology and Population Sciences, Intramural Research Program, National Institute for Aging, Bethesda, MD 20892 USA; 64 Research Unit of Molecular Epidemiology, Helmholtz Zentrum München - German Research Center for Environmental Health, Neuherberg, 85764 Germany; 65 Institute of Genetic Epidemiology, Helmholtz Zentrum München - German Research Center for Environmental Health, Neuherberg, 85764 Germany; 66 Institute for Integrative Genome Biology, University of California, Riverside, CA 92521 USA; 67 Department of Botany and Plant Sciences, University of California, Riverside, CA 92521 USA; 68 Departments of Medicine and Epidemiology, Boston University School of Medicine and Public Health, Boston, MA 02118 USA; 69 German Center for Diabetes Research (DZD), Neuherberg, Germany; 70 CCG Type 2 Diabetes, Helmholtz Zentrum München, Neuherberg, 85764 Germany; 71 CCG Nutrigenomics and Type 2 Diabetes. Helmholtz Zentrum München, Neuherberg, 85764 Germany; 72 National Heart and Lung Institute, Imperial College London, London, SW3 6LY UK; 73 Center for Genome Science, National Institute of Health, Osong Health Technology Administration Complex, Chungcheongbuk-do, 28159 Korea; 74 MRC Human Genetics Unit, IGMM, University of Edinburgh, Edinburgh, Scotland EH4 2XU UK; 75 Department of Pharmaceutical Sciences, SUNY Binghamton, Binghamton, NY 13902 USA; 76 Interfaculty Institute for Genetics and Functional Genomics, University of Greifswald, Greifswald, 17487 Germany; 77 Department of Exercise and Nutrition Sciences, George Washington University, Washington, DC 20052 USA; 78 Research Center for Genetic Medicine, Children’s National Medical Center, Washington, DC 20052 USA; 79 Division of Biostatistics, School of Public Health, University of California, Berkeley, CA 94720 USA; 80 Division of Rheumatology, Department of Medicine, Duke Molecular Physiology Institute, Duke University School of Medicine, Durham, NC 27710 USA; 81 Endocrinology and Internal Medicine, Aarhus University Hospital, Aarhus, DK 8000 Denmark; 82 Department of Human Genetics, Hannover Medical School, Hannover, 30625 Germany; 83 Hannover Unified Biobank, Hannover Medical School, Hannover, 30625 Germany; 84 Department of Medical Sciences, Uppsala University, Uppsala, 75185 Sweden; 85 Division of Cardiovascular Medicine, Department of Medicine, Stanford University School of Medicine, Stanford, CA 94305 USA; 86 Institute for Community Medicine, University of Greifswald, Greifswald, 17489 Germany; 87 Department of Physiology, Institute of Neuroscience and Physiology, Sahlgrenska Academy, University of Gothenburg, Gothenburg, SE 405 30 Sweden; 88 Thurston Arthritis Research Center, University of North Carolina at Chapel Hill, Chapel Hill, NC 27517 USA; 89 Department of Clinical Sciences and Orthopaedics, Lund University, Skåne University Hospital SUS, Malmö, 22362 Sweden; 90 Department of Public Health and Primary Care, University of Cambridge, Cambridge, CB1 8RN UK; 91 The Novo Nordisk Foundation Center for Basic Metabolic Research, Section of Metabolic Genetics, University of Copenhagen, Copenhagen, 2100 Denmark; 92 Department of Environmental Medicine and Public Health, Icahn School of Medicine at Mount Sinai, New York, NY 10029 USA; 93 Department of Medicine, University of Helsinki and Helsinki University Central Hospital, Helsinki, 00029 Finland; 94 Endocrinology, Abdominal Center, University of Helsinki and Helsinki University Central Hospital, Helsinki, 00029 Finland; 95 Department of Health, National Institute for Health and Welfare, Helsinki, 00271 Finland; 96 Minerva Foundation Institute for Medical Research, Helsinki, 00290 Finland; 97 Division of Cardiology, Department of Medicine, Duke Molecular Physiology Institute, Duke University School of Medicine, Durham, NC 27710 USA; 98 Sticht Center on Aging, Wake Forest School of Medicine, Winston-Salem, NC 27157 USA; 99 Institute of Behavioural Sciences, University of Helsinki, Helsinki, FI00014 Finland; 100 University of California San Francisco, San Francisco, CA 94143 USA; 101 School of Medicine and Pharmacology, University of Western Australia, Perth, 6009 Australia; 102 Centre for Kidney Research, School of Public Health, University of Sydney, Sydney, 2006 Australia; 103 Wellcome Trust Centre for Human Genetics, Oxford University, Oxford, OX3 7BN UK; 104 Department of Epidemiology and Prevention, Wake Forest School of Medicine, Winston-Salem, NC 27517 USA; 105 Max Planck Institute for Molecular Genetics, Berlin, 14195 Germany; 106 Max Planck Institute for Human Development, Berlin, 14195 Germany; 107 Institute of Human Genetics, Helmholtz Zentrum München - German Research Center for Environmental Health, Neuherberg, 85764 Germany; 108 Buck Institute for Research on Aging, Novato, CA 94945 USA; 109 Leonard Davis School of Gerontology, University of Southern California, LA, CA 90089 USA; 110 Geriatrics Research and Education Clinical Center, Baltimore Veterans Administration Medical Center, Baltimore, MD 21201 USA; 111 Institute of Translational Medicine, University of Liverpool, Liverpool, L69 3BX UK; 112 Center for Aging and Population Health, University of Pittsburgh, Pittsburgh, PA 15261 USA; 113 Oregon Health & Science University, Portland, OR 97239 USA; 114 Department of Clinical Genetics, Erasmus MC, Rotterdam, 300 CA The Netherlands; 115 Centre for Medical Systems Biology and Netherlands Consortium on Healthy Aging, Leiden, RC2300 The Netherlands; 116 Institute for Molecular Medicine Finland (FIMM), University of Helsinki, Helsinki, 00251 Finland; 117 Department of Medical Genetics, University of Helsinki and University Central Hospital, Helsinki, FI00014 Finland; 118 Human Genetics and Computational Medicine and Bioinformatics, University of Michigan, Ann Arbor, MI 48109 USA; 119 Department of Medicine, Indiana University School of Medicine, Indianapolis, IN 46202 USA; 120 Diabetes and Obesity Research Program, University of Helsinki, Helsinki, FI00014 Finland; 121 Estonian Genome Center, University of Tartu, Tartu, Estonia; 122 Faculty of Medicine, Department of Public Health, University of Split, Split, 21000 Croatia; 123 Department of Endocrinology and Diabetes, Sir Charles Gardiner Hospital, Perth, 6009 Australia; 124 Molecular Medicine Centre, MRC Institute of Genetics and Molecular Medicine, Western General Hospital, Edinburgh, Scotland EH4 2XU UK; 125 Hjelt Institute, University of Helsinki, Helsinki, Finland; 126 Wellcome Trust Sanger Institute, Wellcome Trust Genome Campus, Hinxton, CB10 1SA UK; 127 Department of Medicine, University of California at Davis, Sacramento, CA 95817 USA; 128 Institute for Translational Genomic and Population Sciences, Los Angeles Biomedical Research Institute and Department of Pediatrics, Harbor UCLA Medical Center, Torrance, CA 90502 USA; 129 Department of Preventive Medicine, University of Tennessee Health Science Center, Memphis, TN 38163 USA; 130 Department of Genetics and Genomic Science, Institute of Genomics and Multiscale Biology, Icahn School of Medicine at Mount Sinai, New York, NY 10029 USA; 131 Department of Endocrinology and Metabolism, Landspitali, The National University Hospital of Iceland, Reykjavik, 101 Iceland; 132 Center for Translational Pain Medicine, Department of Anesthiology, Duke University Medical Center, Durham, NC 27110 USA; 133 Geriatric Research and Education Clinical Center (GRECC) - Veterans Administration Medical Center, Baltimore, MD 21201 USA; 134 Department of Epidemiology and Biostatistics, and the EMGO Institute, VU University Medical Center, Amsterdam, BT1081 The Netherlands; 135 Department of Medicine, McMaster University Medical Center, Hamilton, ON Canada L8N 3Z5; 136 Department of Pathology, Stony Brook School of Medicine, Stony Brook, NY 11794 USA; 137 Department of Neuroscience and Preventive Medicine, Danube-University Krems, Krems, 3500 Austria; 138 Diabetes Research Group, King Abdulaziz University, Jeddah, 12589 Saudi Arabia; 139 Dasman Diabetes Institute, Dasman, 15462 Kuwait; 140 Department of Medicine and Internal Medicine, Centre Hospitalier Universitaire Vaudois (CHUV), Lausanne, CH-1011 Switzerland; 141 Department of Epidemiology and Environmental Health, University at Buffalo, State University of New York, Buffalo, NY 14214 USA; 142 Institute of Cellular Medicine, Newcastle University, Newcastle upon Tyne, NE2 4HH UK; 143 Genetics of Complex Traits, University of Exeter Medical School, Exeter, EX1 2LU UK; 144 Institute of Medical Informatics, Biometry and Epidemiology, Chair of Epidemiology, Ludwig-Maximilians-Universität, Munich, 81377 Germany; 145 Institute of Medical Statistics and Epidemiology, Technical University, Munich, 81675 Germany; 146 Department of Epidemiology, University of Alabama at Birmingham, Birmingham, AL 35294 USA; 147 NIHR Cardiovascular Biomedical Research Unit, Royal Brompton and Harefield NHS Foundation Trust and Imperial College, London, SW3 6NP UK; 148 Imperial College Healthcare NHS Trust, London, W2 1NY UK; 149 Institute of Epidemiology I, Helmholtz Zentrum München - German Research Center for Environmental Health, Neuherberg, 85764 Germany; 150 Department of Medicine and Department of Medical and Molecular Genetics, Indiana University School of Medicine, Indianapolis, IN 46202 USA; 151 Departments of Medicine, Epidemiology, and Health Services, Cardiovascular Health Research Unit, University of Washington, Seattle, WA 98101 USA; 152 Kaiser Permanente Washington Health Research Institute, Washington, Seattle, WA 98101 USA; 153 Medical Genetics, GlaxoSmithKline, Philadelphia, PA 19112 USA; 154 The Charles Bronfman Institute for Personalized Medicine, Icahn School of Medicine at Mount Sinai, New York, NY 10029 USA; 155 Institute of Child Health and Development, Icahn School of Medicine at Mount Sinai, New York, NY 10029 USA; 156 The Genetics of Obesity and Related Traits Program, Icahn School of Medicine at Mount Sinai, New York, NY 10029 USA; 157 Department of Preventive Medicine, Icahn School of Medicine at Mount Sinai, New York, NY 10029 USA; 158 Faculty of Medicine in the Galilee, Bar-Ilan University, Safed, 1311502 Israel

*Nature Communications* **8**:80 doi:10.1038/s41467-017-00031-7; Article published online 19 Jul 2017

The previously published version of this Article contained typographical errors in the spelling of the author names Stephen C. J. Parker and H.-Erich Wichmann, which were incorrectly given as Stephan Parker and H.-Erich Wichman, respectively. Furthermore, Affiliation 53 was incorrectly given as ‘Research Group on Geriatrics, The Berlin Aging Study II, Charité–Universitätsmedizin Berlin, Berlin 13353, Germany’, Affiliation 66 was incorrectly given as ‘Department of Botany & Plant Sciences, Institute for Integrative Genome Biology, University of California, Riverside, CA 92521, USA’, and Affiliation 152 was incorrectly given as ‘Group Health Research Institute, Group Health Cooperative, Seattle, WA 98101, USA’. These errors have been corrected in the PDF and HTML versions of the Article.

