# Supplementary material for: Large meta-analysis of genome-wide association studies identifies five loci for lean body mass
Source: Nat Commun. 2017 Jul 19;8:80. doi: 10.1038/s41467-017-00031-7 (PMC5517526; doi:10.1038/s41467-017-00031-7)
Supplement: Supplementary file 1 — Supplementary Information [file 41467_2017_31_MOESM1_ESM.pdf]

File Name: Supplementary Information
Description: Supplementary Figures, Supplementary Tables, Supplementary Notes and Supplementary References.

Supplementary Figures

Supplementary Fig. 1: Overview of study design. The Stage II Replication included both in-silico replication as well as de-novo genotyping, as some cohorts with existing GWAS data did not have data available at the time of the initial discovery efforts. Analyses involving cohorts of European ancestry were the pre-specified primary focus of the project.

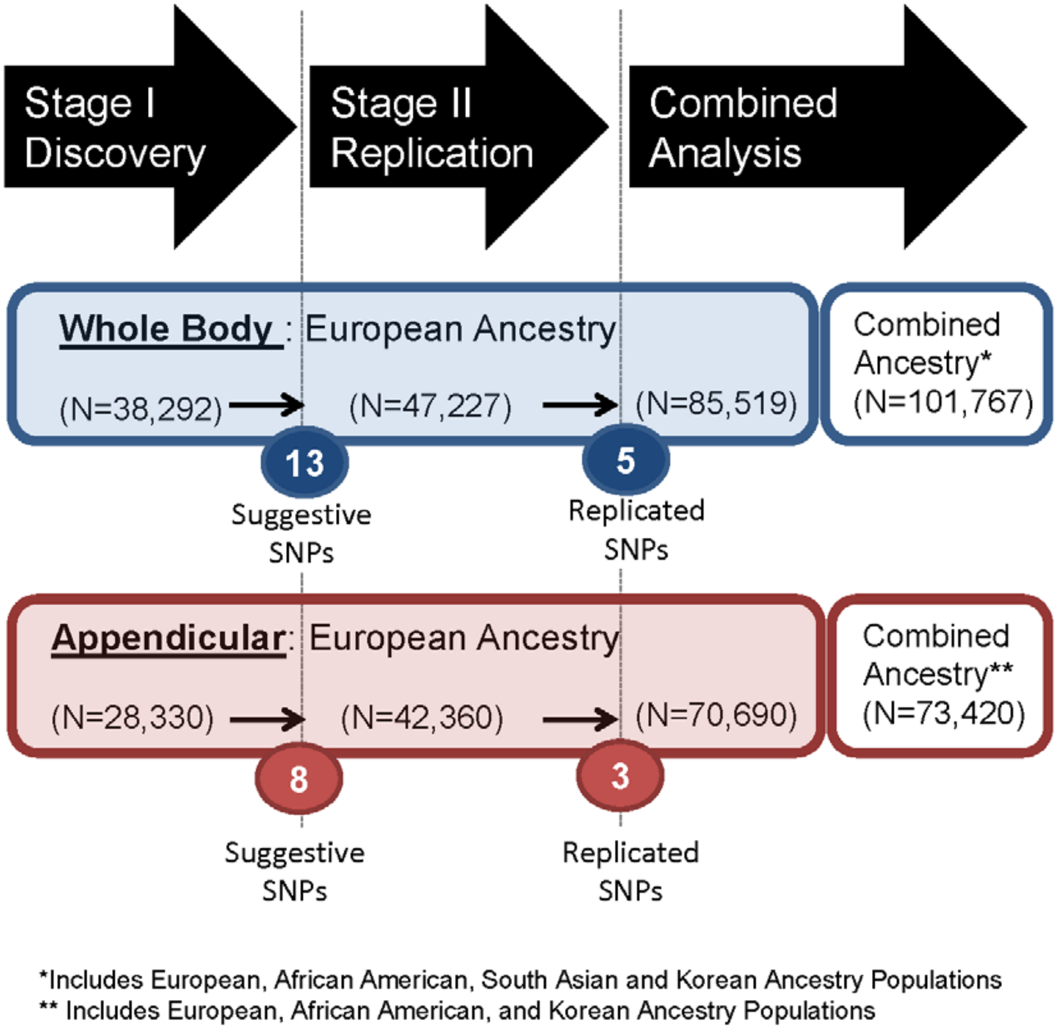

Supplementary Fig. 2: Quantile – quantile plots for whole body lean mass and appendicular lean mass. The plots compare observed p-values (in -log 10 scale) from additive model statistics to those expected under the null distribution using fixed-effects for all analyzed HapMap CEU imputed SNPs passing quality control criteria in the studies

Whole body lean mass

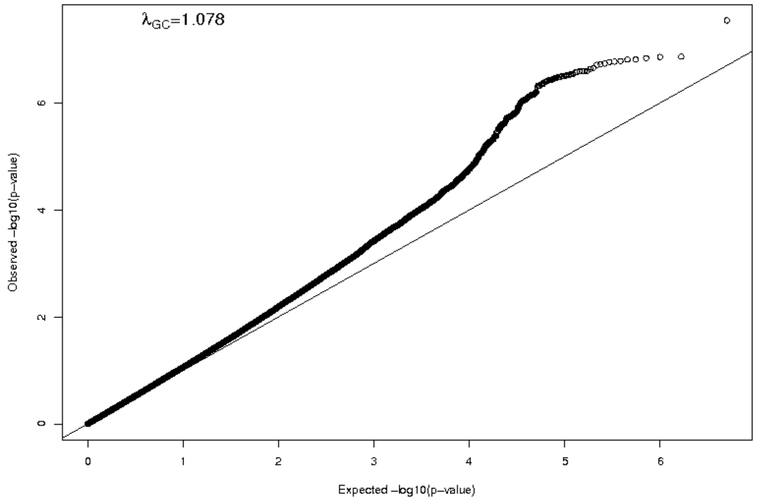

Appendicular lean mass

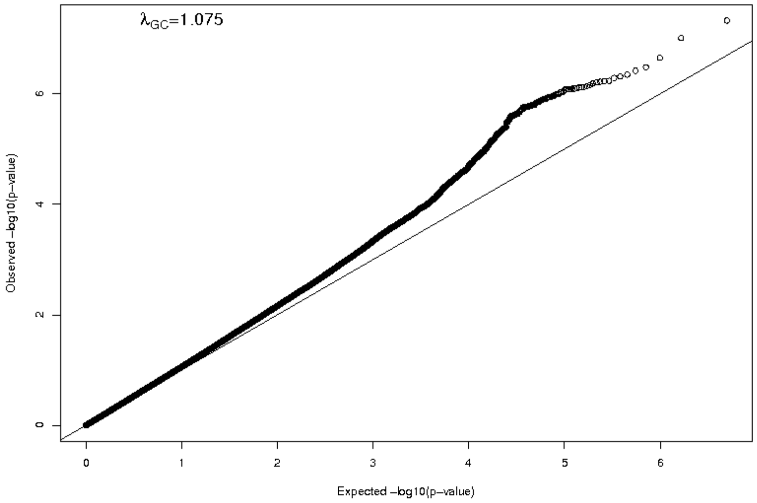

Supplementary Fig. 3: Manhattan plots. Plots display–Log10 (*p*-value) for discovery meta-analysis of genome-wide associations with (a) whole body lean mass (b) and appendicular lean mass for all SNPs analyzed using fixed-effects.

3a.

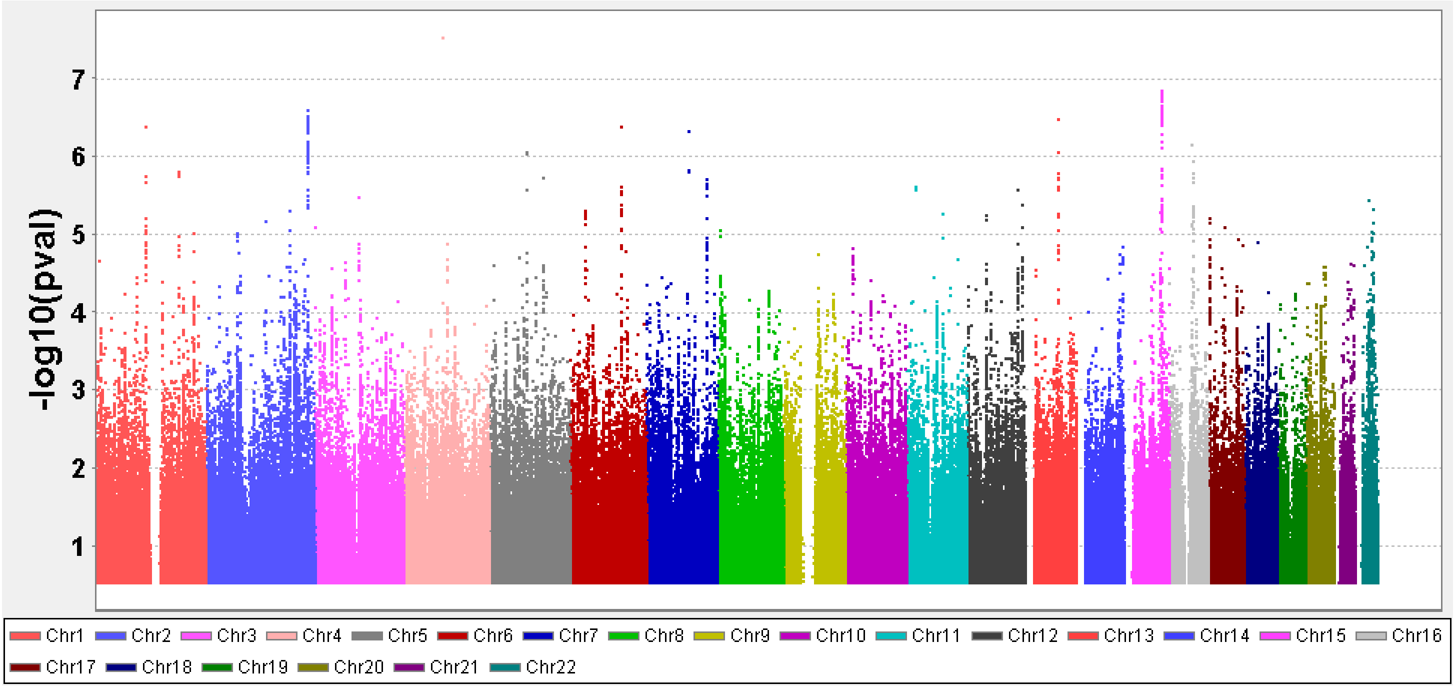

3b.

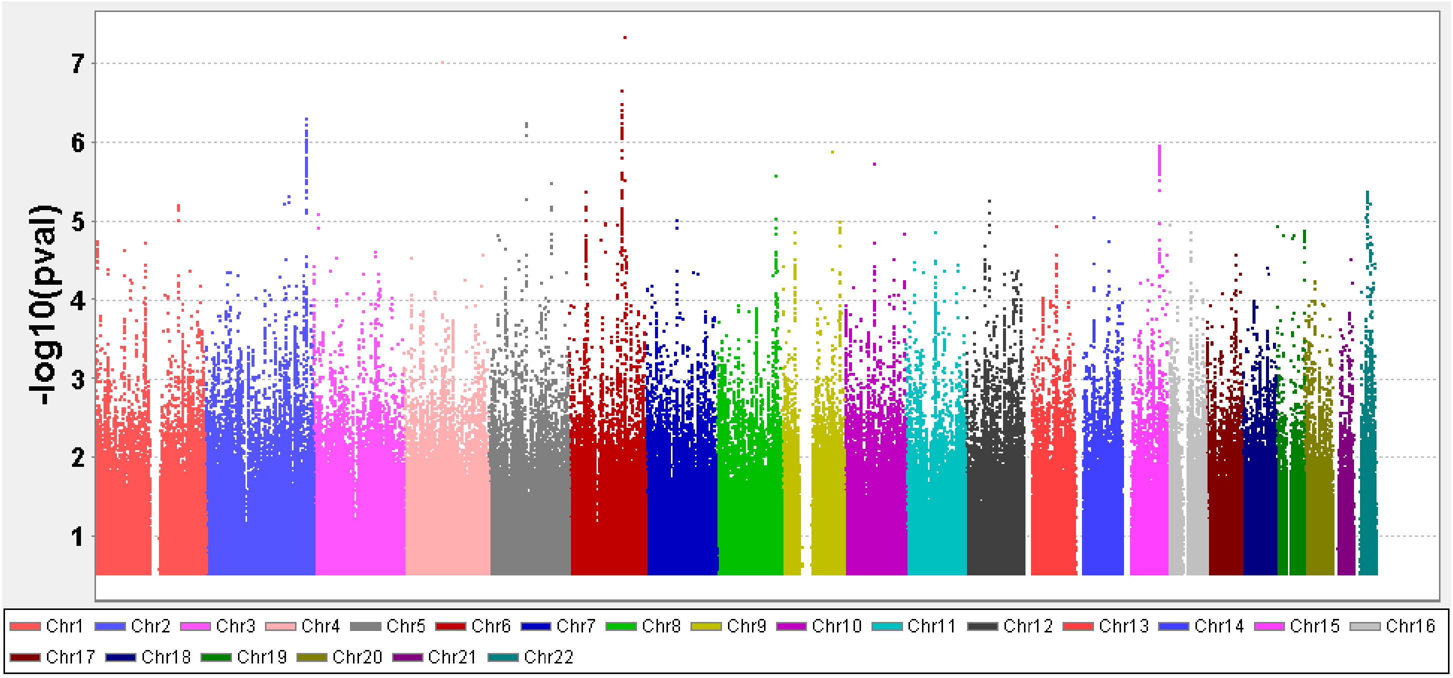

Supplementary Fig. 4: Forest plots of effect sizes across all discovery and replication cohorts for the five replicated genome-wide significant SNPs for whole body lean mass and the three replicated genome-wide significant SNPs for appendicular lean mass.

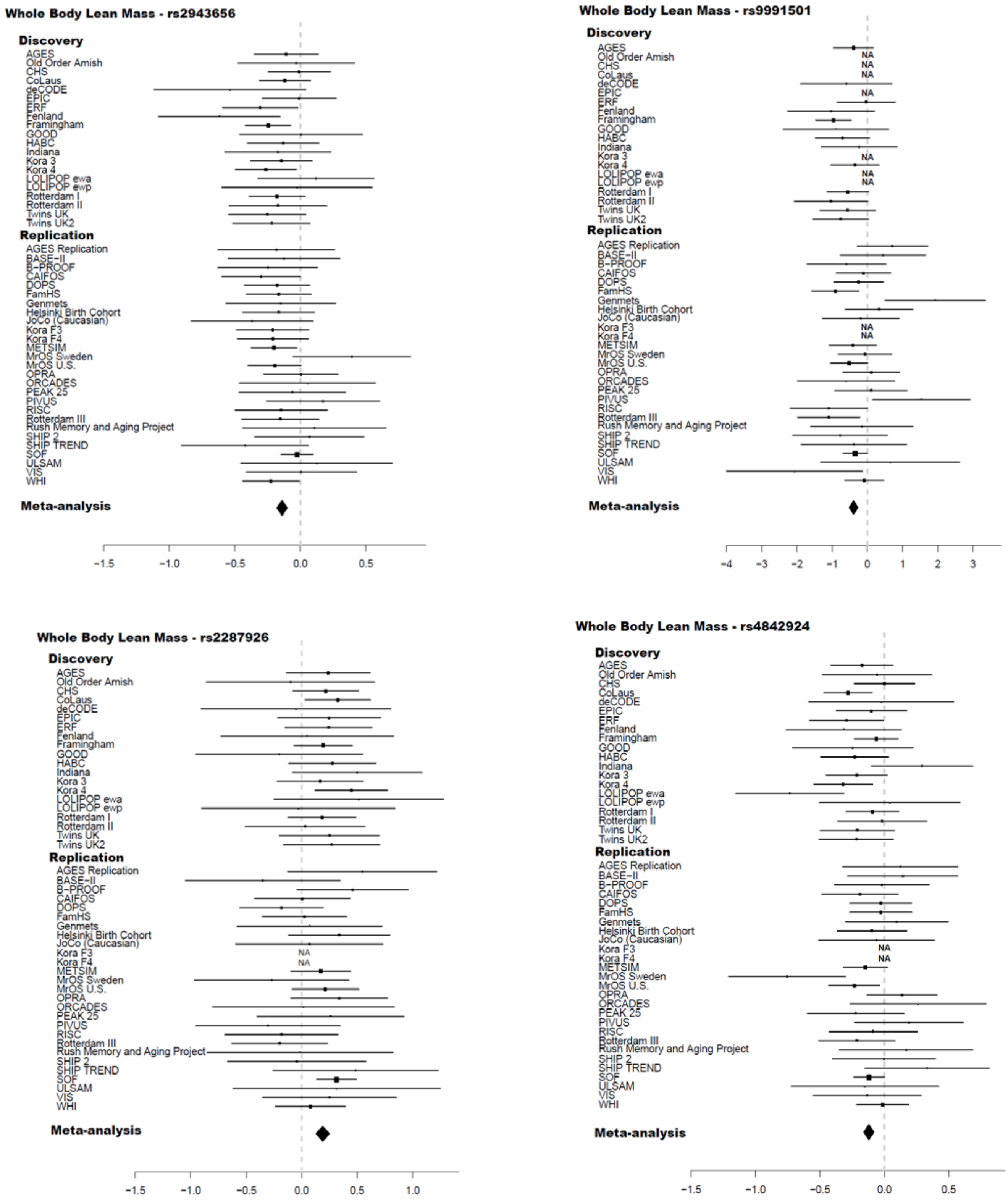

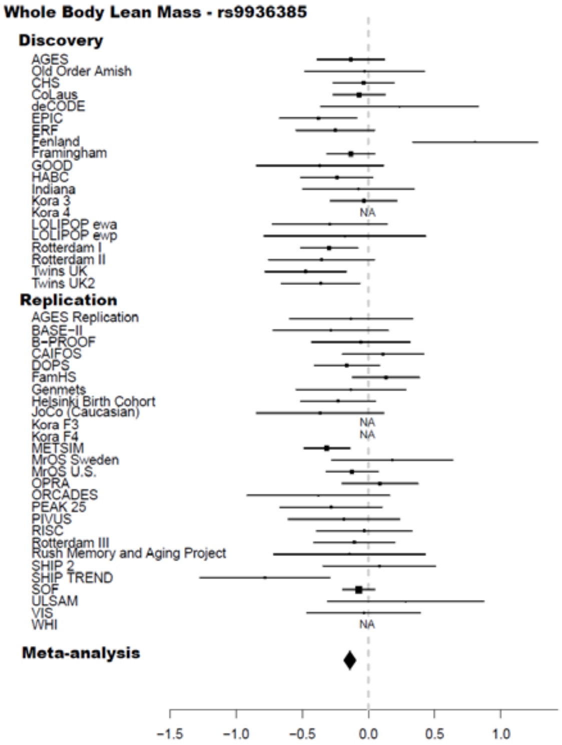
Whole Body Lean Mass - rs9936385
Discovery
AGES
Old Order Amish
CHS
CoLaus
deCODE
EPIC
ERF
Fenland
Framingham
GOOD
HABC
Indiana
Kora 3
Kora 4
NA
LOLIPOP ewa
LOLIPOP ewp
Rotterdam I
Rotterdam II
Twins UK
Twins UK2
Replication
AGES Replication
BASE-II
B-PROOF
CAIFOS
DOPS
FamHS
Genmets
Helsinki Birth Cohort
JoCo (Caucasian)
Kora F3
NA
Kora F4
NA
METSIM
MrOS Sweden
MrOS U.S.
OPRA
ORCADES
PEAK 25
PIVUS
RISC
Rotterdam III
Rush Memory and Aging Project
SHIP 2
SHIP TREND
SOF
ULSAM
VIS
WHI
NA
Meta-analysis
-1.5
-1.0
-0.5
0.0
0.5
1.0

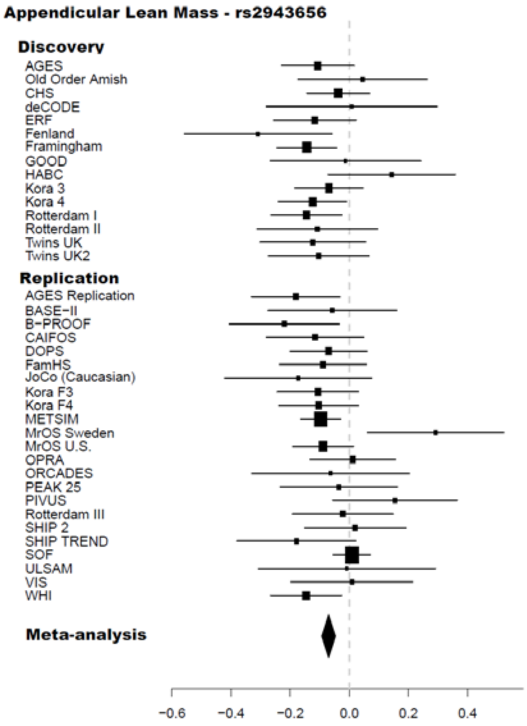
Appendicular Lean Mass - rs2943656
Discovery
AGES
Old Order Amish
CHS
deCODE
ERF
Fenland
Framingham
GOOD
HABC
Kora 3
Kora 4
Rotterdam I
Rotterdam II
Twins UK
Twins UK2
Replication
AGES Replication
BASE-II
B-PROOF
CAIFOS
DOPS
FamHS
JoCo (Caucasian)
Kora F3
Kora F4
METSIM
MrOS Sweden
MrOS U.S.
OPRA
ORCADES
PEAK 25
PIVUS
Rotterdam III
SHIP 2
SHIP TREND
SOF
ULSAM
VIS
WHI
Meta-analysis
-0.6
-0.4
-0.2
0.0
0.2
0.4

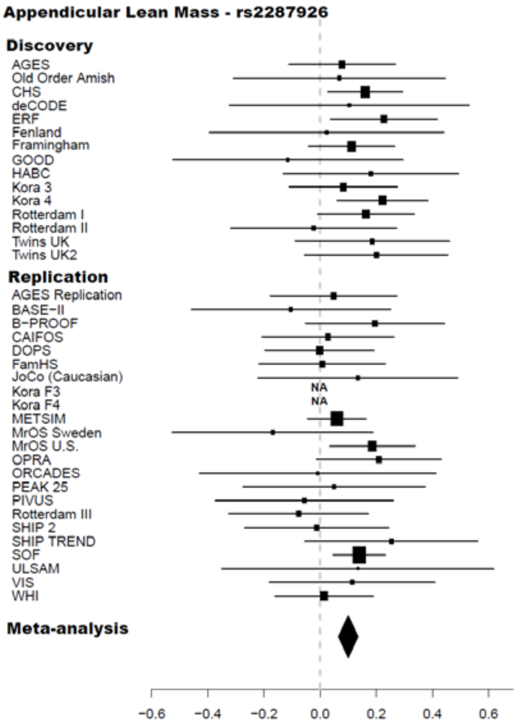
Appendicular Lean Mass - rs2287926
Discovery
AGES
Old Order Amish
CHS
deCODE
ERF
Fenland
Framingham
GOOD
HABC
Kora 3
Kora 4
Rotterdam I
Rotterdam II
Twins UK
Twins UK2
Replication
AGES Replication
BASE-II
B-PROOF
CAIFOS
DOPS
FamHS
JoCo (Caucasian)
Kora F3
NA
Kora F4
NA
METSIM
MrOS Sweden
MrOS U.S.
OPRA
ORCADES
PEAK 25
PIVUS
Rotterdam III
SHIP 2
SHIP TREND
SOF
ULSAM
VIS
WHI
Meta-analysis
-0.6
-0.4
-0.2
0.0
0.2
0.4
0.6

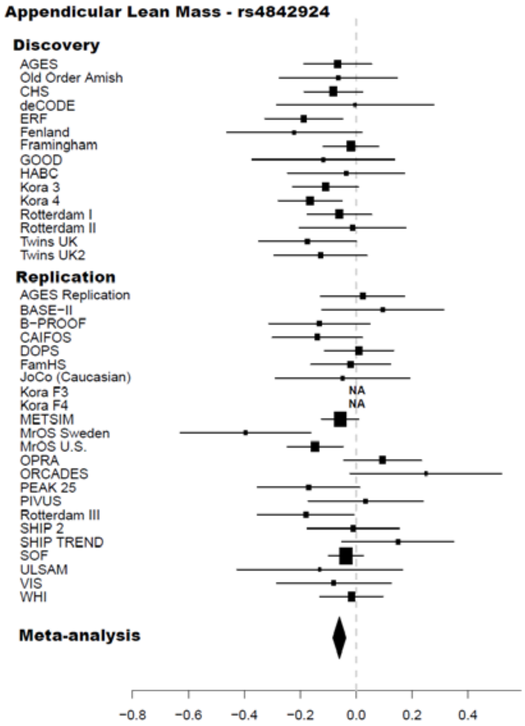
Appendicular Lean Mass - rs4842924
Discovery
AGES
Old Order Amish
CHS
deCODE
ERF
Fenland
Framingham
GOOD
HABC
Kora 3
Kora 4
Rotterdam I
Rotterdam II
Twins UK
Twins UK2
Replication
AGES Replication
BASE-II
B-PROOF
CAIFOS
DOPS
FamHS
JoCo (Caucasian)
Kora F3
NA
Kora F4
NA
METSIM
MrOS Sweden
MrOS U.S.
OPRA
ORCADES
PEAK 25
PIVUS
Rotterdam III
SHIP 2
SHIP TREND
SOF
ULSAM
VIS
WHI
Meta-analysis
-0.8
-0.6
-0.4
-0.2
0.0
0.2
0.4

Supplementary Fig. 5: Locus zoom plots of the genome wide significant SNPs for whole body and appendicular lean mass. The p-values (in –Log10 scale) for the lead SNP is based on the combined discovery and replication samples, whereas the p-values for the other SNPs are based on discovery samples only

rs2943656 - Whole body

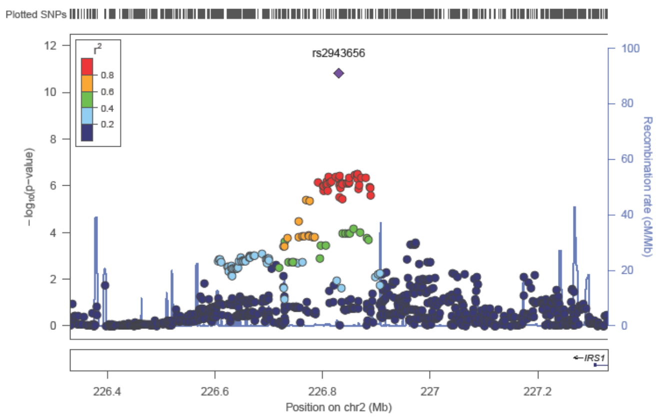

rs9991501 - Whole body

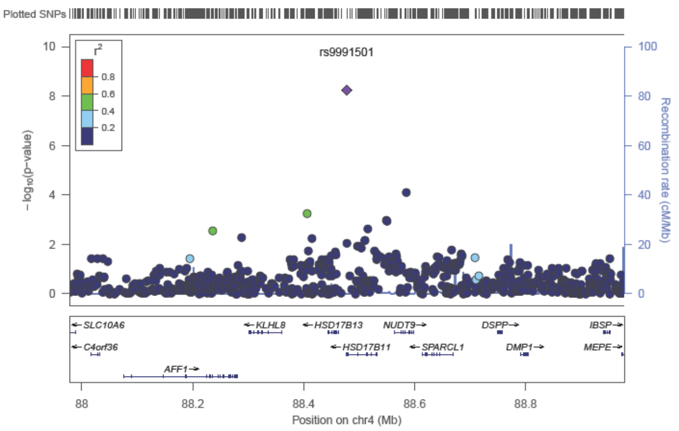

rs9936385 - Whole body

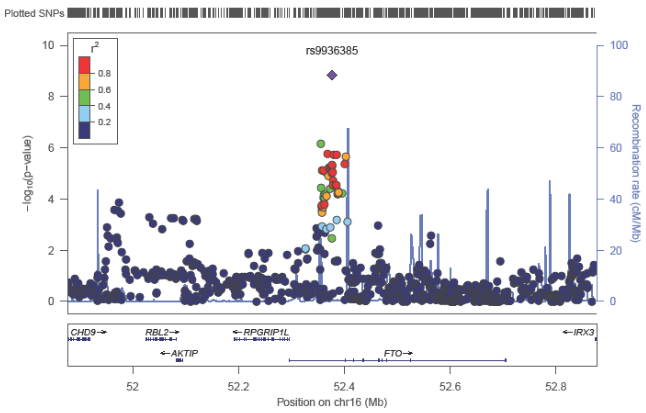

rs2287926 - Whole body

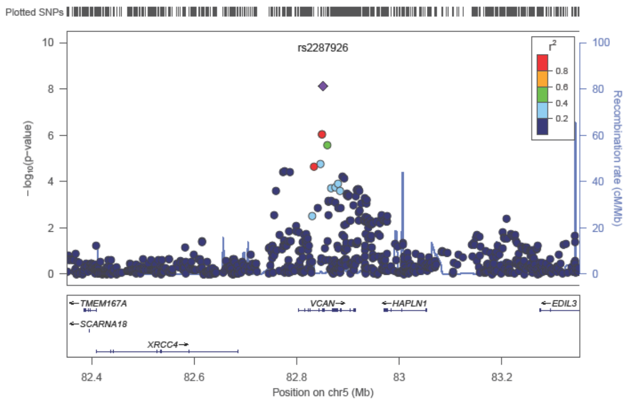

rs4842924 - Whole body

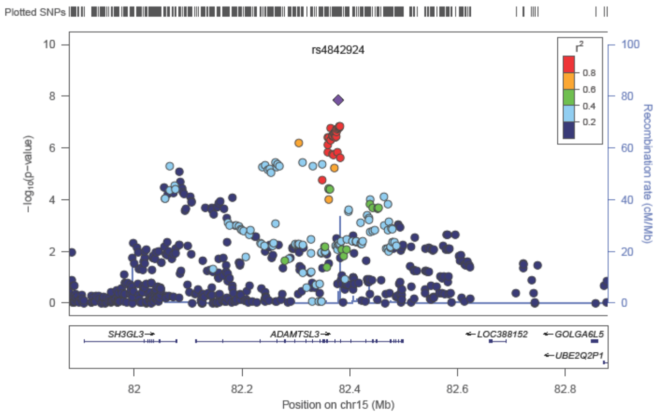

rs2943656 - Appendicular

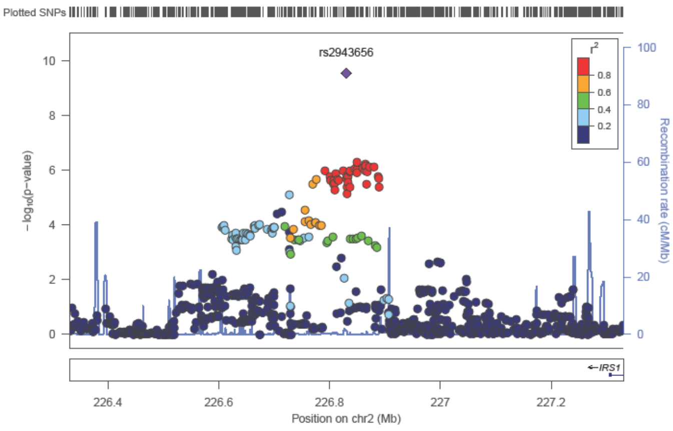

rs2287926 - Appendicular

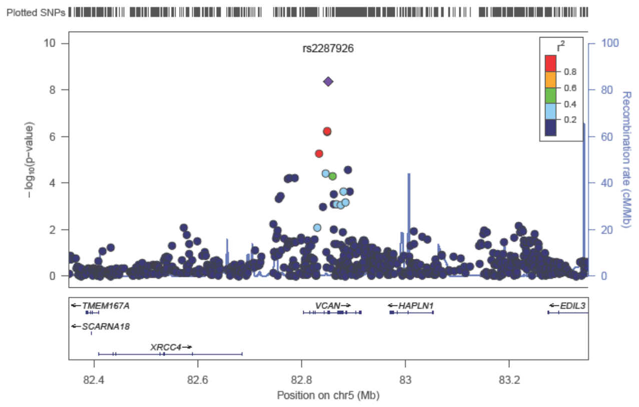

rs4842924 - Appendicular

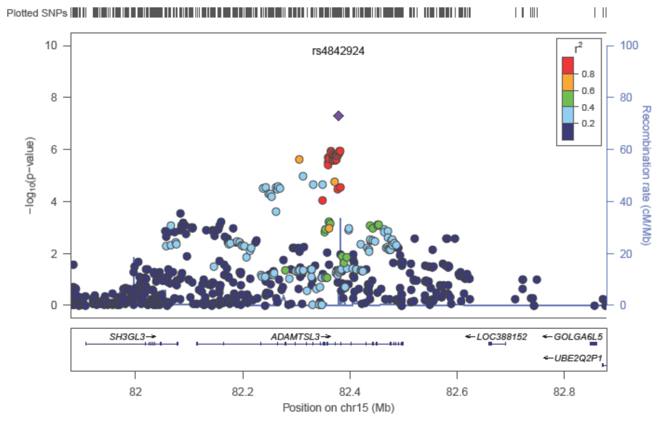

Figures S6-S13: Regional plots with functional annotations of chromatin states identified by experiments of ENCODE Project and Epigenetic Roadmap Project. Colors in chromatin state plots indicate different regulatory functions listed in the legend table.

DESCRIPTION
Active TSS
Promoter Upstream TSS
Promoter Downstream TSS 1
Promoter Downstream TSS 2
Transcribed - 5' preferential
Strong transcription
Transcribed - 3' preferential
Weak transcription
Transcribed & regulatory (Prom/Enh)
Transcribed 5' preferential and Enh
Transcribed 3' preferential and Enh
Transcribed and Weak Enhancer
Active Enhancer 1
Active Enhancer 2
Active Enhancer Flank
Weak Enhancer 1
Weak Enhancer 2
Primary H3K27ac possible Enhancer
Primary DNase
ZNF genes & repeats
Heterochromatin
Poised Promoter
Bivalent Promoter
Repressed Polycomb
Quiescent/Low

Supplementary Fig. 6: Regional plot of rs2943656 associated with appendicular lean mass and chromatin states in muscle, smooth muscle (sm.), fat, brain, blood, and gastrointestinal tract tissues

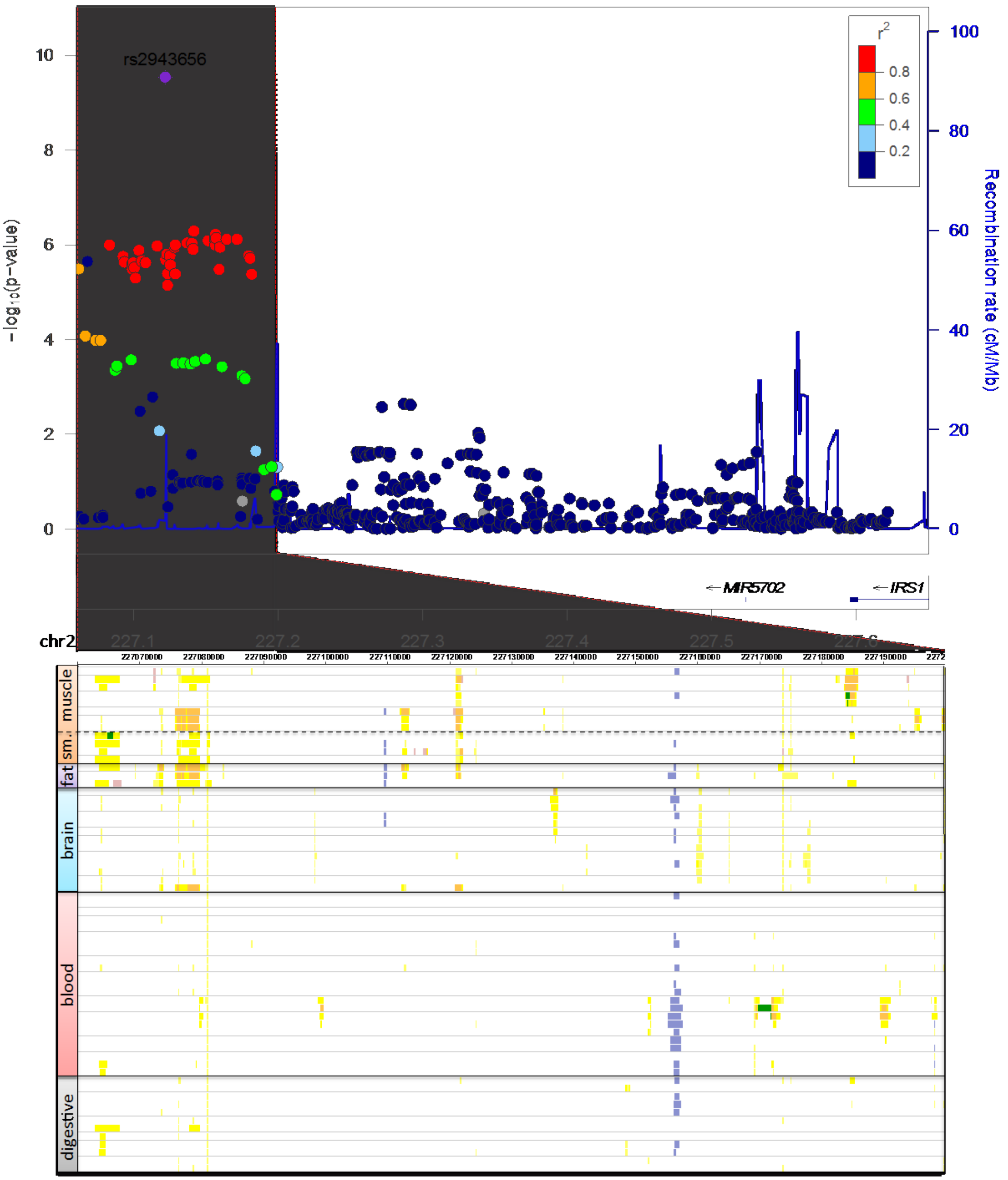

Supplementary Fig. 7: Regional plot of rs2943656 associated with whole-body lean mass and chromatin states in muscle, smooth muscle (sm.), fat, brain, blood, and gastrointestinal tract tissues

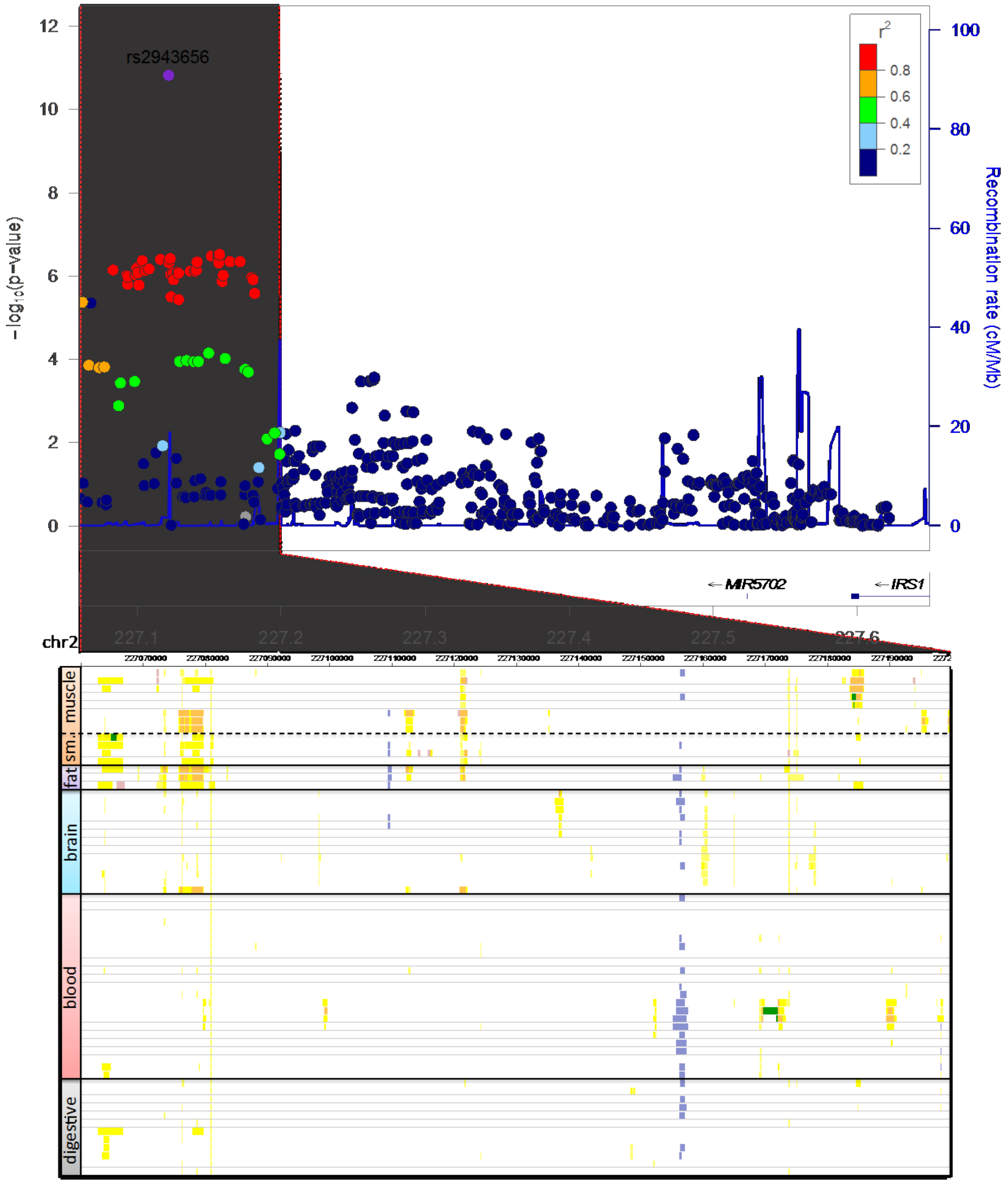

Supplementary Fig. 8: Regional plot of rs9991501 associated with whole-body lean mass and chromatin states in muscle, smooth muscle (sm.), fat, brain, blood, and gastrointestinal tract tissues

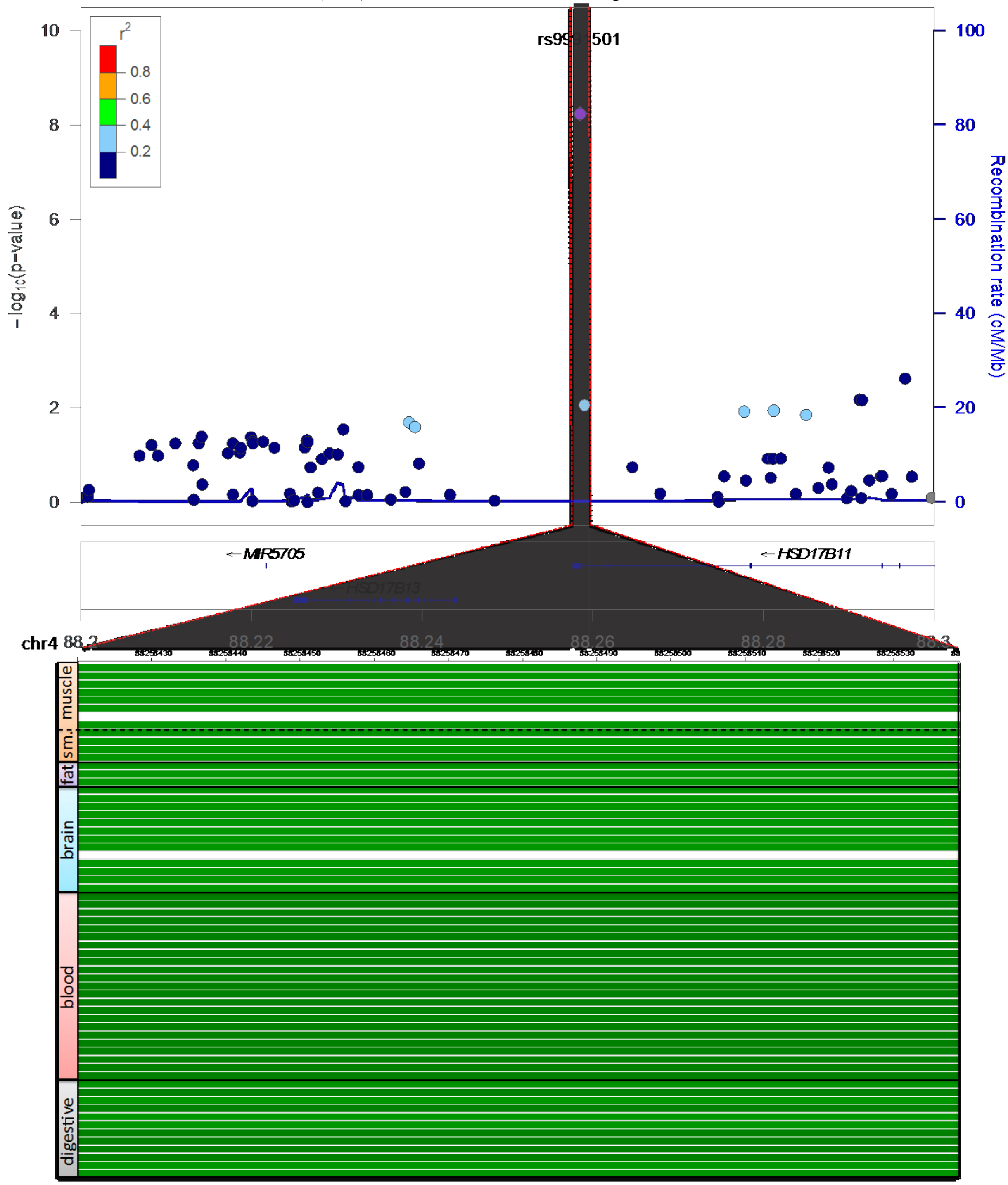

Supplementary Fig. 9: Regional plot of rs2287926 associated with appendicular lean mass and chromatin states in muscle, smooth muscle (sm.), fat, brain, blood, and gastrointestinal tract tissues

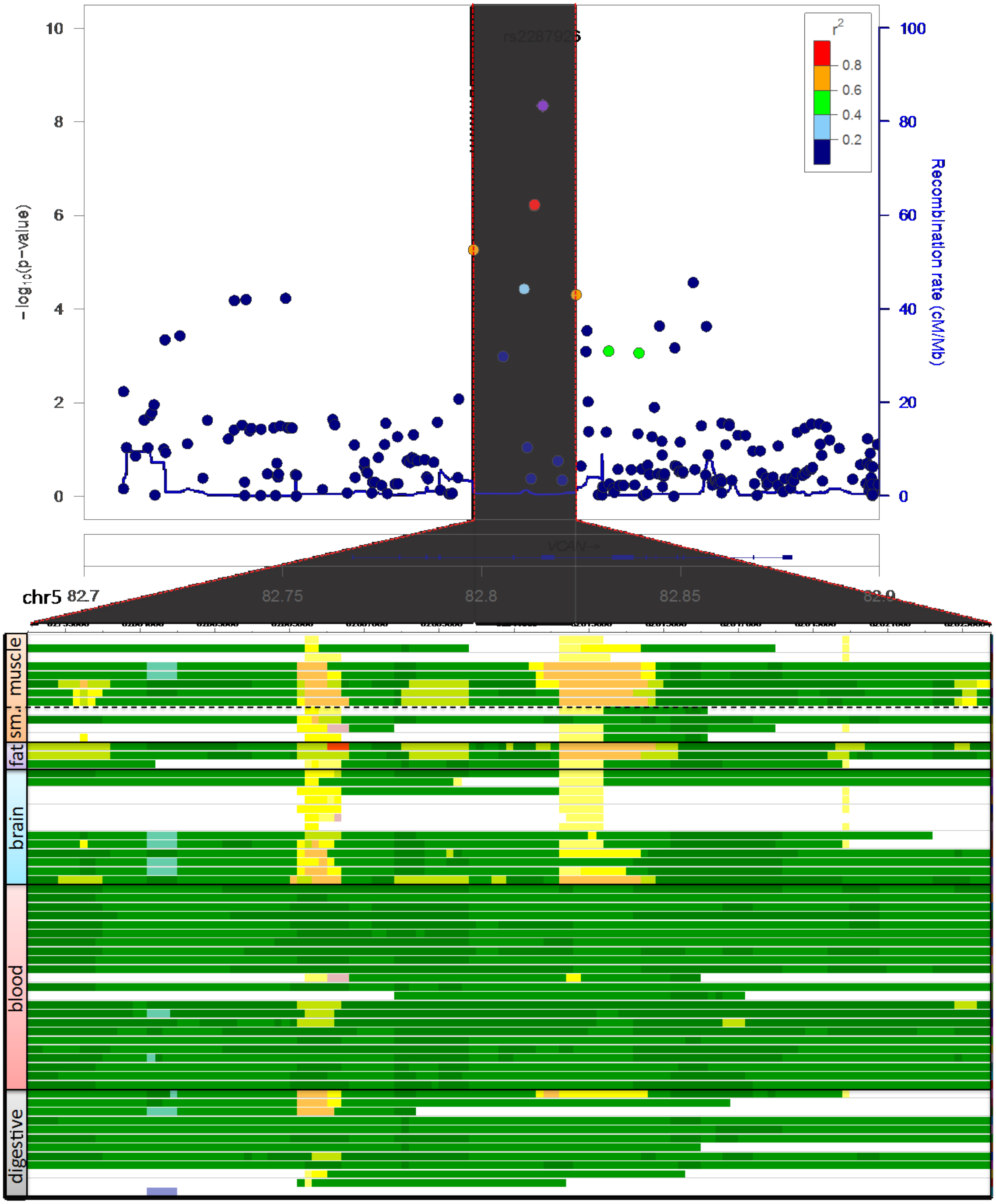

Supplementary Fig. 10: Regional plot of rs2287926 associated with whole-body lean mass and chromatin states in muscle, smooth muscle (sm.), fat, brain, blood, and gastrointestinal tract tissues

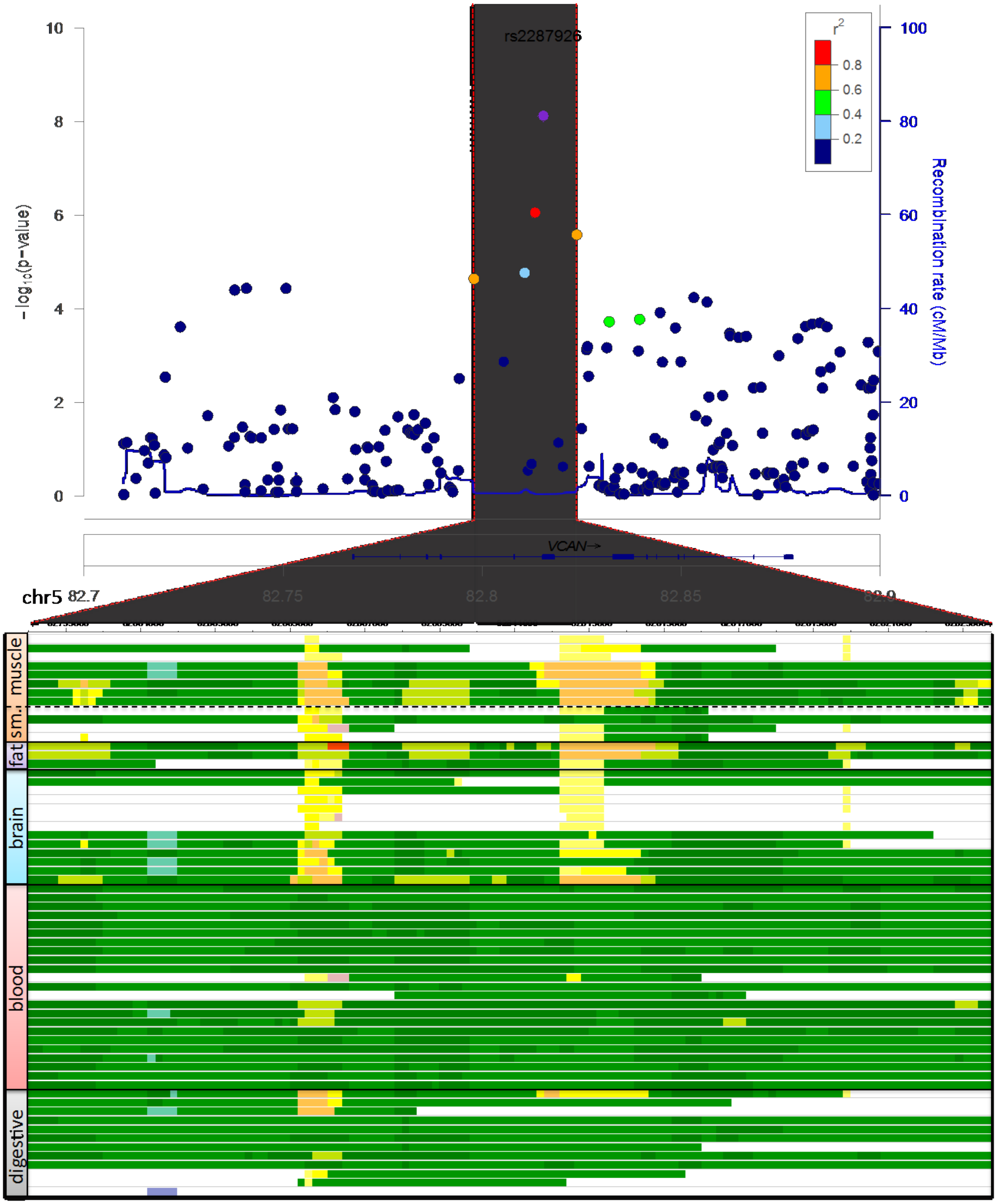

Supplementary Fig. 11: Regional plot of rs4842924 associated with appendicular lean mass and chromatin states in muscle, smooth muscle (sm.), fat, brain, blood, and gastrointestinal tract tissues

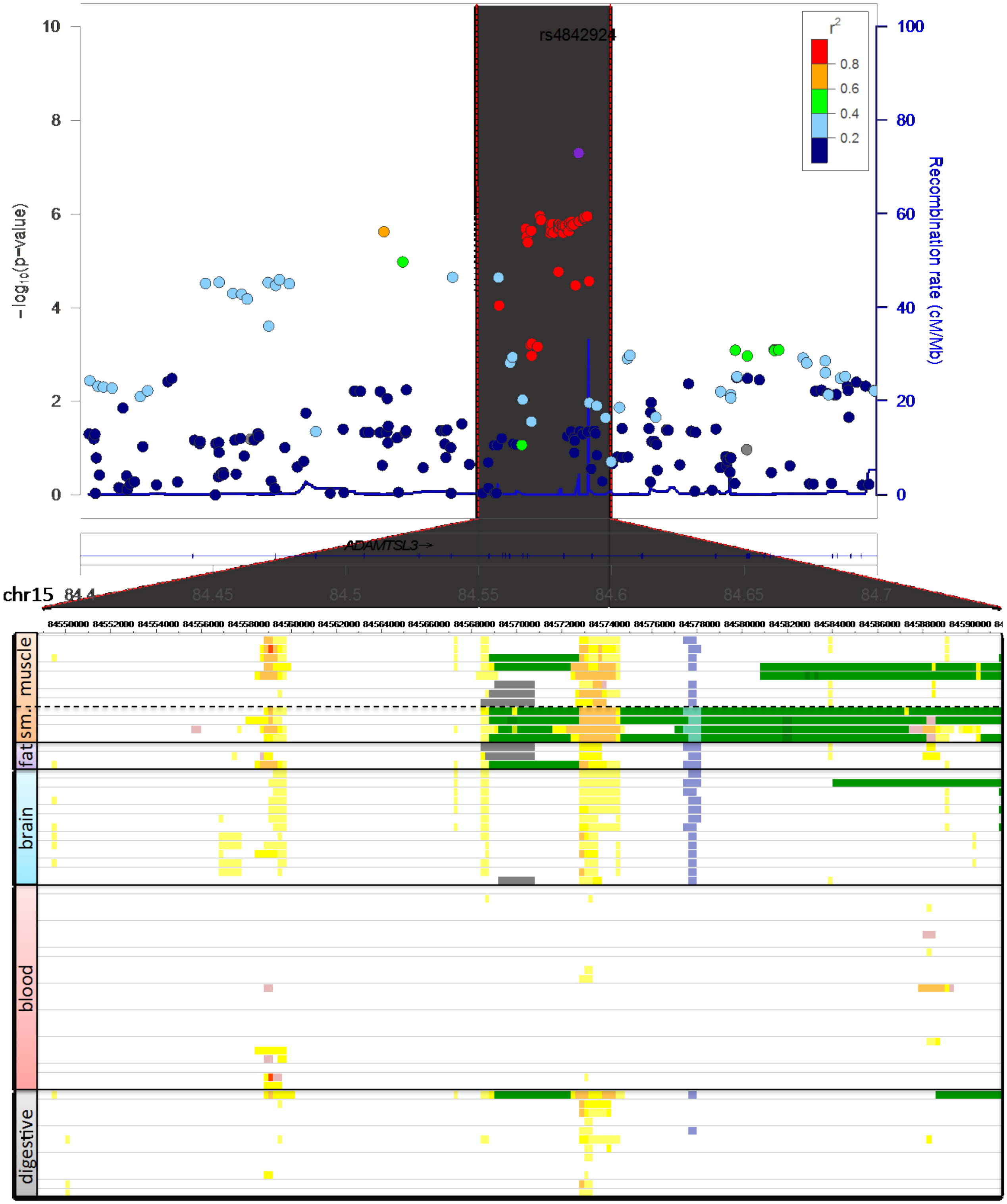

Supplementary Fig. 12: Regional plot of rs4842924 associated with whole-body lean mass and chromatin states in muscle, smooth muscle (sm.), fat, brain, blood, and gastrointestinal tract tissues

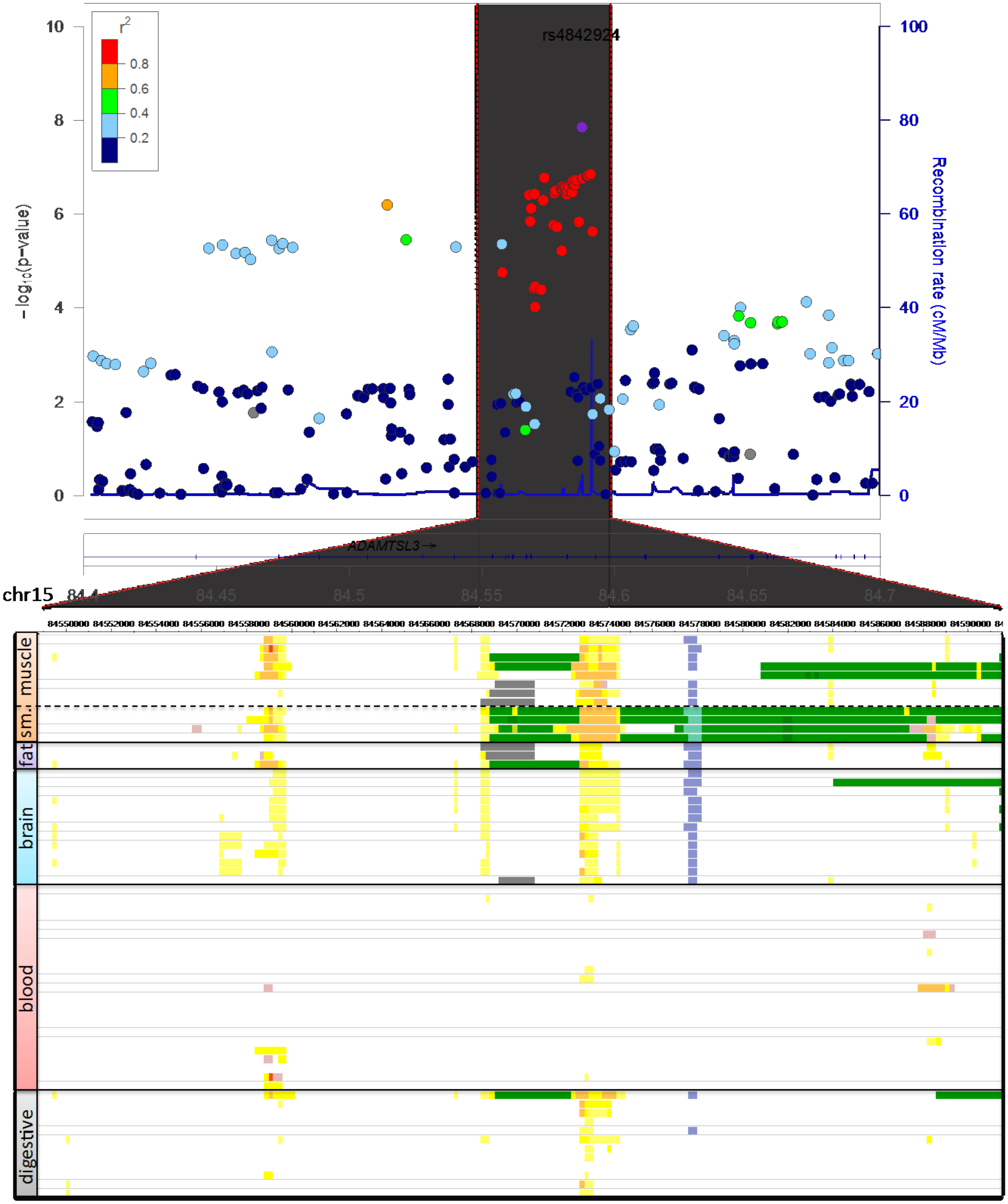

Supplementary Fig. 13: Regional plot of rs9936385 associated with whole-body lean mass and chromatin states in muscle, smooth muscle (sm.), fat, brain, blood, and gastrointestinal tract tissues

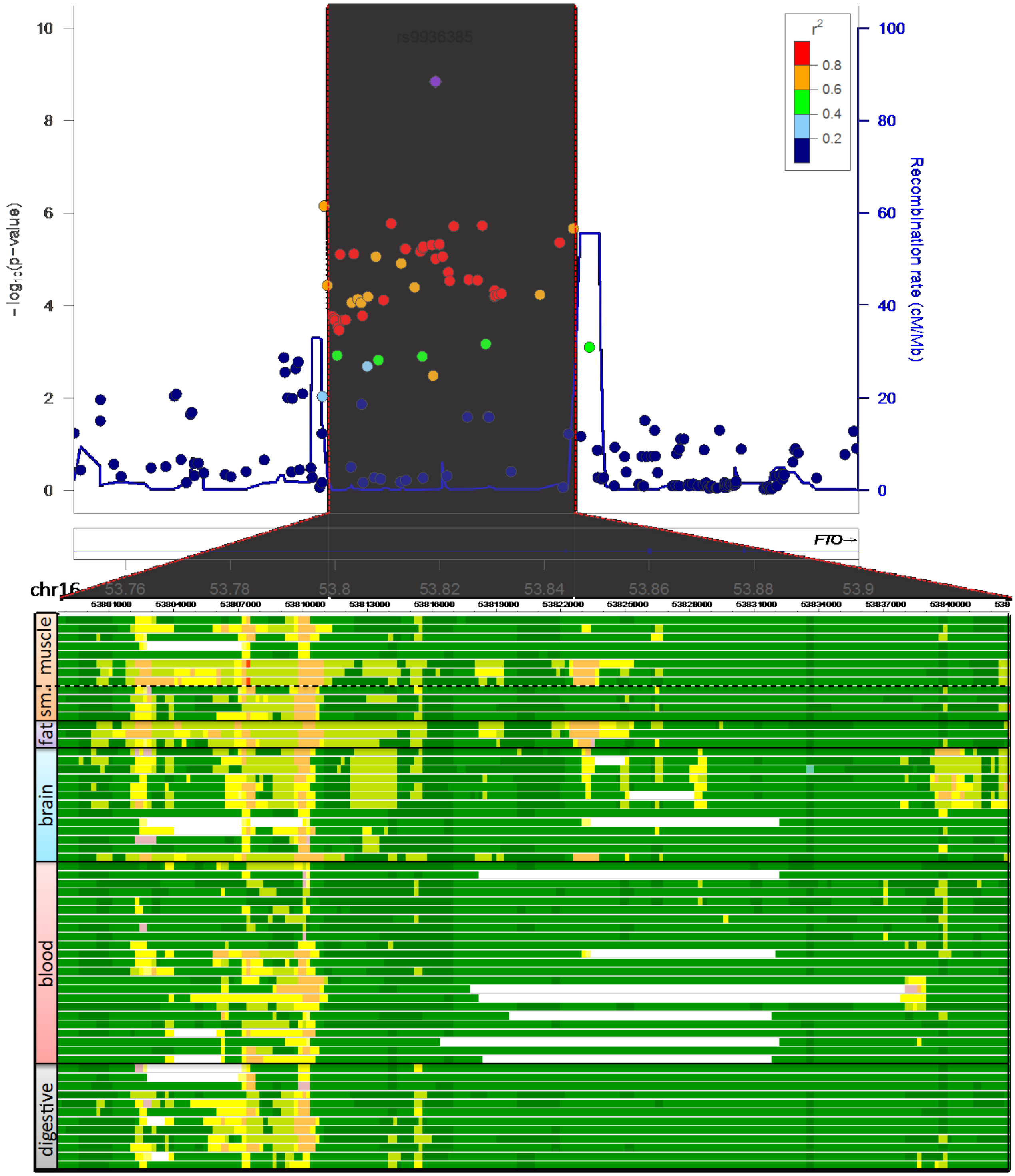

Supplementary Fig. 14: Gene expression in skeletal muscle from the GTEx Study according to genotype. The Y-axis is the rank normalized gene expression.

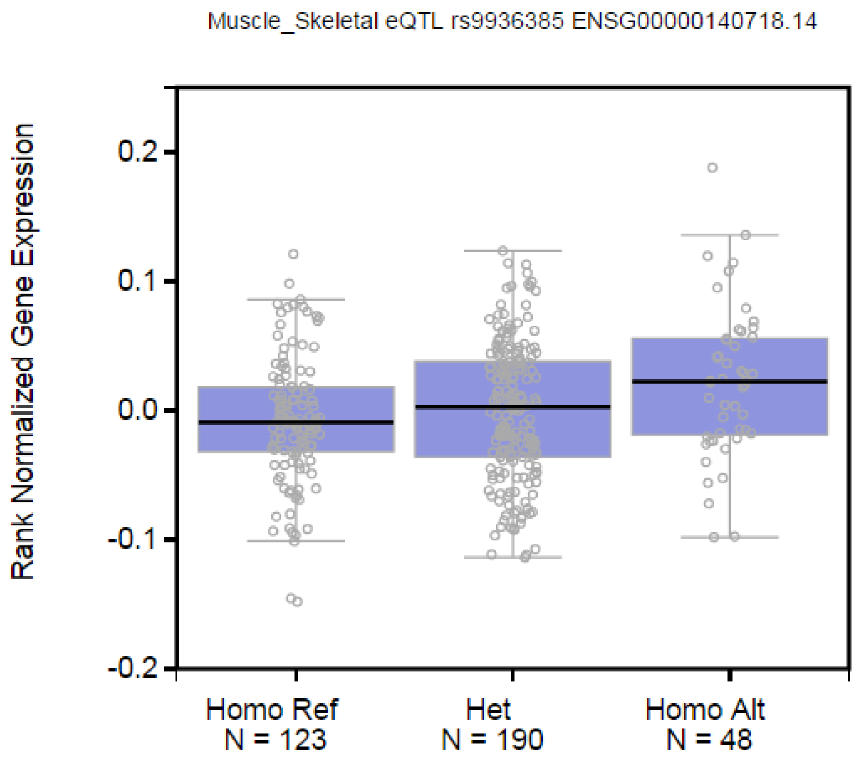

Supplementary Tables

Supplementary Table 1: Discovery and replication meta-analyses for all 16 SNPs taken into replication including replication samples of European ancestry and replication samples of both European and non-European ancestry (bold P-values indicate SNPs successfully replicated).

Whole Body Lean Mass

						Discovery (n=38,292)			Replication EU (n=47,227)			Combined EU (n=85,519)			Combined all (n=101,767)			Mantra	
SNP ID	Chrom	Position	Gene	Allele ½	EAF	Beta	SE	P-value	Beta	SE	P-value	Beta	SE	P-value	Beta	SE	P-value	BF	HetProb
rs2943656	2	226830162	*IRSI*	A/G	0.38	-0.17	0.03	$2.5X10^{-7}$	-0.13	0.03	$8.0X10^{-6}$	-0.14	0.02	**$1.5X10^{-11}$**	-0.14	0.02	$1.5x10^{-11}$	9.5	0.13
rs9991501	4	88477507	*HSD17B11*	T/C	0.04	-0.61	0.01	$2.9x10^{-8}$	-0.26	0.08	$1.9x10^{-3}$	-0.39	0.07	**$5.8x10^{-9}$**	NA	NA	NA	NA	NA
rs2287926	5	82851164	*VCAN*	A/G	0.12	0.24	0.05	$8.6x10^{-7}$	0.15	0.04	$8.5x10^{-4}$	0.19	0.03	**$7.5x10^{-9}$**	0.14	0.03	$5.0x10^{-7}$	5.2	0.36
rs4842924	15	82378611	*ADAMTSL3*	T/C	0.52	-0.17	0.03	$1.4x10^{-7}$	-0.08	0.03	$3.9x10^{-3}$	-0.12	0.02	**$1.4x10^{-8}$**	-0.12	0.02	$7.1x10^{-10}$	7.8	0.16
rs9936385	16	52376670	*FTO*	T/C	0.61	-0.17	0.03	$1.1x10^{-6}$	-0.11	0.03	$1.6x10^{-4}$	-0.14	0.02	**$1.4x10^{-9}$**	-0.15	0.02	$6.9x10^{-13}$	10.6	0.24
rs1110043	1	113037944	*MOV10*	A/G	0.47	-0.15	0.03	$1.8x10^{-6}$	0.01	0.03	$8.3x10^{-1}$	-0.07	0.02	$7.0x10^{-4}$	-0.08	0.02	$5.7x10^{-5}$	3.2	0.27
rs2146098	1	184356993	*HMCN1*	A/G	0.65	0.16	0.03	$1.5x10^{-6}$	0.01	0.04	$8.0x10^{-1}$	0.09	0.02	$1.5x10^{-4}$	0.06	0.02	9.5x10-3	1.3	0.66
rs2999156	1	113048650	*RHOC*	C/G	0.56	-0.17	0.03	$4.0x10^{-7}$	0	0.04	$9.3x10^{-1}$	-0.09	0.02	$1.7x10^{-4}$	-0.09	0.02	1.1x10-5	3.7	0.17
rs10223402	6	116004392	*FRK*	A/G	0.17	0.24	0.05	$4.1x10^{-7}$	0.04	0.04	$3.4x10^{-1}$	0.12	0.03	$6.2x10^{-5}$	0.11	0.03	$3.0x10^{-4}$	2.2	0.21
rs7795758	7	133751491	*AKR1B1*	T/G	0.73	0.17	0.04	$1.9x10^{-6}$	0	0.03	$9.4x10^{-1}$	0.08	0.02	$8.6x10^{-4}$	0.07	0.02	$1.6x10^{-3}$	1.8	0.53
rs9641123	7	93035668	*CALCR*	C/G	0.43	0.17	0.03	$4.8x10^{-7}$	0.06	0.03	$3.3x10^{-2}$	0.11	0.02	$9.8x10^{-7}$	0.09	0.02	$4.3x10^{-6}$	4.1	0.26
rs1028883	13	73006588	*KLF12*	T/G	0.43	-0.16	0.03	$3.2x10^{-7}$	-0.04	0.03	$2.0x10^{-1}$	-0.1	0.02	$8.8x10^{-6}$	-0.09	0.02	$7.1x10^{-6}$	3.9	0.13
rs7206790	16	52355409	*FTO*	C/G	0.54	-0.19	0.04	$6.0x10^{-7}$	-0.06	0.03	$4.7x10^{-2}$	-0.11	0.02	$4.5x10^{-6}$	-0.12	0.02	$5.6x10^{-8}$	6.1	0.56

Appendicular Lean Mass

						Discovery (n=28,330)			Replication EU (n=42,360)			Combined EU (n=70,690)			Combined all (n=73,420)			Mantra	
SNP ID	Chrom	Position	Gene	Allele ½	EAF	Beta	SE	P-value	Beta	SE	P-value	Beta	SE	P-value	Beta	SE	P-value	BF	HetProb
rs2943656	2	226830162	*IRS1*	A/G	0.38	-0.10	0.02	$1.1x10^{-6}$	-0.06	0.01	$2.2x10^{-5}$	-0.07	0.01	**$2.9x10^{-10}$**	-0.07	0.01	$1.8x10^{-10}$	8.3	0.09
rs2287926	5	82851164	*VCAN*	A/G	0.13	0.14	0.03	$8.1x10^{-7}$	0.08	0.02	$3.5x10^{-4}$	0.10	0.02	**$4.5x10^{-9}$**	0.10	0.02	$1.9x10^{-8}$	6.4	0.18
rs4842924	15	82378611	*ADAMTSL3*	T/C	0.52	-0.09	0.02	$1.2x10^{-6}$	-0.05	0.02	$1.6x10^{-3}$	-0.06	0.01	**$5.0x10^{-8}$**	-0.06	0.01	$2.8x10^{-8}$	6.2	0.15
rs9991501	4	88477507	*HSD17B11*	T/C	0.04	-0.33	0.06	$9.7x10^{-8}$	-0.12	0.04	$5.8x10^{-3}$	-0.19	0.04	$1.1x10^{-7}$	NA	NA	NA	NA	NA
rs10223402	6	116004392	*FRK*	A/G	0.17	0.15	0.03	$3.3x10^{-7}$	0.04	0.02	$6.0x10^{-2}$	0.08	0.02	$6.7x10^{-6}$	0.08	0.02	$8.8x10^{-6}$	4	0.18
rs1991642	6	122944689	*PKIB*	C/G	0.72	0.12	0.02	$4.7x10^{-8}$	-0.02	0.02	$1.8x10^{-1}$	0.03	0.01	$2.9x10^{-2}$	0.03	0.01	$3.3x10^{-2}$	1.9	0.96
rs4135187	9	112054589	*TXN*	A/T	0.91	0.21	0.04	$1.3x10^{-6}$	-0.08	0.03	$2.2x10^{-2}$	0.03	0.03	$2.2x10^{-1}$	0.03	0.03	$2.3x10^{-1}$	0.1	0.51
rs4345856	10	66600080	*CTNNA3*	T/C	0.87	0.15	0.03	$1.9x10^{-6}$	0.00	0.02	$9.9x10^{-1}$	-0.05	0.02	$5.0x10^{-3}$	-0.05	0.02	$5.0x10^{-3}$	1.3	0.39

*SNP not polymorphic in non-Europeans

NA: not applicable

BF: log_{10} (Bayes Factor) using the Mantra program[24]

Het Prob: Heterogeneity probability using the Mantra program[24]

Supplementary Table 2 – Results for successfully replicated SNPs according to sex with test for interaction

Whole Body Lean Mass

						Women (n=22,705)			Men (n=15,587)			
SNP ID	Chrom	Position	Closest Gene	Allele 1/2	EAF	Beta	SE	P-value	Beta	SE	P-value	P interaction*
rs2943656	2	2.27E+08	*IRS1*	A/G	0.38	-0.09	0.04	$9.0x10^{-3}$	-0.25	0.06	$7.0x10^{-6}$	$1.9x10^{-2}$
rs9991501	4	88477507	*HSD17B11***	T/C	0.04	-0.45	0.28	$1.1x10^{-1}$	-1.03	0.65	$1.1x10^{-1}$	$4.1x10^{-1}$
rs2287926	5	82851164	*VCAN*	A/G	0.12	0.19	0.05	$4.5x10^{-4}$	0.28	0.08	$6.5x10^{-4}$	$3.3x10^{-1}$
rs4842924	15	82378611	*ADAMTSL3*	T/C	0.52	-0.12	0.04	$6.3x10^{-4}$	-0.23	0.05	$1.9x10^{-5}$	$8.7x10^{-2}$
rs9936385	16	52376670	*FTO*	T/C	0.61	-0.09	0.04	$2.5x10^{-2}$	-0.24	0.06	$4.5x10^{-5}$	$2.8x10^{-2}$

Appendicular Lean Mass

						Women (n=17,271)			Men (n=11,059)			
SNP ID	Chrom	Position	Closest Gene	Allele 1/2	EAF	Beta	SE	P-value	Beta	SE	P-value	P interaction*
rs2943656	2	2.27E+08	*IRS1*	A/G	0.38	-0.05	0.02	$1.9x10^{-2}$	-0.15	0.04	$1.3x10^{-5}$	$8.6x10^{-3}$
rs2287926	5	82851164	*VCAN*	A/G	0.13	0.13	0.03	$1.9x10^{-5}$	0.15	0.05	$3.2x10^{-3}$	$6.6x10^{-1}$
rs4842924	15	82378611	*ADAMTSL3*	T/C	0.52	-0.07	0.02	$2.0x10^{-4}$	-0.12	0.03	$6.1x10^{-4}$	$2.6x10^{-1}$

* P interaction significant when P < 0.05/8 = 0.00625

**for HSD17B11 the sex specific analysis included less people because the rare MAF limited power to examine sex specific effects

Supplementary Table 3 - Discovery meta-analyses for the genome wide significant SNPs stratified by technique (BIA vs DXA) with test for interaction

Whole Body Lean Mass

						DXA (n=21,074)			BIA (n=17,218)			
SNP ID	Chrom	Position	Closest Gene	Allele 1/2	EAF	Beta	SE	P-value	Beta	SE	P-value	P interaction*
rs2943656	2	226830162	*IRS1*	A/G	0.38	-0.21	0.05	$7.8x10^{-5}$	-0.14	0.04	$5.7x10^{-4}$	$3.4x10^{-1}$
rs9991501	4	88477507	*HSD17B11*	T/C	0.04	-0.59	0.15	$5.7x10^{-5}$	-0.63	0.16	$1.3x10^{-4}$	$8.8x10^{-1}$
rs2287926	5	82851164	*VCAN*	A/G	0.12	0.20	0.08	$8.5x10^{-3}$	0.26	0.06	$2.7x10^{-5}$	$5.6x10^{-1}$
rs4842924	15	82378611	*ADAMTSL3*	T/C	0.52	-0.15	0.05	$3.2x10^{-3}$	-0.18	0.04	$1.1x10^{-5}$	$6.5x10^{-1}$
rs9936385	16	52376670	*FTO*	T/C	0.61	-0.24	0.05	$7.1x10^{-6}$	-0.12	0.05	$1.0x10^{-2}$	$8.0x10^{-2}$

Appendicular Lean Mass

						DXA (n=21,074)			BIA (n=17,218)			
SNP ID	Chrom	Position	Closest Gene	Allele 1/2	EAF	Beta	SE	P-value	Beta	SE	P-value	P interaction*
rs2943656	2	226830162	*IRS1*	A/G	0.38	-0.10	0.03	$1.6x10^{-3}$	-0.09	0.03	$2.1x10^{-4}$	$9.5x10^{-1}$
rs2287926	5	82851164	*VCAN*	A/G	0.13	0.14	0.05	$1.5x10^{-3}$	0.14	0.04	$1.6x10^{-4}$	$9.3x10^{-1}$
rs4842924	15	82378611	*ADAMTSL3*	T/C	0.52	-0.11	0.03	$2.5x10^{-4}$	-0.08	0.03	$1.1x10^{-3}$	$4.8x10^{-1}$

* P interaction significant when P < 0.05/8 = 0.00625

Supplementary Table 4: Study design and measurement technique of lean mass

Study	Full Name	Study Design	Lean Mass Technique	DXA or BIA Device: Manufacturer, Model	Country of Origin	Ethnicity	IRB Clearance and Informed Consent	References
Discovery Cohorts								
1. AGES	Age, Gene/Environment Susceptibility Reykjavik Study (AGES-Reykjavik)	Population-based Cohort	BIA	Xitron Technologies,Hydra ECF/ICF Model 4200	Iceland	European Ancestry	Yes	Harris, TB, Launer LJ, Eiriksdottier G, et al. Age, Gene/Environment Susceptibility -Reykjavik Study: Multidisciplinary Applied Phenomics. AJE. 2007; 165:1076-1087.
2. Amish	Amish Family Osteoporosis Study	Family Study	DXA	Hologic 4500W	United States	European Ancestry/Old Order Amish	Yes	Streeten EA, JBMR 2006 Sep; 21(9): 1433-42. Quantitative trait loci for BMD identified by autosome-wide linkage scan to chromosomes 7q and 21q in men from the Amish Family Osteoporosis Study.
3. CHS	Cardiovascular Health Study	Random Cohort	DXA	Hologic Hologic QDR-2000	USA	European and African Ancestry	Yes	Fried LP, Borhani NO, Enright P et al. The Cardiovascular Health Study: Design and rationale. Ann Epidemiol 1991;1:263–276
4. CoLaus	Cohorte Lausannoise	Population-based Cohort	BIA	BIA using Bodystat® 1500 analyzer (Isle of Man, British Isles)	Switzerland	European Ancestry	Yes	Firmann et al 2008 BMC Cardiovasc Disord. (PMID18366642)
5. deCODE	University Hospital Bone Mineral Study and Study on genetics of bone mass	Population	DXA	Hologic QDR4500A	Iceland	European Ancestry	Yes	Gudmundsdottir SL,Indridason OS,Franzson and Sigurdsson G, J Clin Densitom 2005,8,80-86; Steingrimsdottir L,Gunnarsson,Indridason OS, Franzson L, Sigurdsson G, JAMA 2005,294,2336 -2341; Sigurdsson G, Halldorsson BV, Styrkarsdottir U, Kristjansson K, Stefansson K, JBMR 2008,10,1584-190; Strykarsdottir et al, Nat Genet 2009, 41, 15-7
6. EPIC Obesity Study	The European Prospective Investigation of Cancer Obesity Study	Population-based	BIA	Tanita 531; Tanita Europe GmbH, Sindelfingen, Germany	United Kingdom	European Ancestry	Yes	Day N, Oakes S, Luben R, Khaw KT, Bingham S, Welch A, Wareham N. EPIC-Norfolk: study design and characteristics of the cohort. European Prospective Investigation of Cancer. Br J Cancer. 1999 Jul; 80 Suppl 1:95-103.
7. ERF	Erasmus Rucphen Family Study	Population-based	DXA	Lunar Prodigy by GE Healthcare	The Netherlands	European Ancestry	Yes	Aulchenko YS, Heutink P, Mackay I, Bertoli-Avella AM, Pullen J, Vaessen N, Rademaker TA, Sandkuijl LA, Cardon L, Oostra B , van Duijn CM (2004). Linkage disequilibrium in young genetically isolated Dutch population. Eur J Hum Genet. 12, 527-534
8. Fenland	The Fenland Study	Population-based	DXA	Lunar Prodigy Advanced fan beam scanner; GE Healthcare, Bedford, UK	United Kingdom	European Ancestry	Yes	Rolfe Ede L, et al. Association between birth weight and visceral fat in adults. Am J Clin Nutr. 2010 Aug; 92(2):347-52.<br>Willer C et al. Six new loci associated with body mass index highlight a neuronal influence on body weight regulation. Nat Gen. 2009 Jan; 41(1):25-34.
9. FRAM	The Framingham Heart Study	Population-based	BIA	RJL Systems BIA-101	United States	European Ancestry	Yes	Roubenoff R, Baumgartner RN, Harris TB, Dallal GE, Hannan MT, Economos CD, Stauber PM, Wilson PWF, Harris TB, Kiel DP. Application of bioelectric impedance analysis to elderly populations. J Gerontol 1997; 52(3):M161-8.
10. GOOD	Gothenburg Osteoporosis	Prospective	DXA	Lunar Prodigy, GE Lunar	Sweden	European	Yes	Lorentzon M, Swanson C, Andersson N,

	and Obesity Determinants Study	Population-based Cohort		Corp		Ancestry		Mellstrom D, Ohlsson C (2005) Free testosterone is a positive, whereas free estradiol is a negative, predictor of cortical bone size in young Swedish men: the GOOD study. J Bone Miner Res 20: 1334-1341.
11. HABC	Health Aging and Body Composition Study		DXA	Hologic QDR 4500	United States	European Ancestry	Yes	Goodpaster BH, Carlson CL, Visser M, Kelley DE, Scherzinger A, Harris TB, Stamm E, Newman AB. Attenuation of skeletal muscle and strength in the elderly: The Health ABC Study. J Appl Physiol. 2001; 90: 2157-2165.
12. Indiana	Indiana Sisters Osteoporosis Study	Healthy sibling Pairs	DXA	Lunar DPXL and Prodigy by GE Healthcare	United States	European Ancestry	Yes	Koller DL, Ichikawa S, Lai D, Padgett LR, Doheny KF, Pugh E, Paschall J, Hui SL, Edenberg HJ, Xuei X, Peacock M, Econs MJ, Foroud T. Genome-wide association study of bone mineral density in premenopausal European-American women and replication in African-American women. J Clin Endocrinol Metab 2010; 95(4): 1802-9. PMID:20164292
13. Kora F3	Cooperative Health Research in the Region of Augsburg	Population based	BIA	DATA-INPUT GmbH BIA 2000-S	Germany	European Ancestry	Yes	Wichmann HE, Gieger C and Illig T. (2005). KORA-gen-resource for population genetics, controls and a broad spectrum of disease phenotypes. Gesundheitswesen, 67: S26_S30.
14. Kora F4	Cooperative Health Research in the Region of Augsburg	Population based	BIA	DATA-INPUT GmbH BIA 2000-S	Germany	European Ancestry	Yes	Wichmann HE, Gieger C and Illig T. (2005). KORA-gen-resource for population genetics, controls and a broad spectrum of disease phenotypes. Gesundheitswesen, 67: S26_S30.
15 & 16. Lolipop*	The London Life Sciences Prospective Population Study	Prospective Population-based Cohort Studies	BIA	Instrument for fat%: Tanita bioimpedance scales (TBF-401A)	UK	European Ancestry	Yes	1. Kooner JS, Chambers JC, Aguilar-Salinas CA, et al. Genome-wide scan identifies variation in MLXIPL associated with plasma triglycerides. Nat Genet 2008, 40:149-151. 2. Yuan X, Waterworth D, Perry JR, et al. Population-based genome-wide association studies reveal six loci influencing plasma levels of liver enzymes. Am J Hum Genet 2008, 83:520-528.
17. RS I	The Rotterdam Study I	Prospective Population-based Cohort Studies	DXA	Lunar Prodigy by GE Healthcare	The Netherlands	European Ancestry	Yes	Hofman A, Bruselle GG, Murad D, van Duijn CM, Franco OH, Goedegebure A, Ikram MA, Klaver CC, Nijsten TE, Peeters, RP, Stricker BH, Tiemeier HW, Uitterlinden AG, Vernooij MW . The Rotterdam Study: 2016 objectives and design update. Eur J Epidemiol. 2015 Aug; 30(8):661-708.
18. RS II	The Rotterdam Study II	Prospective Population-based Cohort Studies	DXA	Lunar Prodigy by GE Healthcare	The Netherlands	European Ancestry	Yes	Hofman A, Bruselle GG, Murad D, van Duijn CM, Franco OH, Goedegebure A, Ikram MA, Klaver CC, Nijsten TE, Peeters, RP, Stricker BH, Tiemeier HW, Uitterlinden AG, Vernooij MW . The Rotterdam Study: 2016 objectives and design update. Eur J Epidemiol. 2015 Aug; 30(8):661-708.
19 & 20. TUK*	Twins UK	Prospective Population-based Twin Cohort Study	DXA	Hologic Inc, Bedford, MA	UK	European Ancestry	Yes	Moayyeri A, Hammond CJ, Valdes AM, Spector TD. Cohort Profile: TwinsUK and Healthy Ageing Twin Study. Int J Epidemiol. 2012 Jan 9. [Epub ahead of print]
REPLICATION COHORTS								
1. AGES	Age, Gene/Environment	Population-	BIA	Xitron Technologies,Hydra	Iceland	European	Yes	Harris, TB, Launer LJ, Eiriksdottier G, et al. Age,

	Susceptibility Reykjavik Study (AGES-Reykjavik)	based Cohort		ECF/ICF Model 4200		Ancestry		Gene/Environment Susceptibility -Reykjavik Study: Multidisciplinary Applied Phenomics. AJE. 2007; 165:1076-1087.
2. BASE-II	Berlin Aging Study II	Prospective Population-based Cohort Study	DXA	Hologic Discovery DXA system; Hologic Inc., Bedford, MA, USA	Germany	European Ancestry	Yes	Bertram L, Böckenhoff A, Demuth I, Düzel S, Eckardt R, Li SC, Lindenberger U, Pawelec G, Siedler T, Wagner GG, Steinhagen-Thiessen E. Cohort profile: The Berlin Aging Study II (BASE-II). Int J Epidemiol. 2014 Jun;43(3):703-12.
3. B-PROOF	B-Vitamins for the Prevention Of Osteoporotic Fractures Study	Randomized Controlled Trial; Baseline Data	DXA	Lunar Prodigy by GE Healthcare; Hologic QDR 4500 Delphi device by Hologic Inc.	The Netherlands	European Ancestry	Yes	van Wijngaarden JP, Dhonukshe-Rutten RAM, van Schoor NM et al. Rationale and design of the B-PROOF study, a randomized controlled trial on the effect of supplemental intake of vitamin B12 and folic acid on fracture incidence. BMC Geriatr 2011; 11:80.
4. CAIFOS	Calcium Intake Fracture Outcome Study	RCT	DXA	Hologic 4500A	Australia	European Ancestry	Yes	Prince RL, Devine A, Dhaliwal SS, Dick IM. Effects of calcium supplementation on clinical fracture and bone structure: results of a 5-year, double-blind, placebo-controlled trial in elderly women. Arch Intern Med. 2006;166(8):869-75.
5. DOPS	Danish Osteoporosis Prevention Study	Prospective Population-based Cohort	DXA	Hologic 1000 and 2000	Denmark	European Ancestry	Yes	Mosekilde L et al. Maturitas 1999 31(3) 207-19
6. FamHS	Family Heart Study	Family Based	BIA	RJL bioelectric impedance meter (RJL Systems, Clinton Township, MI)	MA,NC,MN,UT	European Ancestry	Yes	Higgins, M. et al., NHLBI Family Heart Study: objectives and design, Am J Epidemiol 143, 1219–1228 (1996) Kilpeläinen et al. Genetic variation near IRS1 associates with reduced adiposity and an impaired metabolic profile. Nat Genet. 2011; 43:753-60.
7. Genmets controls	Health2000 GenMets Study	Case-Control	BIA	InBody 3.0	Finland	European Ancestry	Yes	Perttilä J, et al. OSBL10, a novel candidate gene for high triglyceride trait in dyslipidemic Finnish subjects, regulates cellular lipid metabolism. J Mol Med 87, 825-835 (2009)
8. Helsinki Birth Cohort	Helsinki Birth Cohort Study	Birth Cohort	BIA	InBody 3.0	Finland	European Ancestry	Yes	Ylihärsilä H, Kajantie E, Osmond C, Forsén T, Barker DJ, Eriksson JG. Birth size, adult body composition and muscle strength in later life. Int J Obes (Lond) 2007;Sep;31(9):1392-9
9 &10. Johnson County Study*	Johnston County Osteoarthritis Project	Prospective Population-based Cohort Studies	DXA	Manufacturer: Hologic Model: Delphi	USA	European Ancestry and African American	Yes	Abbate LM, Stevens J, Schwartz TA, Renner JB, Helmick CG, Jordan JM. Anthropometric measures, body composition, body fat distribution, and knee osteoarthritis in women. Obesity (Silver Spring). 2006 Jul;14(7):1274-81.
11 & 12. KoGES*	Korean Genome Epidemiology Study	Prospective Population-based Cohort Studies	DXA and BIA	Lunar Prodigy by GE Healthcare and Inbody 3.0 by Biospace, Korea	Korea	Koreans	Yes	Park B, Yang JJ, Yang JH, Kim J, Cho LY, Kang D, Shin C, Hong YS, Choi BY, Kim SS, Park MS, Park SK. Reliability and data integration of duplicated test results using two bioelectrical impedence analysis machines in the Korean Genome and Epidemiology Study. J Prev Med Public Health. 2010 Nov;43(6):479-85.
13. Kora F3	Cooperative Health Research in the Region of Augsburg	Population Based	BIA	DATA-INPUT GmbH BIA 2000-S	Germany	European Ancestry	Yes	Wichmann HE, Gieger C and Illig T. (2005). KORA-gen-resource for population genetics, controls and a broad spectrum of disease phenotypes. Gesundheitswesen, 67: S26_S30.

14. Kora F4	Cooperative Health Research in the Region of Augsburg	Population Based	BIA	DATA-INPUT GmbH BIA 2000-S	Germany	European Ancestry	Yes	Wichmann HE, Gieger C and Illig T. (2005). KORA-gen-resource for population genetics, controls and a broad spectrum of disease phenotypes. Gesundheitswesen, 67: S26_S30.
15. LOLIP-REP-IA610	The London Life Sciences Prospective Population Study	Prospective Population-based Cohort Studies	BIA	Instrument for fat%: Tanita bioimpedance scales (TBF-401A)	UK	Indian Asians	Yes	Chambers JC, Zhang W, Li Y, et al. Genome-wide association study identifies variants in TMPRSS6 associated with hemoglobin levels. Nat Genet 2009, 41:1170-1172. 2. Chambers JC, Zhang W, Zabaneh D, et al. Common genetic variation near melatonin receptor MTNR1B contributes to raised plasma glucose and increased risk of type 2 diabetes among Indian Asians and European Caucasians. Diabetes 2009, 58:2703-2708.
16. LOLIP-REP-IA_I	The London Life Sciences Prospective Population Study	Prospective Population-based Cohort Studies	BIA	Instrument for fat%: Tanita bioimpedance scales (TBF-401A)	UK	Indian Asians	Yes	Chambers JC, Elliott P, Zabaneh D, et al. Common Genetic variation near MC4R is associated with waist circumference and insulin resistance. Nat Genet 2008, 40: 716-718.
17. LOLIP-REP-IA_P	The London Life Sciences Prospective Population Study	Prospective Population-based Cohort Studies	BIA	Instrument for fat%: Tanita bioimpedance scales (TBF-401A)	UK	Indian Asians	Yes	Kooner JS, Chambers JC, Aguilar-Salinas CA, et al. Genome-wide scan identifies variation in MLXIPL associated with plasma triglycerides. Nat Genet 2008, 40:149-151.
18. METSIM	METabolic Syndrome In Men	Prospective Population-based Cohort	BIA	BIA 101- Body Impedance Analyzer	Finland	European Ancestry	Yes	Stancakova A, et al. Association of 18 confirmed susceptibility loci for type 2 diabetes with indices of insulin release, proinsulin conversion, and insulin sensitivity in 5,327 nondiabetic Finnish men. Diabetes. 2009; 58:2129–36
19. MrOS Sweden	Osteoporotic Fractures in Men study Sweden	Prospective Population-based Cohort	DXA	Lunar Prodigy, GE Lunar Corp and Hologic QDR, Hologic 4500/A-Delphi	Sweden	European Ancestry	Yes	Mellstrom D, Johnell O, Ljunggren O, Eriksson AL, Lorentzon M, et al. (2006) Free testosterone is an independent predictor of BMD and prevalent fractures in elderly men: MrOS Sweden. J Bone Miner Res 21: 529-535.
20. MrOS U.S.	Osteoporotic Fractures in Men Study	Prospective Cohort	DXA	Hologic QDR 4500	US	European Ancestry	Yes	1. Blank JB, Cawthon PM, Carrion-Petersen ML, Harper L, Johnson JP, Mitson E, et al. Overview of recruitment for the osteoporotic fractures in men study (MrOS). Contemp Clin Trials 2005; 26:557-68. 2. Orwoll E, Blank JB, Barrett-Connor E, Cauley J, Cummings S, Ensrud K, et al. Design and baseline characteristics of the osteoporotic fractures in men (MrOS) study--a large observational study of the determinants of fracture in older men. Contemp Clin Trials 2005; 26:569-85.
21. OPRA	Osteoporosis Prospective Risk Assessment study	Prospective Population-based Cohort Study	DXA	Lunar DPX-L Lunar corporation and GE Healthcare	Sweden	European Ancestry	Yes	Gerdhem P, H Brandstrom, F Stiger, K Obrant, H Melhus, O Ljunggren, A Kindmark, and K Akesson (2004) Association of the COL1A 1 Sp1 binding site polymorphism to femoral neck bone mineral density and wrist fracture in 1044 elderly Swedish women Calcif Tissue Int 74(3): 264-269
22. ORCADES	Orkney Complex Disease Study	Isolated Population-based Cohort	DXA	Hologic QDR4500	Scotland	European Ancestry	Yes	McQuillan et al (2008), Runs of homozygosity in European populations Am J Hum Genet 83:359-72

23. PEAK 25	PEAK25	Population-based Cohort Study	DXA	Lunar DPX-L Lunar corporation and GE Healthcare	Sweden	European Ancestry	Yes	McGuigan FE, Larzenius E, Callreus M, Gerdhem P, Luthman H, et al. (2007) Variation in the BMP2 gene: bone mineral density and ultrasound in young adult and elderly women. Calcif Tissue Int 81: 254-262
24. PIVUS	Prospective Investigation of the Vasculature in Uppsala Seniors	Population-based Cohort Study	DXA	Lunar Prodigy by GE Healthcare	Sweden	European Ancestry	Yes	Michaëlsson K, Lind L, Frystyk J, Flyvbjerg A, Gedeborg R, Berne C, Zethelius B, Mallmin H, Söderberg S, Melhus H. Serum adiponectin in elderly men does not correlate with fracture risk. J Clin Endocrinol Metab. 2008 Oct;93(10):4041-7.
25. RISC	Relationship between Insulin Sensitivity and Cardiovascular disease risk	Prospective Population-based Cohort Studies	BIA	Tanita bioimpedance TBF-300a body composition analyser	Austria, Denmark, Finland, France, Germany, Greece, The Netherlands, Ireland, Italy, Sweden, Spain, Switzerland, United Kingdom and Serbia and Montenegro	European Ancestry	Yes	Hills SA, Balkau B, Coppack SW, Dekker JM, Mari A, Natali A, et al. The egir-risc study (the european group for the study of insulin resistance: Relationship between insulin sensitivity and cardiovascular disease risk): I. Methodology and objectives. Diabetologia 2004; 47:566-570.
26. Rotterdam III	The Rotterdam Study III	Prospective Population-based Cohort Studies	DXA	Lunar Prodigy by GE Healthcare	The Netherlands	European Ancestry	Yes	Hofman A, Bruselle GG, Murad D, van Duijn CM, Franco OH, Goedegebure A, Ikram MA, Klaver CC, Nijsten TE, Peeters, RP, Stricker BH, Tiemeier HW, Uitterlinden AG, Vernooij MW . The Rotterdam Study: 2016 objectives and design update. Eur J Epidemiol. 2015 Aug; 30(8):661-708.
27. Rush Memory and Aging Project	Memory and Aging Project	Prospective Population-based Cohort Studies	BIA	Portable Body Comp Scale by American Weights & Measure, California	USA	European Ancestry	YES	Bennett DA, Schneider JA, Buchman AS, Barnes LL, Boyle PA, Wilson RS. Overview and Findings from the Rush Memory and Aging Project. Curr Alzheimer Res. 2012 Jul 1; 9(6):646-63.
28. SHIP 2	Study of Health In Pomerania 2	Population-based Cohort Study	BIA	Nutriguard M by Data Input	Germany	European Ancestry	YES	Volzke H, Alte D, Schmidt CO, et al. Cohort Profile: The Study of Health in Pomerania. Int J Epidemiol. Apr 2011;40(2):294-307.
29. SHIP TREND	Study of Health In Pomerania TREND	Population-based Study	BIA	Nutriguard M by Data Input	Germany	Caucasian	YES	Volzke H, Alte D, Schmidt CO, et al. Cohort Profile: The Study of Health in Pomerania. Int J Epidemiol. Apr 2011; 40(2):294-307.
30. SOF	Study of Osteoporotic Fractures	Prospective Cohort	BIA	Valhalla 1990B Bio-Resistance Body Composition Analyzers	US	European Ancestry	YES	Cummings SR, Black DM, Nevitt MC, Browner WS, Cauley JA, Genant HK, et al. Appendicular bone density and age predict hip fracture in women. The Study of Osteoporotic Fractures Research Group. JAMA.1990; 263:665-8.
31. ULSAM	Uppsala Longitudinal Study of Adult Men	Population-based Cohort Study	DXA	Lunar Prodigy by GE Healthcare	Sweden	European Ancestry	Yes	Byberg L, Gedeborg R, Cars T, Sundström J, Berglund L, Kilander L, Melhus H, Michaëlsson K. Prediction of fracture risk in men: a cohort study. J Bone Miner Res. 2012 Apr;27(4):797-807
32. VIS	CROATIA-Vis	Isolated Population-based Cohort	BIA	BC-531 Body Composition Monitor,	Croatia	European Ancestry	Yes	Vitart V, Biloglav Z, Hayward C, Janicijevic B, Smolif-Narancic N, Barac L, Pericic M, Martinovic Klaric I, Skaric-Juric T, Barbalic M, Polasek O, Kolcic I, Carothers A, Rudan P, Hastie

								N, Wright A, Campbell H, Rudan I. 3000 years of solitude: extreme differentiation in the island isolates of Dalmatia, Croatia. European Journal of Human Genetics (2006) 14, 478–487
33. WHI	Womens Health Initiative	Prospective Cohort	DXA	Hologic	US	European Ancestry	YES	Jackson et al. The Women's Health Initiative calcium-vitamin D trial: overview and baseline characteristics of participants. Ann Epidemiol 2003;13(suppl):S98–106

*These cohorts provided two subcohorts for which overall characteristics have been combined

Supplementary Table 5: Characteristics of the study populations from discovery and replication cohorts

Study	Gender	N with geno- and phenotype	Age (yrs) Mean (SD)	Height (cm) Mean (SD)	Weight (kg) Mean (SD)	BMI (kg/m2) Mean (SD)	Total lean mass (kg) mean (SD)	Appendicular lean mass (kg) mean (SD)	Total fat mass (kg) mean (SD)	Fat% mean (SD)
Discovery Cohorts										
1. AGES	Males	1032	76.40 (5.29)	176.39 (6.14)	82.67 (13.16)	26.84 (3.77)	63.99 (7.55)	40.67 (4.59)	18.69 (6.99)	21.98 (5.48)
	Females	1386	75.92 (5.43)	160.90 (5.74)	70.21 (13.21)	27.10 (4.80)	45.85 (6.29)	35.23 (4.65)	24.36 (7.53)	33.99 (4.95)
	Combined	2418	76.13 (5.37)	167.08 (9.291)	75.53 (14.56)	26.99 (4.39)	53.59 (11.29)	37.55 (5.35)	21.94 (7.82)	28.862 (7.88)
2. Old Order Amish	Males	402	50.6 (15.0)	171.7 (6.3)	78.2 (12.1)	26.5 (3.7)	61.0 (6.9)	27.0 (3.4) *N=374	15.8 (7.3)	19.7 (6.9)
	Females	440	52.1 (14.4)	159.7 (6.0)	72.5 (14.5)	28.4 (5.5)	45.4 (6.3)	18.7 (3.1) *N=423	25.9 (9.1)	35.4 (6.6)
	Combined	842	51.4 (14.7)	165.4 (8.6)	75.2 (13.7)	27.5 (4.8)	52.8 (10.4)	22.6 (5.2) *N=797	21.1 (9.7)	27.9 (10.3)
3. CHS	Males	1252	72.9 (5.6)	173.3 (6.5)	79.4 (12.0)	26.4 (3.6)	51.9 (5.7)	24.3 (3.2)	27.4 (8.6)	33.9 (6.6)
	Females	1938	71.9 (5.2)	159.1 (6.1)	66.7 (13.1)	26.3 (4.9)	41.0 (4.6)	16.4 (2.7)	25.7 (10.1)	37.2 (8.1)
	Combined	3190	72.3 (5.4)	164.7 (9.)	71.7 (14.1)	26.4 (4.4)	45.3 (7.3)	19.5 (4.8)	26.4 (9.6)	35.9 (7.7)
4. CoLaus	Males	2527	52.89 (10.75)	174.99 (7.33)	81.54 (13.37)	26.62 (4.04)	61.62 (8.40)	NA	19.92 (7.56)	23.93 (6.01)
	Females	2835	53.90 (10.70)	162.62 (6.70)	66.46 (13.00)	25.17 (4.91)	42.92 (6.38)	NA	23.54 (9.56)	34.48 (8.25)
	Combined	5362	53.43 (10.73)	168.45 (9.34)	73.57 (15.17)	25.86 (4.58)	51.73 (11.91)	NA	21.84 (8.86)	29.51 (8.98)
5. decode	Males	676	63.06 (14.93)	177.0 (6.7)	86.13 (15.87)	27.41 (4.40)	51.57 (11.07)	28.06 (4.60)	26.19 (99.58)	33.16 (8.42)
	Females	2315	57.35 (15.78)	164.1 (6.3)	70.43 (13.85)	26.14 (4.98)	49.76 (11.07)	18.92 (3.40)	23.00 (81.87)	31.35 (8.09)
	Combined	2991	58.65 (15.78)	167.0 (8.4)	73.98 (15.77)	26.43 (4.88)	50.17 (11.09)	20.99 (5.32)	23.71 (87.19)	31.76 (8.19)
6. EPIC	Males	763	63.28 (8.94)	174.04 (6.64)	80.85 (11.21)	26.62 (3.25)	62.12 (6.11)	NA	19.92 (7.61)	23.64 (6.07)
	Females	928	61.95 (8.75)	161.15 (5.74)	67.90 (11.29)	26.05 (4.26)	40.71 (4.91)	NA	28.35 (11.12)	39.86 (9.26)
	Combined	1691	62.55 (8.86)	166.97 (8.89)	73.74 (12.97)	26.31 (3.85)	50.37 (11.98)	NA	24.55 (10.56)	32.54 (11.35)
7. ERF	Males	906	48.76 (14.47)	174.44 (7.24)	83.01 (14.24)	27.26 (4.22)	57.76 (7.07)	25.60 (3.55)	22.26 (9.01)	25.98 (6.96)
	Females	1191	47.71 (14.46)	161.75 (6.61)	69.20 (13.76)	26.44 (4.93)	39.84 (4.99)	16.53 (2.30)	26.51 (9.97)	37.17 (7.44)
	Combined	2097	48.17 (14.47)	167.23 (9.32)	75.16 (15.55)	26.79 (4.65)	47.58 (10.70)	20.45 (5.35)	24.67 (9.79)	32.34 (9.12)
8. Fenland	Males	554	44.38 (7.35)	177.02 (6.33)	85.41 (12.18)	27.23 (3.4)	57.97 (6.43)	26.96 (3.35)	24.26 (8.06)	27.81 (6.53)
	Females	724	45.27 (7.27)	163.76 (6.12)	70.42 (14.04)	26.25 (5.0)	40.56 (5.07)	17.40 (2.41)	26.96 (10.22)	37.35 (7.58)
	Combined	1278	44.88 (7.31)	169.51 (9.07)	76.92 (15.20)	26.67 (4.4)	48.11 (10.34)	21.54 (5.54)	25.79 (9.44)	33.21 (8.57)
9. FRAM	Males	1382	60.9 (9.5)	174.9 (6.8)	88.0 (15.1)	28.7 (4.5)	59.2 (6.6)	26.9 (3.8)	28.8 (9.6)	31.9 (5.6)
	Females	1574	60.9 (9.4)	161.2 (6.2)	71.8 (15.7)	27.6 (5.8)	40.1 (4.7)	17.7 (3.0)	31.7 (11.8)	42.9 (6.7)
	Combined	2956	60.9 (9.4)	167.6 (9.4)	79.4 (17.4)	28.1 (5.3)	49.0 (10.8)	22.0 (5.7)	30.3 (10.9)	37.7 (8.2)
10. GOOD	Males	940	18.9 (0.6)	181.7 (6.6)	73.9 (11.6)	22.4 (3.2)	57.6 (6.1)	26.9 (3.2)	13.2 (7.9)	17.1 (7.4)
11. HABC	Males	872	73.9 (2.9)	173.6 (6.4)	81.6 (12.4)	27.1 (3.7)	54.2 (6.6)	23.1 (3.5)	24.8 (6.9)	30.0 (4.7)
	Females	782	73.6 (2.8)	159.4 (5.8)	66.5 (12.1)	26.1 (4.5)	37.5 (5)	15.3 (2.4)	27.2 (7.9)	40.1 (5.5)
	Combined	1654	73.8 (2.8)	166.9 (9.3)	74.5 (14.4)	26.6 (4.1)	46.3 (10.2)	19.4 (4.9)	26.0 (7.5)	34.8 (7.2)
12. Indiana	Females	1467	32.7 (7.2)	165.3 (6.1)	71.8 (16.7)	26.3 (5.9)	41.1 (5.4)	NA	25.4 (12.1)	37.5 (9.3)
13. Kora F3	Males	771	62.49 (10.04)	173.55 (6.66)	84.86 (12.08)	28.17 (3.71)	60.62 (6.18)	25.66 (2.98)	24.25 (7.23)	28.06 (4.996)

Supplementary Table 5: Characteristics of the study populations from discovery and replication cohorts

Study	Gender	N with geno- and phenotype	Age (yrs) Mean (SD)	Height (cm) Mean (SD)	Weight (kg) Mean (SD)	BMI (kg/m2) Mean (SD)	Total lean mass (kg) mean (SD)	Appendicular lean mass (kg) mean (SD)	Total fat mass (kg) mean (SD)	Fat% mean (SD)
	Females	784	61.61 (9.89)	160.53 (6.23)	71.76 (13.14)	27.88 (5.04)	43.67 (5.4)	17.48 (2.54)	28.09 (8.49)	38.41 (5.16)
	Combined	1555	62.04 (9.97)	166.99 (9.16)	78.26 (14.22)	28.03 (4.43)	52.07 (10.27)	21.54 (4.93)	26.18 (8.12)	33.28 (7.25)
14. Kora F4	Males	874	54.13 (8.91)	174.38 (6.56)	86.47 (14.05)	28.36 (4.23)	60.75 (6.48)	25.78 (3.11)	24.29 (7.59)	28.03 (4.91)
	Females	920	53.58 (8.796)	161.32 (6.05)	72.6 (13.699)	27.91 (5.25)	44.10 (5.43)	17.76 (2.54)	27.34 (8.62)	37.47 (5.37)
	Combined	1794	53.85 (8.85)	167.68 (9.072)	79.36 (15.51)	28.13 (4.78)	52.21 (10.24)	21.67 (4.91)	25.85 (8.28)	32.87 (6.98)
15. Lolipop I	Males	613	53.9 (10.4)	175.0 (7.0)	85.8 (14.8)	28.0 (4.4)	62.1 (7.3)	NA	23.7 (9.9)	26.7 (7.1)
	Females	204	51.3 (10.4)	163.2 (6.5)	72.5 (15.5)	27.2 (5.7)	44.6 (5.4)	NA	28.0 (10.9)	37.3 (7.0)
	Combined	817	53.3 (10.5)	172.0 (8.5)	82.5 (16.0)	27.8 (4.7)	57.7 (10.3)	NA	24.8 (10.3)	29.3 (8.4)
16. Lolipop II	Males	625	56.2 (8.8)	175.1 (6.6)	87.7 (16.5)	28.6 (5.0)	61.9 (7.3)	NA	25.9 (12.3)	28.2 (8.4)
17. RS I	Males	1051	75.0 (5.7)	173.8 (6.6)	81.7 (11.6)	27.0 (3.4)	55.3 (5.8)	25.1 (3.1)	23.4 (7.6)	28.1 (6.2)
	Females	1384	75.4 (6.1)	160.5 (6.3)	71.2 (12.4)	27.6 (4.5)	40.3 (4.6)	17.5 (2.3)	28.4 (8.8)	39.0 (0.7)
	Combined	2435	75.2 (5.9)	166.3 (9.2)	75.7 (13.1)	27.4 (4.0)	46.8 (9.1)	20.8 (4.6)	26.2 (8.7)	34.3 (8.4)
18. RS II	Males	347	67.06 (6.4)	175.3 (6.1)	84.3 (10.8)	27.4 (3.1)	57.3 (5.8)	26.0 (3.2)	23.7 (7.3)	27.7 (5.9)
	Females	469	66.73 (6.2)	162.5 (6.1)	73.5 (12.8)	27.8 (4.5)	41.4 (4.7)	17.7 (2.2)	29.7 (9.4)	39.6 (6.6)
	Combined	816	66.87 (6.3)	168.0 (8.8)	78.1 (13.1)	27.7 (4.0)	48.2 (9.5)	21.2 (4.9)	27.2 (9.1)	34.5 (8.6)
19. TUK I	Females	1685	46.1 (12.0)	162.7 (6.1)	65.6 (12.4)	24.8 (4.6)	39.1 (5.3)	16.0 (2.9)	22.8 (8.9)	33.9 (7.9)
20. TUK II	Females	1679	47.7 (12.7)	162.5 (6.3)	66.3 (12.2)	25.1 (4.5)	39.0 (5.4)	15.8 (2.9)	23.8 (8.7)	35.1 (7.8)
Replication Cohorts										
1. AGES	Males	723	76.7 (5.5)	175.5 (6.1)	81.9 (13.3)	26.5 (3.8)	63.6 (7.6)	24.7 (3.3)	18.3 (7.0)	21.7(5.5)
	Females	874	76.3 (6.0)	160.7 (5.6)	70.0 (12.7)	27.1 (4.7)	45.8 (6.2)	16.6 (2.5)	24.2 (7.2)	34.0(4.7)
	Combined	1598	76.5 (5.6)	167.4 (9.4)	75.4 (14.3)	26.8 (4.3)	53.8 (11.2)	20.3 (5.0)	21.5 (7.7)	28.4(7.9)
2. Berlin Aging Study (BASE-II)	Males	202	58.07 (16.02)	176.31 (6.97)	82.30 (13.78)	26.48 (3.15)	55.13 (6.87)	24.57 (3.38)	23.27 (7.85)	28.08 (6.13)
	Females	422	57.89 (15.21)	163.76 (6.62)	68.79 (13.20)	25.70 (5.12)	39.03 (4.95)	16.40 (2.321)	26.36 (8.50)	38.19 (6.50)
	Combined	624	57.94 (15.47)	167.83 (8.94)	73.17 (14.80)	25.95 (4.84)	44.24 (9.42)	19.05 (4.68)	25.36 (8.42)	34.92 (7.94)
3. B-PROOF	Males	560	73.5 (5.6)	176.4 (6.4)	83.4 (11.2)	26.8 (3.2)	57.8 (7.2)	25.9 (3.3)	23.1 (7.2)	27.3 (5.9)
	Females	513	73.4 (5.8)	163.8 (6.2)	72.9 (12.3)	27.1 (4.3)	43.2 (5.6)	18.1 (2.4)	28.3 (9.0)	38.0 (6.7)
	Combined	1073	73.5 (5.7)	170.4 (8.9)	78.4 (12.9)	27.0 (3.8)	50.8 (9.8)	22.2 (4.9)	25.6 (8.5)	32.4 (8.3)
4. CAIFOS	Females	966	79.4 (2.9)	157.7 (6.0)	67.1 (12.1)	27.0 (4.8)	39.1 (4.8)	15.5 (2.4)	24.5 (7.3)	36.7 (5.9)
5. DOPS	Females	1717	50.6 (2.8)	164.5 (6.0)	67.7 (11.8)	25.0 (4.3)	42.5 (5.2)	18.1 (2.5)	19.6 (7.9)	28.1 (6.6)
6. FAMHS	Males	1048	55.76 (13.28)	176.33 (7.09)	91.08 (15.80)	29.28 (4.72)	61.10 (7.03)	26.11 (4.06)	29.98 (10.28)	32.11(6.05)
	Females	1299	56.57 (12.93)	162.37 (6.45)	74.77 (17.09)	28.35 (6.20)	41.29 (4.97)	20.31 (3.26)	33.48 (13.08)	43.34(7.47)

Supplementary Table 5: Characteristics of the study populations from discovery and replication cohorts

Study	Gender	N with geno- and phenotype	Age (yrs) Mean (SD)	Height (cm) Mean (SD)	Weight (kg) Mean (SD)	BMI (kg/m2) Mean (SD)	Total lean mass (kg) mean (SD)	Appendicular lean mass (kg) mean (SD)	Total fat mass (kg) mean (SD)	Fat% mean (SD)
	Combined	2347	56.21 (13.09)	168.60 (9.67)	82.05 (18.41)	28.77 (5.61)	50.14 (11.52)	22.90 (4.64)	38.32 (8.85)	38.32(8.85)
7. Genmets	Males	766	48.7 (10.2)	177.1 (6.3)	85.0 (13.1)	27.5 (3.9)	62.7 (7.9)	NA	19.9 (7.9)	22.6 (5.9)
	Females	791	51.9 (11.3)	164.0 (5.8)	70.9 (13.1)	27.3 (5.1)	45.1 (5.6)	NA	24.6 (9.6)	33.0 (7.1)
	Combined	1557	50.3 (10.9)	170.5 (8.9)	77.8 (14.9)	27.4 (4.5)	53.8 (11.1)	NA	22.3 (9.1)	27.9 (8.3)
8. Helsinki Birth Cohort	Males	702	61.35 (2.73)	176.83 (5.75)	86.05 (14.44)	27.56 (4.28)	61.64 (7.53)	NA	21.00 (8.76)	23.74 (5.99)
	Females	946	61.53 (3.03)	163.13 (5.71)	73.60 (13.88)	27.74 (5.07)	45.21 (5.50)	NA	25.74 (9.71)	33.96 (6.91)
	Combined	1648	61.45 (2.90)	168.97 (8.88)	78.91 (15.40)	27.66 (4.74)	52.21 (10.37)	NA	23.72 (9.60)	29.60 (8.26)
9. JoCo (Caucasian)	Males	326	65.12 (10.05)	172.83 (7.29)	90.90 (15.74)	30.37 (4.55)	65.11 (8.82)	29.65 (4.57)	26.02 (7.85)	28.08(4.86)
	Females	568	63.93 (10.18)	158.86 (5.92)	75.45 (17.73)	29.86 (6.69)	45.57 (7.37)	19.58 (3.67)	30.71 (10.82)	39.29(5.81)
	Combined	894	64.37 (10.14)	163.95 (9.32)	81.11 (18.58)	30.05 (6.00)	52.71 (12.31)	23.26 (6.30)	29.00 (10.09)	35.19(7.69)
10. JoCo (African-American)	Males	146	61.92 (10.00)	173.07 (7.17)	88.15 (18.02)	29.40 (5.48)	67.60 (11.03)	32.71 (5.98)	22.18 (8.93)	23.93(6.02)
	Females	234	61.61 (11.26)	159.93 (7.02)	85.01 (18.38)	33.25 (7.05)	51.10 (7.72)	23.39 (4.11)	34.97 (11.90)	39.67(6.08)
	Combined	380	61.73 (10.78)	164.98 (9.54)	86.22 (18.28)	31.77 (6.75)	57.44 (12.16)	26.97 (6.69)	30.05 (12.50)	33.62(9.77)
11. KoGES DXA	Males	976	64.04 (8.33)	165.49 (5.86)	64.92 (9.94)	23.66 (3.10)	49.03 (5.91)	21.31 (3.14)	13.48 (5.73)	20.05 (6.66)
	Females	1374	63.79 (8.55)	152.19 (6.03)	57.22 (8.77)	24.67 (3.31)	35.94 (3.92)	14.41 (2.05)	18.94 (6.06)	32.63 (6.59)
	Combined	2350	63.89 (8.46)	157.71 (8.86)	60.42 (10.02)	24.25 (3.26)	41.38 (8.07)	17.28 (4.26)	16.68 (6.50)	27.41 (9.07)
12. KoGES BIA	Males	2828	50.55 (8.56)	167.45 (5.84)	68.42 (9.72)	24.35 (2.88)	50.25 (6.14)	NA	15.33 (4.90)	21.98 (5.01)
	Females	2856	51.29 (8.94)	154.56 (5.39)	59.03 (8.48)	24.81 (3.25)	37.90 (4.33)	NA	19.20 (5.32)	31.81 (5.31)
	Combined	5684	50.92 (8.76)	160.98 (8.55)	63.84 (10.19)	24.58 (3.08)	44.04 (8.14)	NA	17.27 (5.47)	26.92 (7.13)
13 Kora F3	Males	650	50.2 (12.38)	176.18 (6.95)	85.99 (13.63)	27.68 (4)	62.22 (7.23)	26.57 (3.5)	23.76 (7.85)	27.04 (5.33)
	Females	731	50.81 (12.06)	162.53 (6.62)	69.45 (13.3)	26.34 (5.09)	43.76 (5.47)	17.68 (2.59)	25.69 (8.68)	36.14 (5.75)
	Combined	1381	50.53 (12.21)	168.96 (9.61)	77.24 (15.78)	26.97 (4.65)	52.45 (11.2)	21.87 (5.39)	24.79 (8.36)	31.86 (7.18)
14. Kora F4	Males	580	42.38 (15.66)	176.22 (7.18)	82.63 (11.65)	26.63 (3.58)	60.42 (6.26)	25.79 (3.06)	22.2 (6.92)	26.38 (5.17)
	Females	611	41.36 (14.76)	163.06 (6.87)	68.94 (13.45)	25.98 (5.11)	43.67 (5.47)	17.72 (2.58)	25.27 (8.83)	35.74 (5.93)
	Combined	1191	41.86 (15.21)	169.47 (9.62)	75.60 (14.34)	26.30 (4.44)	51.83 (10.23)	21.65 (4.92)	23.78 (8.1)	31.18 (7.28)
15. LOLIP-REP-IA610	Males	4332	56.82 (10.09)	169.81 (6.58)	78.24 (12.98)	27.10 (3.99)	56.00 (6.52)	NA	22.25 (9.00)	27.59 (7.14)
	Females	1022	56.88 (9.96)	155.52 (6.02)	69.46 (12.72)	28.77 (5.31)	41.62 (4.68)	NA	27.84 (9.10)	39.14 (6.39)
	Combined	5354	56.83 (10.06)	167.09 (8.58)	76.57 (13.38)	27.41 (4.32)	53.25 (8.40)	NA	23.31 (9.28)	29.79 (8.35)
16. LOLIP-REP-IA_I	Males	1929	48.03 (10.52)	171.05 (6.72)	78.68 (14.19)	26.84 (4.25)	56.49 (6.75)	NA	22.19 (9.95)	27.15 (7.69)
17. LOLIP-REP-IA_P	Males	551	53.44 (8.05)	170.16 (6.35)	79.5 (13.60)	27.42 (4.21)	56.99 (6.49)	NA	22.52 (9.44)	27.40 (7.09)

Supplementary Table 5: Characteristics of the study populations from discovery and replication cohorts

Study	Gender	N with geno- and phenotype	Age (yrs) Mean (SD)	Height (cm) Mean (SD)	Weight (kg) Mean (SD)	BMI (kg/m2) Mean (SD)	Total lean mass (kg) mean (SD)	Appendicular lean mass (kg) mean (SD)	Total fat mass (kg) mean (SD)	Fat% mean (SD)
18. METSIM	Males	8115	57.51 (7.01)	175.87 (6.31)	84.38 (13.95)	27.26 (4.15)	63.59 (8.88)	25.42 (3.21)	20.79 (8.04)	24.11 (6.52)
19. MrOS Sweden	Males	2764	75.44 (3.15)	174.81 (6.51)	80.48 (11.71)	26.32 (3.45)	55.48 (6.78)	24.23(3.19)	22.02 (7.63)	26.80 (6.60)
20. MrOS U.S.	Males	4549	73.97 (5.95)	174.49 (6.62)	83.48 (13.04)	27.39 (3.82)	57.10 (7.05)	24.28 (3.39)	21.92 (7.14)	26.31 (5.40)
21. OPRA	Females	902	75.23 (0.15)	160.52.5.71)	67.78 (11.68)	26.27 (4.19)	37.28 (3.95)	15.99 (2.03)	26,09 (7,90)	38.23 (6.88)
22. ORCADES	Males	241	56.97 (13.99)	171.6 (6.47)	85.24 (13.52)	27.92 (4.14)	61.15 (7.14)	26.08 (3.47)	21.15 (8.39)	24.19 (5.94)
	Females	311	57.69 (13.31)	160.5 (6.19)	71.15 (13.59)	27.57 (5.19)	42.27 (5.6)	16.79 (2.57)	26.78 (9.12)	36.84 (6.51)
	Combined	552	57.38 (13.6)	166.6 (9.42)	77.29 (15.25)	27.72 (4.76)	50.51 (11.30)	20.84 (5.50)	24.32 (9.23)	56.97 (8.86)
23. PEAK 25	Females	1001	25.49 (0.21)	167.56 (6.08)	64.71 (11.39)	23.04 (3.81)	40.38 (4.68)	17.49 (2.33)	21.22 (8.40)	33.01 (12.01)
24. PIVUS	Males	419	71.92 (0.83)	175.52 (6.34)	82.94 (12.47)	26.91 (3.65)	56.22 (6.32)	24.69 (3.04)	23.49 (8.14)	27.67 (6.44)
	Females	437	72.14 (0.89)	161.74 (5.76)	69.92 (12.71)	26.74 (4.78)	39.64 (4.99)	16.58 (2.45)	27.63 (9.27)	38.54 (7.31)
	Combined	856	72.03 (0.87)	168.47 (9.17)	76.28 (14.17)	26.82 (4.26)	47.74 (10.05)	20.54 (4.90)	25.61 (8.97)	33.23 (8.78)
25. RISC	Males	459	43.30 (8.53)	178.41 (6.92)	83.95 (12.92)	26.34 (3.53)	64.85 (7.22)	NA	19.10 (8.16)	22.05 (6.61)
	Females	586	44.43 (8.19)	164.93 (6.32)	67.44 (11.96)	24.80 (4.24)	44.85 (4.22)	NA	22.57 (8.94)	32.35 (7.51)
	Combined	1045	43.93 (8.36)	170.84 (9.39)	74.68 (14.85)	25.48 (4.02)	53.64 (11.47)	NA	21.04 (8.77)	27.83 (8.77)
26. Rotterdam III	Males	680	56.01 (5.51)	178.9 (6.65)	88.26 (12.92)	27.58 (3.76)	59.72 (6.07)	27.05 (3.29)	25.167 (9.16)	27.83 (0.067)
	Females	914	56.32 (5.96)	164.9 (6.06)	73.85 (13.28)	26.31 (4.71)	41.72 (4.57)	17.94 (2.32)	29.530 (10.36)	38.93 (7.51)
	Combined	1594	56.12 (5.77)	1.709 (0.09)	80.00 (14.93)	27.34 (4.33)	49.40 (10.34)	21.83 (5.29)	27.667 (10.09)	34.20 (9.04)
27. Rush Memory and Aging Project	Males	152	83.13 (6.02)	174.59 (7.15)	81.56 (12.98)	26.74 (3.89)	55.49 (8.89)	NA	26.08 (8.29)	31.62 (7.20)
	Females	465	83.01 (6.62)	158.68 (6.51)	67.28 (13.78)	26.75 (5.40)	40.47 (5.50)	NA	26.81 (9.62)	38.86 (6.59)
	Combined	617	83.04 (6.47)	162.60 (9.57)	70.80 (14.91)	26.75 (5.06)	44.17 (9.17)	NA	26.63 (9.31)	37.08 (7.43)
28. SHIP 2	Males	710	59.12 (13.51)	174.77 (6.81)	88.12 (14.15)	28.81 (4.07)	66.25 (8.82)	26.40 (3.69)	21.89 (7.26)	24.38 (5.14)
	Females	838	56.92 (13.32)	162.64 (6.81)	73.73 (14.15)	27.92 (5.33)	47.62 (5.50)	18.16 (2.81)	26.11 (9.60)	34.38 (6.48)
	Combined	1548	57.93 (13.44)	168.21 (9.10)	80.33 (15.86)	28.33 (4.81)	56.16 (11.76)	21.94 (5.23)	24.18 (8.86)	29.79 (7.72)
29. SHIP TREND	Males	428	50.67 (14.27)	176.51 (6.87)	86.93 (12.97)	27.88 (3.71)	66.14 (8.06)	26.70 (3.33)	20.82 (6.89)	23.49 (5.31)
	Females	530	50.05 (13.09)	164.37 (6.32)	72.51 (13.50)	26.87 (4.94)	47.33 (5.26)	18.28 (2.61)	25.16 (9.26)	33.71 (6.44)
	Combined	958	50.32 (13.62)	169.79 (8.92)	78.96 (15.07)	27.32 (4.46)	55.73 (11.48)	22.04 (5.12)	23.22 (8.56)	29.15 (7.83)
30. SOF	Females	3365	73.56 (5.31)	159.29 (5.78)	67.65 (11.72)	26.61 (4.36)	39.96 (4.36)	17.08 (2.33)	27.14 (8.60)	39.51 (5.69)
31. ULSAM	Males	496	81.67 (0.93)	172.97 (5.51)	77.85 (10.83)	26.01 (3.33)	51.93 (5.55)	22.19 (2.82)	22.47 (7.63)	28.21 (6.92)
32. VIS	Males	366	55.52 (14.74)	175.8 (7.28)	85.06(13.12)	27.48 (3.72)	62.84(7.32)	26.85 (3.54)	22.42 (7.32)	25.758 (5.23)

Supplementary Table 5: Characteristics of the study populations from discovery and replication cohorts

Study	Gender	N with geno- and phenotype	Age (yrs) Mean (SD)	Height (cm) Mean (SD)	Weight (kg) Mean (SD)	BMI (kg/m2) Mean (SD)	Total lean mass (kg) mean (SD)	Appendicular lean mass (kg) mean (SD)	Total fat mass (kg) mean (SD)	Fat% mean (SD)
	Females	503	55.97 (15.86)	161.7 (6.71)	70.79\(12.44)	27.10 (4.63)	44.9(5.84)	18.16 (2.77)	25.976 (8.30)	35.95 (6.30)
	Combined	869	55.78 (15.39)	167.7 (9.85)	76.8(14.55)	27.26 (4.27)	52.45(10.99)	21.82 (5.31)	24.484 (8.09)	31.672 (7.74)
33. WHI	Females	3000	64.00 (7.4)	161.10 (6.6)	70.50 (15.7)	27.10 (5.8)	37.00 (5.1)	14.10 (3.3)	29.8 (11.2)	42.1 (7.7)

SUPPLEMENTARY TABLE 6: Quality control procedures and exclusion criteria for individuals of the GWA studies, SNP selection criteria and genotype imputation parameters used in the GWA discovery studies

	QC and exclusion criteria of individuals		Genotyping			SNP Inclusion Criteria				SNP Imputation			Association	
**Study**	**Call rate* selection**	**Sample QC / Other exclusions**	**Platform(s)/Chip(s)**	**Calling Algorithm**	**Genotyping Facility**	**MAF**	**Call Rate**	**P-test HWE**	**Included SNPs**	**Method**	**MAF**	**Quality Metric**	**Included SNPs**	**Software**
**AGES**	>= 97 %	1. gender mismatch;<br>2. samples where genotypes mismatch fingerprint genotypes	Illumina Human 370 CNV	Beadstudio Genecall	National Institute on Aging, Bethesda, MD	≥ 1%	≥ 95%	$> 10^{-6}$	308340	MACH	≥ 1%	(O/E)σ^2 ratio ≥ 0.1	2408992	ProbABEL
**Amish**	95%	1. Missing lean mass data for whole body and/or appendicular measures<br>2. Missing covariate data	Affymetrix / either 500k or 6.0	Birdseed	UMB Genomics Core Facility, Baltimore MD 21201	1%	95%	$\geq 10^{-6}$	338598	MACH	≥ 1%	(O/E)σ^2 ratio ≥ 0.1	2404474	MMAP - (Mixed Model Analysis for Pedigrees; author: J O'Connell)
**CHS**	>95%	1. excluded African-Americans<br>2. Samples were also excluded for sex mismatch, discordance with prior genotyping	Illumina 370CNV BeadChip	BeadStudio	General Clinical Research Center's Phenotyping/Genotyping Laboratory at Cedars-Sinai	NA<br><br>SNPs were also excluded if > 2 duplicate errors or Mendelian inconsistencies (for reference CEPH trios), heterozygote frequency = 0, SNP not found in HapMap	>97%	$> 10^{-5}$	306655	BimBAM		observed/expected variance<br><br>**Betas/SEs were set to missing for SNPs with a dosage variance <0.01	*depends upon the analysis	R
**CoLaus**	≥70%	1. ethnic outliers<br>2. related individuals<br>3. missing lean mass data	Affymetrix / 500K	BRLMM	Expression Analysis / Affymetrix	≥ 1%	≥ 90%	$\geq 10^{-7}$	390631	IMPUTE		r-square-hat	2557249	QUICKTEST
**deCODE**	≥0.91%	1. missing BMD measurements	Illumina HH300 and 370CNV	BeadStudio	deCODE genetics	> 1%	> 96%	$> 10^{-6}$	281410	IMPUTE		properinfo > 0.4	2454808	SNPTEST
**EPIC**	≥ 90%	1. heterozygosity <23% or >30%;<br>2. >5.0% discordance in SNP pairs with r2=1 in HapMap;<br>3. ethnic outliers;<br>4. related individuals and duplicates	Affymetrix 500K Array Set	BRLMM	The Wellcome Trust Sanger Institute, Hinxton, Cambridge, UK	≥ 1%	≥ 90%	$> 10^{-6}$	382036	IMPUTE	≥ 1%	Prop.Inf ≥ 0.4	2357235	SNPTEST
**ERF**	≥ 95%	1. missing phenotypes<br>2. gender mismatches<br>3. heterozygosity: false discovery rate used	Illumina 6K, Illumina 318K, Illumina 370K, Affymetrix 250K	Beadstudio Genecall	Erasmus MC Rotterdam	≥ 1%	≥ 95%	$\geq 10^{-6}$	305093	MACH	≥ 1%	(O/E)σ2 ratio ≥ 0.1	2443824	ProABEL R-Package
**Fenland**	≥ 90%	1. failed heterozygosity check: upperbound 0.2882 lowerbound 0.2735<br>2. failed relatedness check (sample with lower call rate in related samples)<br>3. failed duplicate check (sample with lower call rate in duplicates)	Affymetrix SNP5.0 chip	BRLMM	The Wellcome Trust Sanger Institute, Hinxton, Cambridge, UK	≥ 1%	≥ 90%	$> 10^{-6}$	362055	IMPUTE	≥ 1%	Prop.Inf ≥ 0.4	2551274	SNPTEST
**GOOD**	≥ 97.5%	1. excess of autosomal heterozygosity > 0.33 ~ FDR < 0.1%;<br>2. ethnic outliers using IBS distances > 3SD;<br>3. duplicates and/or first degree relatives using IBS probabilities > 97%.	Illumina / HumanHap 610K	Beadstudio Genecall	Genetic Laboratory, Department of Internal Medicine, Erasmus MC, Rotterdam, The Netherlands	≥ 1%	≥ 98%	$\geq 10^{-6}$	521160	MACH	≥ 1%	(O/E)σ2 ratio ≥ 0.1	2543887	MACH2QTL

SUPPLEMENTARY TABLE 6: Quality control procedures and exclusion criteria for individuals of the GWA studies, SNP selection criteria and genotype imputation parameters used in the GWA discovery studies

	QC and exclusion criteria of individuals		Genotyping			SNP Inclusion Criteria				SNP Imputation			Association	
**Study**	**Call rate* selection**	**Sample QC / Other exclusions**	**Platform(s)/Chip(s)**	**Calling Algorithm**	**Genotyping Facility**	**MAF**	**Call Rate**	**P-test HWE**	**Included SNPs**	**Method**	**MAF**	**Quality Metric**	**Included SNPs**	**Software**
**FRAM**	≥ 97.0%	1. autosomal heterozygosity <0.33 or > 0.37<br>2. ethnic outliers (using Eigenstraat)<br>3. missing BMD or weight measurements	Affymetrix 500K Dual GeneChip<br>+50K gene-centered MIP set	BRLMM	Affymetrix Inc.<br>San Francisco, CA, USA	≥ 1%	≥ 97%	$\geq 10^{-6}$	378163	MACH	≥ 1%	(O/E)σ2 ratio ≥ 0.1	2471285	Kinship R-Package
**HABC**	≥ 97 %	1. ethnic outliers (using Eigenstraat)<br>2. missing BMD or weight measurements	Illumina Human1M-Duo	Beadstudio	CIDR	≥ 1%	≥97%	$\geq 10^{-6}$	914263	MACH	>= 1%	(O/E)σ2 ratio ≥ 0.1	2543887	R
**Indiana**	≥ 95%	1. call rates, 2. missing phenotypes	Illumina Human610 Quad V1	Beadstudio	CIDR	≥ 1%	≥ 90%	$\geq 10^{-4}$	553331	IMPUTE	≥ 1%	Threshold for calling genotypes >=0.9	2293182	Merlin
**Kora F3**	≥0,93	different exclusions (e.g. gender mismatch), phenotype exclusions	Affymetrix 500K	BRLMM	Helmholtz Center, Munich	no	0.9	no	490032	MACH		r2_hat		mach2qtl
										WBLM	≥ 1%		2470527	
										ALM	no		2557252	
**Kora F4**	≥0,93	different exclusions (e.g. gender mismatch), phenotype exclusions	Affymetrix 6.0 1000K	Birdseed2	Helmholtz Center, Munich	no	no	no	909622	Impute		Proper_info		SNPTEST
										WBLM	≥ 1%		1912063	
										ALM	no		2099822	
**Lolipop**														
**Lolipop Affymetrix**	≥ 95%	1. duplicates.<br>2. missing phenotypes.<br>3. call rates.	Affymetrix / GeneChip Human Mapping 500K Array	BRLMM	Genotyped by GSK	≥ 1%	≥ 98%	$\geq 10^{-6}$	374773	MACH	≥ 1%	r2_hat	2557252	MACH2QTL
**Lolipop Perlegen**	≥ 95%	1. duplicates (some already genotyped in Affymetrix data).<br>2. missing phenotypes.<br>3. call rates.	Perlegen / custom	Perlegen	Genotyped by Perlegen	≥ 1%	≥ 90%	$\geq 10^{-6}$	~200K	MACH	≥ 1%	r2_hat	2557252	MACH2QTL
**RS I**	≥ 97.5%	1. gender mismatch with typed X-linked markers<br>2. excess autosomal heterozygosity > 0.336~FDR>0.1%<br>3. duplicates and/or	Illumina / HumanHap 550 V.3	Beadstudio Genecall	Genetic Laboratory Dept Internal Medicine Erasmus MC, The Netherlands	≥ 1%	≥ 98%	$\geq 10^{-6}$	512349	MACH	≥ 1%	(O/E)σ2 ratio ≥ 0.1	2467002	MACH2QTL
**RS II**	≥ 97.5%	1. gender mismatch with typed X-linked markers<br>2. excess autosomal heterozygosity F-value < 0.055 (4SD)	Illumina / HumanHap 550 V.3	Beadstudio Genecall	Genetic Laboratory Dept Internal Medicine Erasmus MC, The Netherlands	≥ 1%	≥ 98%	$\geq 10^{-6}$	512349	MACH	≥ 1%	O/E)σ2 ratio ≥ 0.1	2467002	MACH2QTL
**TUK**	> 98%	1. autosomal heterozygosity <0.33 or > 0.37<br>2.ethnic outliers (using PLINK)<br>3. missing measurements<br>4. mismatch with Sequenom typing	Illumina / HumanHap 300 & 550	Illuminus	Wellcome Trust Sanger institute	≥ 1%	≥ 95%	$\geq 10^{-6}$	307040	IMPUTE	≥ 1%	Prop.Inf ≥ 0.4	2288471	GenABEL R-Package

*Sample genotyping success rate; i.e. percentage of succesfully genotyped markers per sample

Supplementary Table 7: Quality control procedures and exclusion criteria for individuals of the GWA studies, SNP selection criteria and genotype imputation parameters used in the GWA replication studies

	QC and exclusion criteria of individuals		Genotyping			SNP Inclusion Criteria				SNP Imputation			Association	
Study	Call rate* selection	Sample QC / Other Exclusions	Platform(s)/Chip(s)	Calling Algorithm	Genotyping Facility	MAF	Call Rate	P-test HWE	Included SNPs	Method	MAF	Quality Metric	Included SNPs	Software
**BASE-II**	≥ 95%	excluded were: duplicated samples, samples with sex inconsistencies, related individuals, samples with excessive heterozygosity, population outliers	Affymetrix 6.0	Birdsuite v2	ATLAS Biolabs, Inc, Germany	≥ 1%	≥ 98%	$\geq 10^{-6}$	710205	IMPUTE v2	≥ 1%	NA	32	SNPTEST v2
**B-PROOF**	≥97.5%	1. ethnic outliers<br>2. related individuals<br>3. gender mismatch<br>4. excess autosomal heterozygosity F-value < -0.05207 (4SD)	Illumina Omni Express	Beadstudio Genecall	Genetic Laboratory, Department of Internal Medicine, Erasmus MC, The Netherlands	≥1%	≥98%	$\geq 10^{-6}$	722053	MACH	≥ 1%	r2_hat	2543887	MACH2QTL in GRIMP
**FamHS**	≥ 98%	1. Missing trait measures:<br>2. Not Caucasian origin:	ILLUMINA 550K, ILLUMINA 610K, and ILLUMINA 1M	BEADSTUDIO-Genecall v3.0	Washington University (St. Louis, MO, USA)	≥1%	≥98%	$> 10^{-6}$	493938	MACH	All	All	2543887	R version 2.14.1
**Genmets controls**	>95%	1. heterozygosity check<br>2. gender check<br>3. cryptic relatedness<br>4. missing phenotypes	Ilumina 610K	Illuminus	Wellcome Trust Sanger Institute, Hinxton, UK	≥ 1%	≥ 95%	$\geq 10^{-6}$	598203	MACH 1.0.10	≥ 1%	R2	2543888	probABEL, R
**Helsinki Birth Cohort**	>95%	1. missing phenotype or covariates<br>2. gender mismatches	modified Illumina Infinum 610K Quad	BeadStudio	The Wellcome Trust Sanger Institute, Hinxton, Cambridge, UK	> 1%	>95%	$> 10^{-6}$	2543888	MACH	≥ 1%	r2hat ≥ 0.3	2430098	ProbABEL
**Johnson County Study**	>98%	1. sex/gender mismatch<br>2. First-degree relative<br>3. disagreement between reported and genotypic race<br>4. missing any covariate	Illumina Infinium 1M-Duo bead arrays	Beadstudio	Expression Analysis Inc	≥ 0.5%	≥ 98%	$> 10^{-4}$	1065734	MACH	≥ 5%	rsq>0.3	2176145	R and ProbABEL
**KoGES**	≥ 95%	gender mismatch with typed X-linked markers	Affymetrix Genechip 5.0	Birdseed-v2	DNALink, Korea	≥ 1%	≥ 99%	$\geq 10^{-3}$	30	IMPUTE v1.0.0	≥ 1%	NA	33	SAS
**Kora F3**	none	None	Illumina CardioMetabochip	Genome Studio	Helmholtz Zentrum München	None	none	None	185781	NA	NA	NA	3	R
**Kora F4**	none	None	Illumina CardioMetabochip	Genome Studio	Helmholtz Zentrum München	None	none	None	185781	NA	NA	NA	3	R
**LOLIP-REP-IA610**	≥ 95%	1. duplicates (for quality check)<br>2. missing phenotypes<br>3. call rates<br>4. Relateds<br>5. Wrong gender<br>6. Ethnic outliers	Illumina Human610	BeadStudio	Genotyped by DeCode	≥ 1%	≥ 90%	$\geq 10^{-6}$	544390	MACH	≥ 1%	r2_hat	2177742	MACH2QTL

Supplementary Table 7: Quality control procedures and exclusion criteria for individuals of the GWA studies, SNP selection criteria and genotype imputation parameters used in the GWA replication studies

	QC and exclusion criteria of individuals		Genotyping			SNP Inclusion Criteria				SNP Imputation			Association	
**Study**	**Call rate* selection**	**Sample QC / Other Exclusions**	**Platform(s)/Chip(s)**	**Calling Algorithm**	**Genotyping Facility**	**MAF**	**Call Rate**	**P-test HWE**	**Included SNPs**	**Method**	**MAF**	**Quality Metric**	**Included SNPs**	**Software**
**LOLIP-REP-IA_I**	≥ 95%	1. duplicates (some already genotyped in IA610)<br>2. missing phenotypes<br>3. call rates	Affymetrix / GeneChip Human Mapping 500K Array	BRLMM	Genotyped by GSK	≥ 1%	≥ 90%	$\geq 10^{-6}$	245892	MACH	≥ 1%	r2_hat	1958375	RMACH2QTL
**LOLIP-REP-IA_P**	≥ 95%	1. duplicates (some already genotyped in IA610)<br>2. missing phenotypes<br>3. call rates	Perlegen / custom	Perlegen	Genotyped by Perlegen	≥ 1%	≥ 90%	$\geq 10^{-6}$	184469	MACH	≥ 1%	r2_hat	1958375	MACH2QTL
**MrOS Sweden (Gothenburg)**	≥ 98%	exclusion based on IBD clustering, Identical twins, sample duplicate	Illumina HumanOmni1_Quad_v1-0 B	Illumina GenomeStudio	Broad Institute	≥ 1%	≥95%	$\geq 10^{-4}$	739477	Minimach	≥ 1%	R2	3020488	MACH2QTL, PLINK
**MrOS US**	≥97%	Duplicate, sex mismatch and relatedness and non-Caucasian	Illumina Human Omni1-Quad	Beadstudio	Broad institute (Boston, MA)	≥ 1%	≥95%	$\geq 10^{-4}$	725550	MINIMAC	≥ 1%	R2 Mach	2391250	R
**ORCADES**	≥ 97%	1. Missing trait measures:	Illumina HumanHap300v2	Beadstudio	Helmholtz Zentrum München, Neuherberg, Germany	≥ 1%	≥ 98%	$\geq 10^{-6}$	285491	MACH	≥ 1%	R2	2543887	probABEL
**RISC**		1. sex-mismatch<br>2. non-Caucasian<br>3. Missing trait measures	KASPar Chemistry	KASPar	Kbioscience	≥ 1%	≥ 95%	$\geq 10^{-6}$		MACH	≥ 1%	r2 ≥ 0.3	2451091	MACH2QTL
**RS III**	≥ 97.5%	1. gender mismatch with typed X-linked markers<br>2. excess autosomal heterozygosity > 0.336~FDR>0.1%<br>3. duplicates and/or first degree relatives IBD piHAT>40% from Plink	Illumina / HumanHap 550 V.3	Beadstudio Genecall	Genetic Laboratory Dept Internal Medicine Erasmus MC, The Netherlands	≥ 1%	≥ 98%	$\geq 10^{-6}$	512349	MACH	≥ 1%	(O/E)σ2 ratio ≥ 0.1	2467002	MACH2QTL
**Rush Memory and Aging Project**	≥ 95%	1. sex discordance with reported<br>2. inbreeding coefficient F>0.04	Affymetrix Genechip 6.0	Birdsuite, Broad Institute	Broad Institute's Center for Genotyping; Translational Genomics Research Institute	≥ 1%	≥ 95%	$\geq 10^{-6}$	645349	MACH	≥ 1%	R2	2543887	probABEL, R
**SHIP 2**	> 92%	gender mismatch, duplicate samples by IBD estimation	Affymetrix Genechip 6.0	Birdseed2	Affymetrix Inc., Greifswald University	NA	NA	NA	869224	IMPUTE	NA	NA	2748910	Stata
**SHIP TREND**	≥ 94%	gender mismatch, duplicate samples by IBD estimation	Illumina Omni 2.5	Beadstudio Genecall	Helmholtz Zentrum München, Greifswald University	NA	> 90%	$\geq 10^{-4}$	1782967	IMPUTE	NA	NA	3437411	Stata
**SOF**	≥97%	Duplicate, sex mismatch and relatedness and non-Caucasian	Illumina Human Omni1-Quad	Beadstudio	Broad institute (Boston, MA)	≥ 1%	>=95%	$\geq 10^{-4}$	725550	MINIMAC	≥ 1%	R2 Mach	2391250	R
**VIS**	≥ 95%	1. Missing trait measures:	Illumina HumanHap300v1	Beadstudio	Wellcome Trust Clinical Research Facility, Edinburgh	≥ 1%	≥ 98%	$\geq 10^{-6}$	289287	MACH	≥ 1%	R2	2543887	probABEL

*Sample genotyping success rate; i.e. percentage of successfully genotyped markers per sample

Supplementary Table 8: Replication cohorts de novo genotyping information

Study	N (samples)	Genotyping Technique	Genotyping faclility	% samples in duplicate	call rate (%)	HWE cut-off	Statistical Analysis Program
**AGES**	1598	KBioScience Allele-Specific Polymorphism (KASP) SNP genotyping system	KBioscience, UK	3%	> 90%	1.00E-06	R
**CAIFOS**	966	KBioScience Allele-Specific Polymorphism (KASP) SNP genotyping system	KBioscience, UK	3%	98	< 0.00147	SPSS
**DOPS**	1717	KBioScience Allele-Specific Polymorphism (KASP) SNP genotyping system	KBioscience, UK	3%	> 90%	< 0.00147	SPSS
**METSIM**	8115	Sequenom, TaqMan	Kuopio	3%	99	0.0016	SPSS
**OPRA**	1008	KBioScience Allele-Specific Polymorphism (KASP) SNP genotyping system	KBioscience, UK	3%	>97%	$p<0.05$	SPSS v18
**PEAK 25**	1005	KBioScience Allele-Specific Polymorphism (KASP) SNP genotyping system	KBioscience, UK	3%	>97%	$p<0.05$	SPSS v18
**PIVUS***	949	Illumina Golden Gate	Uppsala SNP&SEQ Technology Platform,	0	≥99% (SNPs with MAF<5%) or ≥95% (SNPs with MAF≥5%)	1.00E-06	PLINK
**ULSAM***	1179	Illumina Golden Gate	Uppsala SNP&SEQ Technology Platform	0	≥99% (SNPs with MAF<5%) or ≥95% (SNPs with MAF≥5%)	1.00E-06	PLINK
**WHI**	2406 non-Hispanic Caucasian, 354 Hispanic	Sequenom's iPLEX	Michael B. Seddon, Director of Operation, BioServe Biotechnologies, Ltd., 9000 Virginia Manor Road, Suite 207, Beltsville, MD 20705	3%	75-97%	0.01	SAS,STATA and PLINK

*some SNPs were replicated using GWAS with Omni Express, and Metabochip and Omni 2.5

Supplementary Table 9: eQTLs in human muscle tissue, liver, and fat for 5 GWAS loci and *IRX3* and *IRX5*

				**Muscle**					**Liver***	**Omental fat†**	**Subcutaneous fat†**
**SNP ID**	**Chr.**	**Position**	**Gene Symbol of Expressed Gene**	**GTEx n=361**	**Fusion N=271**	**Pima N=225**	**Chest Wall Muscle n=75**	**STRRIDE n=57**	**n=956**	**n=742**	**n=610**
rs2943656	2	226830162	*IRS1*	8.1×10^{-3}	0.85	NA	>0.1	0.04	0.04	4×10^{-7}	6.44×10^{-6}
rs9991501	4	88477507	*HSD17B11*	1.4×10^{-4}	0.05	NA	NA	NA	>0.1	>0.1	>0.1
rs2287926	5	82851164	*VCAN*	0.59	0.04	0.09	>0.1	0.35	>0.1	>0.1	>0.1
rs4842924	15	84587607	*ADAMTSL3*	0.22	0.83	0.21	>0.1	0.44	>0.1	>0.1	>0.1
rs9936385	16	52376670	*FTO*	2.9×10^{-3}	4.4×10^{-11}‡	0.69	>0.1	0.53	>0.1	>0.1	>0.1
rs9936385	16	52376670	*IRX3*	0.78	0.44	0.47	NA	0.35	NA	NA	NA
rs9936385	16	52376670	*IRX5*	0.70	0.03	0.26	NA	0.55	NA	NA	NA

NA: eQTL analyses were not available due to low minor allele frequencies for rs2943656 and rs9991501. For *IRX3* and *IRX5*, data was only accessible to us for GTEx, FUSION and the Pima Indian studies.

* Liver samples acquired from Caucasian individuals from three independent liver collections at tissue resource centers at Vanderbilt University, the University of Pittsburgh, and Merck Research Laboratories[1]

† Fat tissue collected from patients at the time of Roux-en Y Gastric Bypass surgery at Massachusetts General Hospital between 2000 and 2007[2]

‡ p= 0.16 following conditional analysis as described in the supplemental methods section 2.4

Supplementary Table 10: Association* between the five replicated SNPs and other reported anthropometric phenotypes.

			Hip Circumference		Waist circumference		Waist-Hip Ratio		BMI		Height	
SNP	Genes	Alleles	Beta	P-values	Beta	P-values	Beta	P-values	Beta	P-values	Beta	P-values
rs2943656	*IRSI*	A/<u>**G**</u>	0.017	$4.00x10^{-6}$	0.015	$2.10x10^{-5}$	0.007	0.041	0.0135	$1.55x10^{-5}$	-0.0025	0.4
rs9991501	*HSD17B11*	T/<u>**C**</u>	0.0042	0.8	-0.0002	0.99	0.001	0.95	$3.00x10^{-4}$	0.981	-0.0071	0.46
rs2287926	*VCAN*	<u>**A**</u>/G	-0.0008	0.91	-0.0067	0.31	-0.014	0.029	0.0016	0.7827	-0.0034	0.45
rs4842924	*ADAMTSL3*	T/<u>**C**</u>	0.018	$2.50x10^{-7}$	0.017	$1.00x10^{-6}$	0.004	0.22	-0.0042	0.1732	0.044	$6.10x10^{-52}$
rs9936385	*FTO*	T/<u>**C**</u>	-0.07	$9.30x10^{-81}$	-0.072	$1.30x10^{-96}$	-0.042	$3.10\ x10^{-35}$	-0.0795	$2.73x10^{-144}$	0.0096	0.0015

			Hip Circumference (BMI adjusted)		Waist Circumference (BMI adjusted)		Waist to Hip Ratio (BMI adjusted)	
SNP RS#	Genes	Alleles	Beta	P-values	Beta	P-values	Beta	P-values
rs2943656	*IRSI*	A/<u>**G**</u>	0.0071	0.053	0.005	0.15	-0.0001	0.98
rs9991501	*HSD17B11*	T/<u>**C**</u>	-0.017	0.27	-0.01	0.49	-0.0063	0.68
rs2287926	*VCAN*	<u>**A**</u>/G	-0.0072	0.29	-0.024	0.00022	-0.021	0.0017
rs4842924	*ADAMTSL3*	T/<u>**C**</u>	0.037	$7.20x10^{-26}$	0.035	$2.70x10^{-26}$	0.0064	0.058
rs9936385	*FTO*	T/<u>**C**</u>	-0.0029	0.43	-0.0078	0.025	-0.0035	0.32

*All beta coefficients are based on inverse standard normalized residuals (adjusted for covariates)

Lean mass decreasing allele is reported in bold and underlined (eg. <u>**G**</u>)

Anthropometric associations where the lean mass lowering allele is associated with higher anthropometric trait values are shown in red. Anthropometric associations where the lean mass lowering allele is associated with lower anthropometric trait values are shown in blue.

Supplementary Notes

1. <u>De novo genotyping stage 2:</u>

A total of 18,943 samples from 9 studies with lean mass information was de-novo genotyped in four main genotyping centers (KBioSciences), Uppsala SNP & Seq Technology Platform (PIVUS and ULSAM), Kuopio (METSIM), and Women's Health Initiative (BioServe Biotechnologies, Ltd.). (Supplemental Table 9).

1.1. KBioSciences

The majority of the studies participating in the replication stage had genotyping performed by K-Biosciences. All SNPs genotyped by K-Biosciences (www.kbioscience.co.uk) used a competitive allele specific PCR (KASPar) assay. A Y-chromosome specific assay was evaluated in all samples. We performed a standardized quality control for the K-Bioscience genotyping including: 1) visual checking of the genotype clustering of the results; 2) exclusion of samples with >20% of genotypes missing (no genotype for 7 or more of the SNPs) - 82 samples (4.6%); 3) exclusion of SNPs with a call rate <90% - 0 SNPs; 4) HWE check to retain only SNPs with a p-value > 0.00147 - all SNPs.

1.2. Uppsala SNP and Seq Technology Platform - ULSAM/PIVUS

The ULSAM and PIVUS samples (n=2,213) were genotyped for 34 SNPs using the Illumina Golden Gate assay.[3] Allele signal intensities were read-out by the Illumina BeadXpress system and converted into genotypes using the software

GenomeStudio 2010.3 (Illumina Inc.). The 34 SNPs had sample call rate from 96.2 to 99.2%. Quality control was achieved by typing approximately 60 samples in duplicate (2.4% of the total number of genotypes). The reproducibility was determined to be 100% (0 duplicate errors in 2336 duplicate tests). An inheritance test was performed by adding DNA samples from 15 individuals from CEPH/Utah Pedigree 1362 from Coriell's Polymorphism Discovery Resource. The inheritance was determined to be 100% correct (0 inheritance errors in 656 inheritance tests). All 34 SNPs were also included for genotyping in the HapMap project in six of the individuals from CEPH/Utah Pedigree 1362. The concordance to the HapMap data was determined to be 100% (0 conflicts in 256 concordance tests).

1.3. Kupio Sequenom – METSIM

Genotyping of SNPs was performed using the Sequenom iPlex Gold SBE assay at the University of Eastern Finland. The Sequenom iPlex call rate was 90.2–96.9%, and the discordance rate was 0% among 4.2% DNA samples genotyped in duplicate.

1.4 BioServe Biotechnologies, Ltd. – Women's Health Initiative

The Women's Health Initiative samples (n=2,406 non-Hispanic women of European descent) were genotyped for all replication SNPs using the Sequenom iPLEX platform, which is based on multiplexed PCR followed by a mass extend reaction that produces an allele-specific extension product. After resin clean-up the extension products are spotted onto chips, and a laser

hits the products in a MALDI-TOF mass spectrometer which measures the mass of the extension products. Automated allele calling is carried out in real-time using Sequenom's Typer 3.4 software to convert the mass of the extension product to an allele call. The thresholds were all automated. Quality control was achieved by typing internal positive control samples of known genotypes with no template controls and by QC of replicate samples. 90 samples ~3% of the total were repeated. In group 1, for 14 SNPs there was 0% discordance and in group 2 for 8 SNPs there was 1% discordance. Genotyping plates were reviewed for results from positive- and negative-DNA control wells that are organized in specific patterns to assist in the QC process and to ensure correct plate orientations during processing and data review. In initial assay development, DNAs from 20 individuals from Coriell's Polymorphism Discovery Resource were used. SNP assays with genotype call rates of < 80% are excluded or redesigned. In SNPs with a high minor allele frequency the distributions of homozygote major, hetero and homozygote minor alleles were examined.

2. Supplemental Note:

2.1. Sex-specific analyses

Given the known sexual dimorphism of lean body mass, and evidence from variance decomposition studies that this might reflect sex-specific genetic effects,[4] we performed sex-specific meta-analyses of all successfully replicated SNPs to examine potential differences in association results between men and women. Furthermore, since two different methods (DXA and BIA) were used to measure lean mass, for successfully replicated associations, we also conducted meta-analysis in DXA and

BIA samples separately to assess whether similar results were obtained. Formal tests of heterogeneity by sex and by the technique used to measure lean mass were performed in the METAL package. **Supplemental Supplementary Table 2** provides results for men and women separately. Formal tests for heterogeneity by sex were performed using I^2 statistics implemented in the METAL package. In total, the discovery cohorts included up to 22,705 women and up to 15,587 men. We performed sex-specific meta-analyses for the eight successfully replicated associations. None of the sex-specific associations reached genome wide significance in either men or women, and the p-values for a formal interaction test between SNPs and sex did not reach statistical significance after multiple testing correction (the Bonferroni-corrected p-value threshold of < 0.00625 for 8 tests) (**Supplementary Supplementary Table 2**).

2.2. Technique-specific analyses

Two body composition techniques were employed by various participating studies. The first technique was dual energy x-ray absorptiometry (DXA). DXA uses an x-ray tube that produces two energy peaks. The ratio of soft-tissue attenuation (R_{ST}) at the two energies is measured. The attenuation of pure fat (R_F) and of bone-free lean tissue (R_L) are known from both theoretical calculations and human experiments. Given a subject's R_{ST} and the known R_S for fat and lean, one can solve two equations (one at each x-ray energy) with two unknowns to calculate the proportion of fat and lean tissue in each pixel.

Bioimpedance analysis (BIA) is another approach to estimating body composition. It relies on the geometrical relationship between:

1. Impedance of the body (composed of the resistance of the fat free mass and the reactance produced by the capacitance of cellular membranes, tissue interfaces and nonionic tissues
2. Height of the subject
3. Volume of fat free mass

Further refinements of BIA have produced equations using reactance and resistance to predict appendicular skeletal muscle mass.[5] Cohorts with access to the reactance and resistance data for their BIA measures calculated the appendicular lean mass using the equation developed by Kyle.[6] Genome wide association analyses with whole body and appendicular lean mass from cohorts with BIA were meta-analyzed separately from cohorts with whole body lean mass and appendicular lean mass derived from DXA. Formal tests for heterogeneity by the technique used to measure whole body and appendicular lean mass were performed using I^2 statistics implemented in the METAL package.

Cohorts participating in this project had measures of body composition obtained using either DXA (up to 21,074 subjects) or BIA (up to 17,218 subjects). Based on previous work showing a high correlation between the two phenotypes,[7, 6] the primary analysis combined all discovery cohorts regardless of technique used to measure lean mass. Nevertheless, we also stratified the discovery meta-analysis by technique of lean mass measurement, and performed secondary technique-specific meta-analyses for the eight successfully replicated associations. In general there was no evidence of heterogeneity, with all effect sizes being in the same direction and of similar magnitude for DXA vs. BIA (**Supplementary Supplementary Table 3**).

2.3. Replication and meta-analyses with all replication samples

See Methods section of manuscript.

2.4 Candidate genes reported previously

We looked at all associations between 1,440 SNPs in the four candidate genes previously reported to be associated with lean mass including *GREM1, CNTF, GLYAT, TRH,* and *PRDM16.* After correcting for multiple testing using a false discovery rate of 0.05, none of the SNPs in any of the genes were significantly associated with either whole body or appendicular lean mass.

2.5 Expression Analyses

Muscle tissues:

GTEx[8] (Genotype Tissue Expression program): A total of 138 postmortem donors with available muscle tissues were RNA-sequenced. Expression levels from RNA-seq data (standard GTEx protocol with filtered on ≥ 10 individuals having > 0.1 Reads Per Kilobase per Million was log transformed and quantile-normalized across all samples. Genome-wide genotyping was performed by Illumina Human Omni 5 DNA chip (Illumina, Inc., San Diego, CA). Un-genotyped SNPs were imputed based on the 1000 Genome Phase-1 reference panel. SNPs with MAF < 5% and INFO (an index of imputation quality from the IMPUTE package[9]) < 0.4 were excluded from eQTL analyses. We studied *cis*-eQTLs of the 5 replicated GWAS SNPs or their proxies (SNPs in LD with $r^2 \geq 0.7$) with selected transcripts within 500 kb of the SNP position. A multiple linear regression model

(adjusted for gender, 3 genotyping PCs and 15 PEER factors[8]) with additive genetic effects was used to determine association between expression levels and genotypes. The analyses were done using the MatrixEQTL[10] R package.

STRRIDE **(**Studies of a Targeted Risk Reduction Intervention through Defined Exercise). The STRRIDE includes two clinical trials investigating the effects of exercise training on cardio-metabolic risk factors in adults with metabolic syndrome (NCT00200993 and NCT00275145; PI: WE Kraus). Details about each study are published elsewhere.[11,12] Skeletal muscle biopsies were done on each subject at baseline and following 9 months of exercise training. Only baseline samples were used to estimate eQTLs for the current study. Global gene expression (Affymetrix U133A plus 2.0 arrays; Affymetrix, Santa Clara, CA) was completed on 60 Caucasian subjects from the total study (representing 5 men and 5 women from each of treatment 6 groups) according to manufacturer instructions. Three samples did not pass quality control measures for either cRNA input to microarray or microarray result quality using standard limits. Array data were quantified using the PLIER algorithm in Expression Console (Affymetrix). Genomic DNA was isolated from blood or skeletal muscle using DNeasy kits (Qiagen, Valencia, CA) and amplified using REPLI-g mini kits (Qiagen). Genotypes were generated from HumanOmni5-Quad BeadChips, using a Highscan system and GenomeStudio's Genotyping module (V1.9.4) (Illumina, Inc., San Diego, CA) with standard settings. A total of 57 samples (28 men; 29 women) were available for eQTL analyses. We studied the *cis*-eQTL of the 5 replicated GWAS SNPs with selected transcripts within 500 kb of the SNP position. A multiple linear regression model (adjusted for age, sex, study centers, BMI) with additive genetic effects was used to determine association between expression levels and genotypes.

FUSION (Finland United States Investigation of NIDDM): The FUSION RNA Seq eQTL sample consisted of 267 thigh skeletal muscle biopsies from fasting Finnish individuals with normal glucose tolerance (n=97), impaired glucose tolerance (n=69), impaired fasting glucose (n=35) and type 2 diabetes (n=66). We performed tissue sampling, RNA preparation, quality control and eQTL analysis as previously described (Scott LJ *et al.* The genetic regulatory signature of type 2 diabetes in human skeletal muscle.[13] Briefly we Poly A selected RNA and performed sequencing to a mean depth of 91.3M strand-specific paired-end reads. Following quality control, we retained genes with expression levels ≥5 counts in ≥25% of samples, and calculated FPKM based on the nuclear reads. Genome-wide genotyping was performed with the HumanOmni2.5-4v1_H BeadChip array (Illumina, San Diego, CA, USA). We performed SNP imputation using a two-step strategy.[14] For the reference panel we used the haplotypes from 2,737 European individuals sequenced in the Genetics of Type 2 Diabetes (GoT2D) project and used Minimac[15] to perform the imputation. SNPs with fewer than 5 allele counts and r^2hat (an index of imputation quality from the Minimac package) < 0.3 were excluded from eQTL analyses. For cis-eQTL analysis we 1) performed factor analysis via PEER[16] on the inverse normalized FPKM including age, sex, oral glucose tolerance test status, the top 2 genotype-based principle components and batch as covariates in the model and 2) inverse normalized the residuals. We included 60 Factors in the PEER analysis as that maximized the number of eQTLs. A linear regression model (implemented in the MatrixEQTL R package) with additive genetic effect was used to determine the association between the inverse normalized residual expression levels and the imputed allele count. For this analysis, we accessed results for 5 SNV in 8 genes, for a total of 8 SNP-transcript pairs.

To determine if the mean muscle mass GWAS variant, rs993685-FTO cis eQTL, was likely independent of other FTO cis-eQTLs, we performed iterative conditional analysis. We predicted Y_{ij} (as defined for cis-eQTL analysis), beginning with the GWAS SNP genotype in the model. We then performed step-wise forward selection of SNPs within 1Mb of the most upstream TSS (ie. we individually analyzed each SNP in the preceding model, retained the most significant SNP in the model, and repeated the procedure until no added SNP had a p-value < our stopping threshold). We used a stopping threshold of a p-value of 0.0019 (corresponding to the p-value threshold for gene-based cis-eQTLs with FDR<5% from our entire eQTL analysis as detailed in Scott et al. (Scott LJ *et al.* The genetic regulatory signature of type 2 diabetes in human skeletal muscle. *Nat. Comm.* In Press).

Pima Indian samples: Biopsies of *vastus lateralis* muscle were obtained from 225 healthy Pima Indians living in Arizona. Specimens were snap frozen and stored at -20 to -70°C. The rRNA was prepared and the cDNA synthesized for the Human Exon 1.0 ST Array microarray chips (Affymetrix, Santa Clara, CA, USA) using the GeneChip Whole Transcript Sense Target Labeling Assay kits (Affymetrix, Santa Clara, CA). Only transcript clusters identified as "core" (genes which have been identified with a high level of confidence) were analyzed, comprising a total of 17,881 genes. To account for varying intensity levels and other variability between different scans, we normalized the 225 chips using the Robust Multichip Average method with quantile normalization of the log2 intensities with prior GC correction[17] using the mean of all probes associated with the given gene. The level of each transcript was adjusted for batch effect and then normalized by sex, using an inverse Gaussian

transformation, prior to analysis. Of 5 SNPs of interest, 2 were monomorphic in the Pima Indian population. Genotypes of 2 SNPs were obtained using a custom-designed Axiom array (Affymetrix, Santa Clara, CA) designed to capture variants with minor allele frequency ≥0.05 observed in 296 Pima Indians with whole genome sequencing data. Genotypes of 1 SNP (rs4842924) were imputed based on data from the Axiom chip, using the whole-genome sequencing data of 296 Pima Indians as a reference in the program Minimac.[15] The associations between 3 SNPs and 5 transcripts (for a total of 5 SNP-transcript pairs) were tested using linear regression analyses based on additive genetic models, adjusting for effects of age and population admixture. Analyses were modeled using *proc mixe*d command to include the empirically estimated identity-by-decent as a random effect in the SAS program (Cary, NC).

Chest wall muscle biopsies from patients undergoing thoracic surgery for lung and cardiac diseases[18]: A total of 75 chest wall muscle biopsies were obtained from patients undergoing thoracic surgery for lung and cardiac diseases. Tissue collection, RNA and DNA isolation, expression profiling, and DNA genotyping have been described previously.[18] A gene expression profile with Agilent custom array (Agilent Santa Clara, CA) and genome-wide genotyping of Illumina SNP genotyping arrays were available on all 75 patients. All gene expression levels were adjusted for age, sex, race, and study center. We estimated cis-eQTL of the 5 replicated GWAS SNPs or their proxies (SNPs in LD with $r^2 \geq 0.7$) with selected transcripts within 500 kb of the SNP position. The Kruskal-Wallis test was used to determine the associations between adjusted expression levels and genotypes.

Other Tissues:

Subcutaneous adipose, omental adipose and liver tissues: A gene expression profile with 34,266 known genes (Agilent custom array) and genome-wide genotyping of 782,476 SNPs (Affymetrix 500K and Illumina 650Y SNP genotyping arrays) were available on 610 human subcutaneous adipose samples,[19] 742 omental adipose samples[19] and 957 liver samples.[1] Tissue samples were either postmortem or surgical resections from organ donors. Tissue collection, RNA and DNA isolation, expression profiling, and DNA genotyping have been described previously.[1] All gene expression levels were adjusted for age, sex, race, and center. We studied the cis-eQTL of the 5 replicated GWAS SNPs or its proxy (SNPs in LD with $r^2 \geq 0.7$) with selected transcripts within 500 kb of the SNP position. The Kruskal-Wallis test was used to determine association between adjusted expression levels and genotypes.

Lymphocytes: Expression experiments in two different samples were performed. A gene expression profile with 20,599 genes (Affymetrix U133 Plus 2.0) and genome-wide genotyping of 408,273 SNPs (Illumina HumanHap300 Genotyping Beadchip) were available on 400 children from families recruited through a proband with asthma. The detailed study design was described elsewhere.[20] We also profiled expression levels using the Illumina Human 6 BeadChips on additional 550 children from the UK (recruited from families with atopic dermatitis probands). These individuals were genotyped using Illumina HumanHap300 Genotyping Beadchip. Inverse normal transformation was used to normalize the skewed distribution in both samples. MACH

package[21] was used to impute un-genotyped SNPs based on Phase II HapMap CEU panel. Association analysis was applied with FASTASSOC option implemented in MERLIN package[22]. Only cis-effects within 500 Kb of the transcript were tested.

<u>Primary Osteoblasts</u>: A gene expression profile with 18,144 known genes (Illumina Human Ref8v2 BeadChips) and genome-wide genotyping of 561,303 SNPs (Illumina 550k Duo chips) were available in 95 human Caucasian primary osteoblast samples. Primary osteoblasts were derived from trabecular bone of proximal femora obtained from donors undergoing total hip replacement. Tissue collection, RNA and DNA isolation, expression profiling, and DNA genotyping have been described in detail.[23] All gene expression levels were adjusted for sex and year of birth. We studied the *cis*-eQTL of the 5 replicated GWAS SNPs or their proxies (SNPs in LD with $r^2 \geq 0.7$) with selected transcripts within 500 kb of the SNP position. The linear regression model implemented in PLINK[24] was used to determine association between adjusted expression levels and genotypes.

2.6 SNP Annotation and Enrichment analysis of human tissue-specific regulatory elements of GWAS loci

For coding variants, we predicted their function by PolyPhen-2.[25] For all variants, we annotated potential regulatory functions of our replicated GWAS SNPs and loci based on experimental epigenetic evidence including DNase hypersensitive sites, histone modifications, and transcription factor binding sites in human cell lines and tissues from the ENCODE Project and the Epigenetic Roadmap Project. We first examined GWAS loci for GWAS lead SNPs and SNPs in high LD ($r^2 \geq 0.8$) with GWAS lead SNPs. We then identified potential enhancers and promoters in the GWAS loci across 127 healthy human

tissues/normal cell lines available in the ENCODE Project and the Epigenetic Roadmap Project from the HaploReg4 web browser [26] using ChromHMM.[27] To evaluate if replicated GWAS loci were enriched with regulatory elements in skeletal muscle tissue, we performed a hypergeometric test to examine whether estimated tissue-specific promoters and enhancers in each GWAS locus were enriched in 8 relevant skeletal muscle tissues/cell lines vs non-skeletal muscle tissues (119 tissues/cell lines). The permutation (100,000 permutations) with minimum p-value approach was performed to correct for multiple testing. Permutation *p-values* < 0.05 were considered statistically significant. In addition, we also performed enrichment analyses in smooth muscle tissues/cells, fat tissue, brain tissues, blood cells and gastrointestinal tract tissues. The 8 skeletal muscle relevant tissues/cells were excluded when conducting enrichment analyses for other tissue types.

Tissues/cell types included in the enrichment analyses:

Skeletal muscle tissue: HSMM Skeletal Muscle Myoblasts Cells, HSMM cell derived Skeletal Muscle Myotubes Cells, Fetal Muscle Trunk, Fetal Muscle Leg, Psoas Muscle, Skeletal Muscle Male, Skeletal Muscle Female, and Muscle Satellite Cultured Cells.

Smooth muscle tissue: Colon Smooth Muscle, Duodenum Smooth Muscle, Rectal Smooth Muscle, and Stomach Smooth Muscle.

Fat tissue: Adipose Nuclei, Mesenchymal Stem Cell Derived Adipocyte Cultured Cells, Adipose Derived Mesenchymal Stem Cell Cultured Cells.

Brain tissue: Brain Angular Gyrus, Brain Anterior Caudate, Brain Cingulate Gyrus, Brain Germinal Matrix, Brain

Hippocampus Middle, Brain Inferior Temporal Lobe, Brain Dorsolateral Prefrontal Cortex, Brain Substantia Nigra, Fetal Brain Male, Fetal Brain Female, NH-A Astrocytes Primary Cells, Cortex derived primary cultured neurospheres, and Ganglion Eminence derived primary cultured neurospheres.

Blood tissue: Primary T cells from cord blood, Primary T cells from peripheral blood, Primary T helper memory cells from peripheral blood 2, Primary T helper naive cells from peripheral blood, Primary T helper memory cells from peripheral blood 1, Primary T regulatory cells from peripheral blood, Primary T cells effector/memory enriched from peripheral blood, Primary T CD8+ naive cells from peripheral blood, Primary T CD8+ memory cells from peripheral blood, Primary mononuclear cells from peripheral blood, Dnd41 TCell Leukemia Cell Line, GM12878 Lymphoblastoid Cells, K562 Leukemia Cells, Monocytes-CD14+ RO01746 Primary Cells, Primary monocytes from peripheral blood, Primary neutrophils from peripheral blood, Primary B cells from cord blood, Primary B cells from peripheral blood, Primary hematopoietic stem cells, Primary hematopoietic stem cells short term culture, Primary Natural Killer cells from peripheral blood, Primary hematopoietic stem cells G-CSF-mobilized Female, and Primary hematopoietic stem cells G-CSF-mobilized Male.

Gastrointestinal tract tissue: Colonic Mucosa, Duodenum Mucosa, Esophagus, Fetal Intestine Large, Fetal Intestine Small, Fetal Stomach, Gastric, Rectal Mucosa Donor 29, Rectal Mucosa Donor 31, Sigmoid Colon, Small Intestine, and Stomach Mucosa.

2.7 RNA-Seq analysis of gene expression in young versus old men, comparison with GWAS

Skeletal muscle biopsies from the *vastus lateralis* were taken from 19 healthy young (mean age 21 years, range 19-29 years, SD 2.7) and 18 healthy older (mean age 69 years, range 64-79 years, SD 4.6) male individuals using established procedures.[28,29] We used only males to maximize the likelihood of detecting differential gene expression using RNA sequencing (RNA-Seq). None of the subjects were performing regular physical exercise nor carrying out any form of exercise in the 48 hours preceding the biopsy, nor had they undergone an orthopedic procedure (i.e., joint arthroplasty) in the leg where the biopsy was taken. All subjects were studied between 0800h - 1200h in the fasted state with no caffeine or alcohol intake for 24 hours before the biopsy, and none of the subjects had significant medical disorders (diabetes, nerve or muscle disease, hypercholesterolemia requiring statins, or cardiovascular disease (other than hypertension requiring at most one medication)) or smoked. Samples were taken under ethical approval from the Hamilton Health Sciences Research Ethics board (IRB # 03-267, 05-376, 09-148) and all subjects provided informed, written consent. RNA was extracted from the tissue using standard protocols, and subjected to RNA-Seq using 50bp single reads on a HiSeq2000 using protocols recommended by the manufacturer. Data analysis was performed with the RNA-Seq workflow module of the *systemPiperR* package[30] available on Bioconductor.[31] Quality reports were generated with the *seeFastq* function. RNA-Seq reads were mapped with the splice junction aware short read alignment suite *Bowtie2/Tophat2*[32,33] against the *H.sapiens* genome sequence from Ensembl (Release 83). For the alignments, we used default parameters of *Tophat2* optimized for mammalian genomes. Raw expression values in

the form of gene-level read counts were generated with the *summarizeOverlaps* function.[34] We counted only reads overlapping exonic regions of genes, discarding reads mapping to ambiguous regions of exons from overlapping genes. Given the non-stranded nature of RNA-Seq libraries, the read counting was performed in a non-strand-specific manner. Of the 60,670 Ensembl IDs mapped, we then performed analysis of differentially expressed genes through use of the multtest package,[35] using the Benjamini-Hochberg method to correct for multiple testing. As our sample size was ~ 20 per group, we permuted the t-statistic 500,000 times to compare gene expression in the young group versus the old group, to minimize the likelihood that we would identify false positives.

We examined whether any SNPs in LD (r^2 >0.8) with our replicated SNP associations with whole body or appendicular LM were differentially expressed in young versus old muscles. Of 1,420 genes differentially expressed with age with a p-value of less than 0.05, none were in or near the five significant GWAS loci. Thus, there was no significant evidence of a relationship between the GWAS loci and the differentially expressed genes with age.

2.8. Discovery Cohorts

1. Age, Gene/Environment Susceptibility Reykjavik Study (AGES-Reykjavik): The AGES study was conducted between 2002-2006 on a random sample of surviving men and women from the Reykjavik Study[36]. Participants were born between 1907 and 1935 and lived in Reykjavik in 1967. Phenotypic data were collected using standardized protocols; the clinic visit included a blood draw, blood pressure measurement, electrocardiography, anthropometry and measures of different domains of physical and cognitive function. The AGES-Reykjavik Study GWAS was approved by the Icelandic National Bioethics Committee (VSN: 00-063), the Icelandic Data Protection Authority, and the MedStar Institutional Review Board. All subjects provided written informed consent.

2. Old Order Amish (OOA): The Amish Family Osteoporosis Study was designed to identify genetic determinants of osteoporosis in the Old Order Amish (OOA) population from Lancaster County, PA USA[37]. As part of this study DXA scans, including body composition, were obtained. The OOA community was founded in the late 18th century by a small group of immigrants (~400) from Switzerland and now numbers nearly 30,000 individuals. This is a founder population with virtually all community members linking to a single, 14-generation pedigree. The study was overseen by the local institutional review board and all participants provided written informed consent.

3. Cardiovascular Health Study (CHS): Cardiovascular Health Study (CHS): The Cardiovascular Health Study is a population-based, prospective cohort study of ambulatory older adults ages 65 years or older.[38] A cohort of 5,201 non-institutionalized men and women were randomly selected and enrolled from Medicare eligibility lists in 4 U.S. communities in 1989-90; an additional 687 African American participants were recruited and enrolled in 1992-93. They have been followed every six months since enrollment by either yearly in- person visits, though 1999, or phone interviews. In 2006 an addition home/clinic visit was carried out. Multiple physical and biological tests have been carried out and outcomes adjudicated. GWAS has been performed on 3,868 participants.

4. CoLaus: The design of the CoLaus study has been described previously.[39] Briefly, it is a population-based study conducted between 2003 and 2006 which recruited over 6,000 subjects aged 35-75 years in Lausanne, Switzerland. The following inclusion criteria were applied: a) voluntary participation in the examination, including blood sample, b) aged 35-75 years, and c) Caucasian origin defined as having both parents and grand-parents Caucasian (determined by birth place). The Institutional Review Board of the Centre Hospitalier Universitaire Vaudois (CHUV) in Lausanne approved the study protocol and signed informed consent was obtained from participants.

5. deCODE Study: Individuals that participated in various studies at the Landspitali University Hospital Bone Densitometry Clinic in Iceland were included in this study, as well as individuals referred to the clinic by medical doctors. In particular, all participants in the University Hospital Bone Mineral Study; a population based and randomly selected 1,630 men and women (aged 30-85 years) participating in a study on various aspects of bone health[40,41]. Additional individuals were part of the Study on genetics of bone mass with deCode Genetics.[42,43] Body composition and bone mineral density were measured by a Hologic QDR 4500 scanner. All participants gave informed consent and the study was approved by the Data Protection Commission of Iceland (DPC) and the National Bioethics Committee of Iceland.

6. The EPIC Study: The EPIC Obesity study includes 3,847 participants and is nested within the EPIC-Norfolk Study, a population-based cohort study of 25,663 European men and women aged 39-79 years recruited in Norfolk, UK between 1993 and 1997[44]. The study was designed as a case-cohort study of which the cases (n = 1,685) were randomly selected from the obese individuals within the EPIC-Norfolk Study and are defined as those with a BMI ≥30 kg/m2. The control-cohort consists of 2,566 individuals randomly selected from the total EPIC-Norfolk Study and thus by design, 381 individuals are part of the control-cohort as well as the case group. The current results are based on the genome-wide association analyses in the population-based cohort. Body composition was assessed using BIA (Tanita 531; Tanita Europe GmbH, Sindelfingen, Germany).

7. Erasmus Rucphen Family (ERF) Study: ERF is a cross-sectional family-based study including 3,000 living descendants of 22 couples baptized in the community church around 1850-1900. The participants are not selected on any disease or other outcome. Details about the genealogy of the population have been described elsewhere.[45,46] The study protocol was approved by the medical ethics board of the Erasmus MC Rotterdam, the Netherlands.

8. Fenland: The Fenland Study is an ongoing population-based cohort study (started in 2005) designed to investigate the association between genetic and lifestyle/environmental factors with the risk of obesity, insulin sensitivity, hyperglycaemia and related metabolic traits in men and women aged 30 to 55 years old.[47,48] Potential volunteers were recruited from General Practice sampling frames in the Fenland, Ely and Cambridge areas of the Cambridgeshire Primary Care Trust in the U.K. Exclusion criteria for the study were: prevalent diabetes, pregnant and lactating women, inability to participate including terminal illness, psychotic illness, or inability to walk unaided. Currently, the study comprises more than 3,000 participants of whom the first 1,500 volunteers with complete anthropometric data were genotyped and included in the current analyses. Body composition was assessed using DXA (Lunar Prodigy Advanced fan beam scanner; GE Healthcare, Bedford, UK).

9. Framingham Osteoporosis Study (FOS) / Framingham Heart Study (FHS). The FHS was initiated in 1948 to study determinants of cardiovascular disease and other major illnesses. The Original Cohort included 5,209 men and women, aged 28-62 years at enrollment who have undergone routine biennial examinations.[49,50] In 1971, Offspring of the Original Cohort participants and Offspring spouses including 5124 men and women, aged 5 to 70 years, were enrolled into the Framingham Offspring Study. Offspring participants have been examined approximately every 4 years [51,52]. In the 1990s, DNA was obtained for genetic studies from surviving Original Cohort and Offspring participants. The body composition measurements used in this analysis have been previously described.[7]

10. Gothenburg Osteoporosis and Obesity Determinants Study (GOOD): The GOOD study was initiated to determine both environmental and genetic factors involved in the regulation of bone and fat mass[53]. Male study subjects were randomly identified in the greater Gothenburg area in Sweden using national population registers, contacted by telephone, and invited to participate. To be enrolled in the GOOD study, subjects had to be between 18 and 20 years of age. There were no other exclusion criteria, and 49% of the study candidates agreed to participate (n = 1,068). The study was approved by the ethics committee at the University of Gothenburg. Written and oral informed consent was obtained from all study participants.

11. Health Aging and Body Composition Study (Health ABC): The Health ABC Study is a NIA-sponsored ongoing cohort study of the factors that contribute to incident disability and the decline in function of healthier older persons, with a particular emphasis on changes in body composition in old age. Health ABC enrolled well-functioning, community-dwelling African American (n=1,281) and Caucasian (n=1,794) men and women aged 70-79 years between April 1997 and June 1998. Participants were recruited from a random sample of Caucasian and all African American Medicare eligible residents in the Pittsburgh, PA, and Memphis, TN, metropolitan areas. The key components of Health ABC include a baseline exam, annual follow-up clinical exams, and phone contacts every 6 months to identify major health events and document functional status between clinic visits. GWAS data used in this study are available from 1663 Caucasian participants.

12. Indiana: Recruitment was focused on Indiana families having two or more healthy sisters who were required to be within 10 years of one another in age[54]. The sample included 1524 premenopausal European American women aged 20–45 years from 762 sibships. Exclusion criteria included a history of chronic disease, taking medications known to affect bone mass or metabolism, inability to have BMD measured because of obesity (weight >136 kg), and abnormal blood biochemistry tests. Women who had irregular menses or a history of pregnancy or lactation within 3 months before enrollment were excluded; however, women taking oral contraceptives were not excluded. BMD was measured by dual-energy x-ray

absorptiometry (DPXL and Prodigy; Lunar Corp., Madison, WI) at lumbar spine (vertebrae 2–4) and hip (femoral neck). The study was approved by the Institutional Review Board of Indiana University-Purdue University Indianapolis. Informed written consent was obtained from all subjects before their participation in the study.

13 & 14. Kora (KORA F3 and KORA F4): The KORA study is a series of independent population-based epidemiological surveys of participants living in the region of Augsburg, Southern Germany[55]. In the KORA S3 study 4,856 subjects (1994/95, response 75%), and in KORA S4 in total 4,261 subjects have been examined (1999/2001, response 67%). 3,006 subjects participated in a 10-year follow-up examination of S3 in 2004/05 (KORA F3). Informed consent has been given by all participants. The study has been approved by the local ethics committee. All study participants underwent a standardized protocol by certified medical staff including measurement of body composition. Genotyping was performed for 1,644 randomly selected individuals in KORA F3 and 1,814 individuals from KORA F4.

15 & 16 (two cohorts). The London Life Sciences Population (LOLIPOP): The LOLIPOP study is an ongoing population based cohort study of ~30,000 Indian Asian and European Caucasian men and women, aged 35-75 years, recruited from the lists of 58 General Practitioners in West London, United Kingdom between 2002 and 2008[56,57]. Europeans were recruited if all 4 grandparents were born in the UK, and Indian Asians if all 4 grandparents were born in the Indian Subcontinent (countries of India, Pakistan, Sri Lanka or Bangladesh). Data on medical history, family history, current prescribed medication, cardiovascular risk factors, and alcohol intake were obtained by a trained research nurse using an interviewer-administered questionnaire. Physical assessment included blood pressure, and anthropometric measurements (height, weight, waist and hip circumference). Country of birth of participants, parents, and grandparents were recorded together with language and religion for assignment of ethnic subgroups. Other biomedical information was recorded and venous blood was taken for genetic analysis. All participants gave written informed consent, and the study was approved by the Local Research Ethics Committee. In a subset study of the LOLIPOP cohort, genome-wide association scans of ~200K

SNPs were genotyped on a Perlegen custom array in 1,005 European men aged 35-65 years, ascertained on Adult Treatment Panel (ATP) III criteria for metabolic syndrome. In another study, 879 males and females enriched with coronary artery disease were genotyped on the Affymetrix 500K array.

17 & 18. Rotterdam Study (RSI, RSII): RSI and RSII are ongoing prospective population-based cohort studies of Caucasian subjects aged 45 years and over, living in the Ommoord district of Rotterdam, the Netherlands[58]. The study was designed to investigate the incidence and determinants of chronic disabling diseases in the elderly. Rationale and design have been described previously[59]. For RSI, all 10,275 inhabitants aged 55 years and over were invited for baseline examination between August 1990 and June 1993, of those, 7,983 participated. In 1999, 3,011 participants (out of 4,472 invitees) who had become 55 years of age or moved into the study district since the start of the study were added to the cohort (RSII). In 2006, a further extension of the cohort was initiated in which about 6,000 subjects aged 45–54 years, living in the Ommoord district, were invited, of which 3,932 participated (RSIII). Questionnaires including menopause related questions were filled out by study nurses during the home interview, while blood samples were taken of over 70 percent of the participants at the research centre. The Rotterdam Study was approved by the medical ethics committee of the Erasmus University Medical School, and written informed consent was obtained from each subject.

19 & 20 (two cohorts). Twins UK (TUK): The data examined in the present study are from the TwinsUK Adult Twin Registry, described in detail elsewhere[60]. This is an ongoing research project, in which all participants gave written informed consent before entering the study and the St. Thomas' Hospital research ethics committee approved the project. The volunteer sample was collected from the general population through national media campaigns in the UK and without first ascertaining the presence of any individual characteristics, diseases or traits. The present study is based on 6652 individuals, all female twins, collected since 1992. Each individual in the sample had one or more repeated DXA measurements of total lean (LBM) and fat (FBM) mass, 6074 individuals had as well measurements of left and right upper

and lower limbs. Measurements were carried out from 1992 to 2011. The average age of individuals used in the current analysis was 46.9 (SD=12.4) and varied between 18 and 79. The subjects were genotyped using a combination of Illumina and HumanHap610-Quad arrays (Human Hap300 and the Human Hap610Q). Genotyping was performed by the Wellcome Trust Sanger Institute using the Infinium assay (Illumina, San Diego, USA) and Centre for Inherited Diseases Research (USA). Genotyping was made in two stages. The first sample based on 317k GWAS data included 1685 individuals (41 MZ twin pairs, 519 DZ twin pairs and565 single individuals, belonging to 351 MZ and 214 DZ sibships with only one sibling with genotype & phenotype measurements available). The second sample based on 610k GWAS data included 1679 individuals (45 MZ twin pairs, 579 DZ twin pairs, and 431 single individuals, belonging 282 MZ and149 DZ sibships respectively). Twins' zygosity was determined by a standard questionnaire and by multiplex DNA fingerprinting with variable tandem repeats as well as concordance of genome-wide genotyping. Two samples in total included 3364 individuals. As the cohort is overwhelmingly of North European ancestry (98%), participants of other ethnicities were removed using standard principle component analyses.

<u>Replication Cohorts</u>

1. AGES. Age, Gene/Environment Susceptibility Reykjavik Study (AGES-Replication): The AGES study was conducted between 2002-2006 on a random sample of surviving men and women from the Reykjavik Study[36]. Participants were born between 1907 and 1935 and lived in Reykjavik in 1967. Phenotypic data were collected using standardized protocols; the clinic visit included a blood draw, blood pressure measurement, electrocardiography, anthropometry and measures of different domains of physical and cognitive function. The AGES-Reykjavik Study GWAS was approved by the Icelandic National Bioethics Committee, the Icelandic Data Protection Authority, and the MedStar Institutional Review Board. All subjects provided written informed consent.

2. Berlin Aging Study II (BASE-II): is a multidisciplinary study initiated in 2009 investigating factors related to human aging[61]. All subjects are recruited from the Berlin metropolitan area and undergo an extensive phenotypic assessment, including a 2-day internal medicine examination. GWAS genotyping was performed using the "Genome-Wide Human SNP Array 6.0" [Affymetrix, Inc]) on 1125 BASE-II participants. Phenotypic data was available for 624 of these individuals.

3. B-vitamins in the prevention of osteoporotic fractures (B-PROOF): The B-PROOF study is a randomized double-blind placebo-controlled trial to determine the efficacy of daily oral supplementation with vitamin B12 and folic acid in the prevention of fractures in elderly people. The intervention comprises a period of two years and will be completed in the first half of 2013. The study is carried out by a consortium from the Erasmus Medical Center (Rotterdam, the Netherlands), VU University Medical Center (Amsterdam, the Netherlands) and Wageningen University, (Wageningen, the Netherlands). Details of the study design have been described previously [62]. A total of 2,919 persons aged 65 years and older with serum homocysteine concentrations ≥12 μmol/L were included between October 2008 and March 2011. GWAS data and baseline whole body DXA data were available in 1,073 participants. The medical ethics committee of Wageningen University approved the study and all participants gave written informed consent.

4. Calcium Intake Fracture Outcome Study (CAIFOS): 1500 women were recruited in 1998 for an RCT of calcium tablets, a 5-year population based study of ambulant Western Australian women aged over 70 years[63]. The patients are still being followed up as on epidemiological study of ageing. A recruitment letter was sent to 24,800 potentially eligible women using the electoral roll, on which almost 100% of women of this age are registered. Potential participants were excluded if they had an illness likely to limit involvement in the study for 5 years or they were taking bone active agents such as bisphosphonates or estrogen. The treatment phase concluded in 2003 and the major findings published in Archives of Internal Medicine in 2006. Informed consent was obtained from each participant, including consent to access linked

administrative health data from WADLS. The Human Rights Committee of the University of Western Australia approved the study. At baseline and 5 years whole body Dual-energy X-ray Absorptiometry (DXA) was undertaken using a Hologic 4500A machine (Hologic, Boston, MA, USA) calibrated daily.

5. Danish Osteoporosis Study (DOPS): DOPS was initiated in 1991 when 2016 perimenopausal women (within 2 years of menopause) were enrolled in the study.[64] The women were recruited at 4 Danish centers based on mailings to names and addresses of women between the age of 40 and 58 years of age. The aim of the study was to investigate predictors of osteoporosis and the effect of hormone replacement therapy. None of the women who entered the study were treated with hormone replacement therapy or other bone acting drugs at the time of entry. All the data used for this study is from baseline, before treatment with HRT was initiated.

6. Family Heart Study (FamHS): The FamHS (https://dsgweb.wustl.edu/PROJECTS/MP1.html) was begun in 1992 with the ascertainment of 1,200 families, half randomly sampled, and half selected because of an excess of coronary heart disease (CHD) or risk factor abnormalities as compared with age- and sex-specific population rates[65]. The families, with approximately 6,000 individuals, were sampled on the basis of information on probands from four population-based parent studies: the Framingham Heart Study, the Utah Family Tree Study, and two Atherosclerosis Risk in Communities (ARIC) centers (Minneapolis, MN and Forsyth County, NC). A broad range of phenotypes were assessed at a clinic examination. Approximately eight years later, study participants belonging to the largest pedigrees were invited for a second clinical exam. A total of 2,756 subjects of European ancestry in 510 extended families were examined. All subjects gave informed consent for participation in the study and all procedures were conducted under institutionally approved protocols for human subjects' research. Measurements of the most important CHD risk factors in the atherosclerosis, lipid, glucose metabolism, blood pressure, anthropometry domains were obtained, along with medical history, medication use and several biochemical and hematologic markers. At this clinical examination, fat free mass (FFM) was assessed by bioelectrical impedance

analysis[7]. The FamHS used the Illumina genotyping platform (974 subjects were genotyped with HumanHap550v1.1 chip, 249 with Human610-Quadv1 and 1,472 with a Human1M-Duov3 chip). The framework map excluded SNPs with HWE p-value < 10^{-6}, with MAF< 1%, and those not present in the HapMap.[66,67] These data were used to impute ~ 2.5M SNPs in a two stages, based on HapMap phased haplotypes of 60 CEU samples, release 22, build 36 using MACH (version 1.0.16).

7. GenMets (Health 2000): The Genmets sample is a subset of 2,212 individuals of the Health2000 study (http://www.terveys2000.fi/doc/methodologyrep.pdf) collected as metabolic syndrome cases and their matched controls.[68] A total of 2,173 individuals have been genotyped on Illumina 610K arrays. 598,203 SNPs were successfully called with Illuminus software. Sample exclusions included sample call rate (<95%), relatedness, heterozygosity and gender discrepancy. The SNP exclusions were done for MAF < 1%, call rate < 95%, SNP clustering probability for each genotype < 95%, HWE $p < 1 \times 10^{-6}$.

8. Helsinki Birth Cohort (HBCS): The HBCS is composed of 8 760 individuals born between the years 1934-44 in one of the two main maternity hospitals in Helsinki, Finland. Between 2001 and 2003, a randomly selected sample of 928 males and 1,075 females participated in a clinical follow-up study with a focus on cardiovascular, metabolic and reproductive health, cognitive function and depressive symptoms. Detailed information on the selection of the HBCS participants and on the study design can be found elsewhere.[69-71] The research plan of the HBCS was approved by the Institutional Review Board of the National Public Health Institute and all participants have signed an informed consent. Body composition was measured at the clinical visit using bioelectrical impedance analysis (BIA) with the InBody 3.0 eight-polar tactile electrode system, Biospace Co., Ltd, Seoul, Republic of Korea equipment. DNA was extracted from blood samples and genotyping was performed with the modified Illumina 610k chip by the Wellcome Trust Sanger Institute, Cambridge, UK according to standard protocols (N=1,728). Genomic coverage was extended by imputation using the HapMap phase II CEU data as the reference sample and MACH software. Data on genotype and phenotype were available in 1,648 participants.

9 & 10 (African American& European Ancestry): Johnston County Study: The Johnston County Osteoarthritis Project (JoCo) is an ongoing prospective cohort study with multiple time points and all the samples were collected from Johnston County in North Carolina. JoCo is mainly focused in the research field of OA occurrence and progression. In order to investigate the ethnic difference in OA, the project recruited both African Americans and Caucasians. The baseline samples were recruited between 1990 and 1997 and additional new samples were collected between 2003 and 2004. Participants were genotyped using Illumina Infinium 1M-Duo bead arrays at Express Analysis (Durham, North Carolina). Lean mass was measured by DXA (Hologic, Inc). A total of 1,274 subjects with both lean mass measurements and genotype data were included in this association study. The analysis was conducted in Caucasian and African Americans separately. The sample size in the Caucasian specific analysis is 894 (36.5% men). The sample size of the African American specific analysis is 380 (38.4% men).

11 & 12 (BIA & DXA cohorts). Korean Genome Epidemiology Study (KoGES): KoGES is an ongoing prospective, community-based cohort study to investigate the frequency and determinants of chronic diseases in Korea.[72] For this study, two communities, one from a rural Ansung and the other from an urban Ansan community, were selected. The baseline examination was performed in 2001~2002, and biennial follow-up examinations were performed. The age range for eligibility was 40~69 years. Of the 7,192 eligible individuals in Ansung, 5,018 were surveyed (70% response rate) using a cluster sampling method. A total of 15,580 individuals were eligible in Ansan, and we successfully recruited 5,020 (32.4%) using a random sampling method of the local telephone directory. The study protocol was approved by the ethics committee of the Korean Health and Genome Study. Dual energy X-ray Absorptiometry scans (DXA, Lunar Prodigy) have been performed between 2009~2010. Bioelectrical impedance analyses (BIA, Inbody 3.0, Biospace, Korea) have been performed between 2001~2002. The current study is based on subjects with a total lean mass measurement with DXA (n=2,350) or BIA (n=5,694).

13 & 14. Kora F3 and Kora F4: Study information is the same as for KORA F3 and KORA F4 in the discovery cohort section[55]. Genotyping with the Illumina CardioMetabochip was performed for a non-overlapping sample of 1,482 (KORA F3) and 1,222 individuals (KORA F4).

15. LOLIP-REP-IA610: 7,015 samples were initially recruited as CHD cases and controls from the LOLIPOP cohort (see above).[73,74] All were of Indian Asian origin. The samples were genotyped on the Illumina Human610-Quad BeadChip.

16. LOLIP-REP-IA_I: 2,684 samples were selected from a random sample of 4,100 Indian Asian men from the LOLIPOP study.[75] The samples were enriched for insulin resistance and component phenotypes. Participants were selected if they had type-2 diabetes or were in the top 12.5% of the distributions for waist-hip ratio or diastolic blood pressure or HDL cholesterol, or the bottom 12.5% of these phenotypes and glucose. Genome-wide association scans were performed at deCODE genetics (Reykjavik, Iceland), using the Illumina HumanHap300 BeadChip.

17. LOLIP-REP-IA_P: 1006 Indian Asian men aged 35-65 years were ascertained on Adult Treatment Panel (ATP) III criteria for metabolic syndrome from the LOLIPOP cohort.[57] Genome-wide association scans of ~200K SNPs were genotyped on a Perlegen custom array.

18. METSIM: The METSIM Study includes 10,197 men, aged from 45 to 73 years, randomly selected from the population register of the Kuopio town, Eastern Finland, and examined in 2005-2010[76]. The aim of the study is to investigate genetic and non-genetic factors associated with the risk of type 2 diabetes, cardiovascular disease, and insulin resistance –related traits in a cross-sectional and longitudinal setting. Study protocol includes collection on data on cardiovascular risk factors (smoking, exercise, diet, history of chronic diseases including coronary heart disease, stroke, cardiac failure, medication, history of diabetes or early onset coronary heart disease in the family), questionnaire on the FINDISC Score, measurement of height, weight, waist, hip, blood pressure (3 times), and bioimpedance for the evaluation of fat percentage. Laboratory studies include an oral glucose tolerance test to evaluate glucose tolerance (samples for glucose, insulin, proinsulin and free

fatty acids at 0, 30, and 120 minutes), as well as fasting laboratory measurements (lipids, lipoproteins, apolipoproteins, adiponectin, bilirubin, ALT, bile acids, hs-CRP, IL1-RA, IL-1beta, HbA1c, proton NMR metabolomics data including lipids and lipoproteins, low molecular weight molecules (including e.g. amino acids), and fatty acids of different lengths.

19. MrOS Sweden: The MrOS study[77] is a multicenter, prospective study including 3,014 elderly men in Sweden, Hong Kong (± 2,000), and the United States (± 6,000). The MrOS Sweden cohort consist of three sub-cohorts from three different Swedish cities (n=1,005 in Malmö, n=1,010 in Göteborg, and n=999 in Uppsala). Study subjects (men aged 69–80 years) were randomly identified using national population registers, contacted and asked to participate. To be eligible for the study, the subjects had to be able to walk without assistance, provide self-reported data, and sign an informed consent; there were no other exclusion criteria. The study was approved by the ethics committees at the Universities of Gothenburg, Lund, and Uppsala. Informed consent was obtained from all study participants.

20. MrOS U.S.: The MrOS study enrolled 5,994 participants from March 2000 through April 2002. Recruitment occurred at six US clinical centers (Birmingham, AL; Minneapolis, MN; Palo Alto, CA; Pittsburgh, PA; Portland, OR; and San Diego, CA) and was accomplished primarily through mass mailings targeted to age-eligible men. Eligible participants were community-dwelling men who were at least 65 years of age, able to walk without assistance from another person, and had not had bilateral hip replacements (in order to obtain a hip BMD measure). Details of the MrOS study design and recruitment have been published elsewhere.[78,79] Written informed consent was obtained from all participants, and the study was approved by the Institutional Review Board at each study site. Body composition was measured using dual energy x-ray absorptiometry (DXA) (Hologic, Inc., MA). A central quality control lab, certification of DXA operators, and standardized procedures for scanning were used to ensure reproducibility of DXA measurements. At baseline, a Hologic whole body phantom was circulated and measured at the 6 clinical sites. The variability across clinics was within acceptable limits, and cross-calibration correction factors were not required. To adjust for inter-clinic differences, statistical

models include indicator variables for the individual scanners. Each clinic scanned a Hologic whole body phantom throughout the study to monitor longitudinal changes, and correction factors were applied to participant data as appropriate.

21. Osteoporosis Prospective Risk Assessment study (OPRA): The study participants are Caucasian women from the population based OPRA cohort.[80] Of 1,604 women invited between December 1995 and May 1999, 1,044 (65%) attended at baseline. All the women were 75 years of age at invitation and almost all were self-ambulatory. No exclusion criteria were applied. All participants answered a detailed questionnaire regarding their general health; BMD and body composition was assessed by DXA. The data reported in this analysis is based on women for whom genotype and body composition data was available, corresponding to 902 women. All participants gave written informed consent and the Lund University Ethics Committee approved the study. The study was performed according to the principles of the Helsinki declaration.

22. Orkney Complex Disease Study (ORCADES): ORCADES is a family-based, cross-sectional study in the Scottish archipelago of Orkney collected data between 2005 and 2011.[81] Data for 889 participants aged 18 to 100 years from a subgroup of ten islands, were used for this analysis.

23. PEAK 25: The population based PEAK-25 cohort consists of young women living in Malmö, Sweden, between 1999 and 2004.[82] The women were all 25 years of age at inclusion. Initially 2,394 women were invited and after excluding subjects who were pregnant at the time of the baseline investigation or during the previous 12 months, a total of 1,061 women participated in the study. All participants answered a detailed questionnaire regarding their general health; BMD and body composition was assessed by DXA. The data reported in this analysis is based on women for whom genotype and body composition data was available, corresponding to 1001 women. All participants gave written informed consent and the Lund University Ethics Committee approved the study. The study was performed according to the principles of the Helsinki declaration.

24. The Prospective Investigation of the Vasculature in Uppsala Seniors (PIVUS): PIVUS was initiated in 2001.[83] Eligible were all subjects aged 70 years living in the community of Uppsala, Sweden. The subjects were randomly chosen from the population register. Of the 2,025 subjects invited, 1,016 participated. (www.medsci.uu.se/pivus). The investigation contained extensive invasive and non-invasive investigations of the vasculature, medical and lifestyle questionnaire, dietary recordings, and serum and whole blood sample collection. Repeat data collection with examinations has been done at age 75 years and is presently ongoing at age 80 years. Dual energy X-ray Absorptiometry scans (DXA, Lunar Prodigy) have been performed on average two years after the baseline investigation and is also performed at the latest investigation. The present analysis is based on participants with DNA samples and a total lean mass measurement by DXA at 72 years of age (n=856).

25. Relationship between Insulin Sensitivity and Cardiovascular Disease (RISC): The primary objective of the RISC study is to establish whether insulin resistance predicts the development of atherosclerosis as measured by cIMT.[84] Other objectives are to determine whether insulin resistance predicts the deterioration of CVD risk markers, onset of diabetes, and obesity. The study has also been designed to determine the genetic and environmental contributions to insulin resistance. Understanding the relative importance of genetic and environmental factors and how they interact is critically important for the development of specific treatments and for the identification of high-risk individuals. Further, the study aims to develop a novel method to identify more easily insulin resistant subjects in clinical practice. In brief, participants were recruited from the local population at 19 centers in 14 countries in Europe, according to the following inclusion criteria: either sex, age between 30 and 60 years, and clinically healthy, stratified by sex and age according to 10-year age groups. Initial exclusion criteria were: treatment for obesity, hypertension, lipid disorders or diabetes, pregnancy, cardiovascular or chronic lung disease, weight change of 5 kg or more in last 6 months, cancer (in last 5 yr), and renal failure. Exclusion criteria after screening were: arterial blood pressure 140/90 mm Hg or higher; fasting plasma glucose 7.0 mmol/liter or greater; 2-h plasma glucose [on a 75-g oral glucose tolerance test (OGTT)] 11.0 mmol/liter or greater; total serum

cholesterol 7.8 mmol/liter or greater; serum triglycerides 4.6 mmol/liter or greater; and electrocardiogram abnormalities. Baseline examinations began in June 2002 and were completed in November 2004. The present analysis is based on the 1308 subjects (718 women and 590 men, mean age 44 yr, BMI 26 kg.m_2, range 18–44) who satisfied all criteria and whose clamp study passed the quality control.

26. Rotterdam III. Rotterdam Study (RS): See discovery.[58]

27. Rush Memory and Aging Project: The Rush Memory and Aging Project (MAP)[85] is a longitudinal, clinical-pathologic cohort study of common chronic conditions of aging with an emphasis on decline in cognitive and motor function and risk of Alzheimer's disease (AD). The study enrolls older adults without known dementia who agree to an annual assessment of risk factors, blood donation and a detailed evaluation. Further, as a condition of entry all participants sign an Anatomical Gift Act and agree to donation of brain, the entire spinal cord and selected nerve and muscles at the time of death. Study participants are primarily recruited from continuous care retirement communities throughout north eastern Illinois and all clinical evaluations are performed at home visits. Since October 1997, about 1,600 participants have completed their baseline evaluation. The follow-up rate of survivors exceeds 90%, and the autopsy rate exceeds 80%. Annual whole-body bioimpedance measures using a portable Body Comp Scale (American Weights & Measure, California) were added in 2005. The current study is based on 617 men and women for whom GWAS and body composition data was available.

28. SHIP 2: SHIP 2 is the twelve-year follow-up of the Study of Health in Pomerania, which was established in 1997 in the Northeast of Germany.[86] For baseline SHIP, 7,008 subjects aged 20 – 79 years were randomly selected in twelve age and sex strata from population registries. The net sample (without migrated or deceased persons) comprised 6,267 eligible subjects of which 4,308 subjects participated (response 68.8%). Data of 1,548 participants from SHIP 2 with available BIA and genotype data were used in the present analyses. Fat mass was assessed by a BIA using a Nutriguard M (Data Input, Darmstadt, Germany).

29. SHIP TREND: SHIP TREND is an on-going population based study in the Northeast of Germany established in 2008[86]. In the present analyses data from 958 participants with available BIA and genotype data were used. Fat mass was assessed by a BIA using a Nutriguard M (Data Input, Darmstadt, Germany).

30. The Study of Osteoporotic Fractures (SOF): SOF is a prospective multicenter study of risk factors for vertebral and non-vertebral fractures.[87,88] From 1986 to 1988, the cohort enrolled 9,704 community-dwelling women 65 years old or older recruited from population-based listings in four U.S. areas: Baltimore, Maryland; Minneapolis, Minnesota; Portland, Oregon; and the Monongahela Valley, Pennsylvania. Women enrolled in the study were 99% Caucasian, with African American women initially excluded from the study due to their low incidence of hip fractures. From January 1989 to December 1990, all participants were invited to undergo a second examination. Blood samples for DNA extraction were collected in 6,795 from women attending the second study visit. Lean mass was estimated by the bioelectrical impedance method using Valhalla 1990B Bio-Resistance Body Composition Analyzers (Valhalla Scientific, San Diego, California)[89,90]. Fat mass was calculated by subtracting lean mass from total weight; percent body fat was fat mass expressed as a percentage of total weight. Participants were instructed to maintain a normal fluid balance and to abstain from vigorous activity, alcohol, and caffeine for 12 hours before the clinic visit.[91]

31. Uppsala Longitudinal Study of Adult Men (ULSAM): ULSAM was initiated 40 years ago (http://www2.pubcare.uu.se/ULSAM/).[92] In short, all men born 1920-24 and living in Uppsala municipality (n=2841), Sweden, were invited in 1970-1973 to a health investigation in which 2322 (82%) men aged around 50 years participated. Surviving men have been re-investigated at 60, 71, 77, 82, and 88 years of age. The screening examination program has contained an extensive medical and lifestyle questionnaire and interview, dietary recordings, over-night fasting serum sampling with analyses of minerals, glucose metabolism, lipids, and vitamins, anthropometric measurements, and linkage to nation-wide disease registers. Dual energy X-ray Absorptiometry scans (DXA, Lunar Prodigy) have been performed at

the two latest investigations. DNA from whole blood samples was obtained at the third investigation (age 71 years). The current study is based on men with DNA samples and a total lean mass measurement with DXA at age 82 years (n=496).

32. CROATIA-VIS (VIS): The VIS study is a family-based, cross-sectional study in the villages of Komiza and Vis on the isolated island of Vis off the Dalmatian coast of Croatia, that included 1,056 examinees aged 18-93. Blood samples were collected in 2003 and 2004 along with many clinical and biochemical measures and lifestyle and health questionnaires.

33. Women's Health Initiative (WHI): Data are from the WHI sub-cohort for Biomarkers and Genetics factors of Sarcopenia. The WHI is a large, nationwide, longitudinal study investigating factors that contribute to the development of major health outcomes affecting postmenopausal women, including cancer, heart disease, and osteoporotic fractures.[93] The women were between the ages of 50-79 years at enrolment and from several racial and ethnic backgrounds. They were followed for up to nine years in the main study (1995-2003) and then followed for an additional five years in the extension study. The participants in this sub-cohort are part of the WHI observational group, with a mean age of 64.0 years (n=3,000). Lean mass was assessed using dual energy X-ray absorptiometry (DXA) (QDR 2000, 2000+, and 4500, Hologic Inc., Waltham, MA) at the three WHI bone density centers. DNA samples were analysed by BioServe Biotechnologies, Ltd. Genome wide analysis was done on all minority participants on the Affymetrix 6.0 platform.

Supplementary References

1. Schadt EE, Molony C, Chudin E, et al. Mapping the genetic architecture of gene expression in human liver. PLoS Biol 2008;6:e107.
2. Greenawalt DM, Dobrin R, Chudin E, et al. A survey of the genetics of stomach, liver, and adipose gene expression from a morbidly obese cohort. Genome Res 2011;21:1008-16.
3. Fan JB, Oliphant A, Shen R, et al. Highly parallel SNP genotyping. Cold Spring Harbor symposia on quantitative biology 2003;68:69-78.
4. Zillikens MC, Yazdanpanah M, Pardo LM, et al. Sex-specific genetic effects influence variation in body composition. Diabetologia 2008;51:2233-41.
5. Pietrobelli A, Morini P, Battistini N, Chiumello G, Nunez C, Heymsfield SB. Appendicular skeletal muscle mass: prediction from multiple frequency segmental bioimpedance analysis. Eur J Clin Nutr 1998;52:507-11.
6. Kyle UG, Genton L, Hans D, Pichard C. Validation of a bioelectrical impedance analysis equation to predict appendicular skeletal muscle mass (ASMM). Clin Nutr 2003;22:537-43.
7. Roubenoff R, Baumgartner RN, Harris TB, et al. Application of bioelectrical impedance analysis to elderly populations. J Gerontol A Biol Sci Med Sci 1997;52:M129-36.
8. Consortium GT. The Genotype-Tissue Expression (GTEx) project. Nat Genet 2013;45:580-5.
9. Howie B, Marchini J, Stephens M. Genotype imputation with thousands of genomes. G3 (Bethesda) 2011;1:457-70.

10. Shabalin AA. Matrix eQTL: ultra fast eQTL analysis via large matrix operations. Bioinformatics 2012;28:1353-8.

11. Kraus WE, Torgan CE, Duscha BD, et al. Studies of a targeted risk reduction intervention through defined exercise (STRRIDE). Med Sci Sports Exerc 2001;33:1774-84.

12. Slentz CA, Bateman LA, Willis LH, et al. Effects of aerobic vs. resistance training on visceral and liver fat stores, liver enzymes, and insulin resistance by HOMA in overweight adults from STRRIDE AT/RT. Am J Physiol Endocrinol Metab 2011;301:E1033-9.

13. Scott LJ, Erdos MR, Huyghe JR, et al. The genetic regulatory signature of type 2 diabetes in human skeletal muscle. Nat Commun 2016;7:11764.

14. Howie B, Fuchsberger C, Stephens M, Marchini J, Abecasis GR. Fast and accurate genotype imputation in genome-wide association studies through pre-phasing. Nat Genet 2012;44:955-9.

15. Fuchsberger C, Abecasis GR, Hinds DA. minimac2: faster genotype imputation. Bioinformatics 2015;31:782-4.

16. Stegle O, Parts L, Piipari M, Winn J, Durbin R. Using probabilistic estimation of expression residuals (PEER) to obtain increased power and interpretability of gene expression analyses. Nat Protoc 2012;7:500-7.

17. Mason CC, Hanson RL, Ossowski V, et al. Bimodal distribution of RNA expression levels in human skeletal muscle tissue. BMC genomics 2011;12:98.

18. Hagg S, Skogsberg J, Lundstrom J, et al. Multi-organ expression profiling uncovers a gene module in coronary artery disease involving transendothelial migration of leukocytes and LIM domain binding 2: the Stockholm Atherosclerosis Gene Expression (STAGE) study. PLoS Genet 2009;5:e1000754.

19. Musunuru K, Strong A, Frank-Kamenetsky M, et al. From noncoding variant to phenotype via SORT1 at the 1p13 cholesterol locus. Nature 2010;466:714-9.

20. Dixon AL, Liang L, Moffatt MF, et al. A genome-wide association study of global gene expression. Nat Genet 2007;39:1202-7.

21. Li Y, Abecasis G. Mach 1.0: rapid haplotype reconstruction and missing genotype inference. Am J Hum Genet 2006:2290.

22. Abecasis GR, Cherny SS, Cookson WO, Cardon LR. Merlin--rapid analysis of dense genetic maps using sparse gene flow trees. Nat Genet 2002;30:97-101.

23. Grundberg E, Kwan T, Ge B, et al. Population genomics in a disease targeted primary cell model. Genome Res 2009;19:1942-52.

24. Purcell S, Neale B, Todd-Brown K, et al. PLINK: a tool set for whole-genome association and population-based linkage analyses. Am J Hum Genet 2007;81:559-75.

25. Adzhubei IA, Schmidt S, Peshkin L, et al. A method and server for predicting damaging missense mutations. Nat Methods 2010;7:248-9.

26. Ward LD, Kellis M. HaploReg: a resource for exploring chromatin states, conservation, and regulatory motif alterations within sets of genetically linked variants. Nucleic Acids Res 2012;40:D930-4.

27. Ernst J, Kellis M. ChromHMM: automating chromatin-state discovery and characterization. Nat Methods 2012;9:215-6.

28. Tarnopolsky MA, Pearce E, Smith K, Lach B. Suction-modified Bergstrom muscle biopsy technique: experience with 13,500 procedures. Muscle Nerve 2011;43:717-25.

29. Zykovich A, Hubbard A, Flynn JM, et al. Genome-wide DNA methylation changes with age in disease-free human skeletal muscle. Aging Cell 2014;13:360-6.

30. Girke T. systemPipeR: NGS workflow and report generation environment. Bioconductor software package. 2014.

31. Huber W, Carey VJ, Gentleman R, et al. Orchestrating high-throughput genomic analysis with Bioconductor. Nat Methods 2015;12:115-21.

32. Kim D, Pertea G, Trapnell C, Pimentel H, Kelley R, Salzberg SL. TopHat2: accurate alignment of transcriptomes in the presence of insertions, deletions and gene fusions. Genome biology 2013;14:R36.

33. Langmead B, Salzberg SL. Fast gapped-read alignment with Bowtie 2. Nat Methods 2012;9:357-9.

34. Lawrence M, Huber W, Pages H, et al. Software for computing and annotating genomic ranges. PLoS computational biology 2013;9:e1003118.

35. Pollard KS, Dudoit S, van der Laan MJ. Multiple Testing Procedures: R multtest Package and Applications to Genomics. Bioinformatics and Computational Biology Solutions Using R and Bioconductor: Springer; 2005.

36. Harris TB, Launer LJ, Eiriksdottir G, et al. Age, Gene/Environment Susceptibility-Reykjavik Study: multidisciplinary applied phenomics. Am J Epidemiol 2007;165:1076-87.

37. Streeten EA, McBride DJ, Pollin TI, et al. Quantitative trait loci for bone mineral density identified by genome-wide linkage scan to chromosomes 7q and 21q in men from the Amish Family Osteoporosis Study. J Bone Miner Res 2006;21:1433-42.

38. Fried LP, Borhani NO, Enright P, et al. The Cardiovascular Health Study: design and rationale. Annals of epidemiology 1991;1:263-76.

39. Firmann M, Mayor V, Vidal PM, et al. The CoLaus study: a population-based study to investigate the epidemiology and genetic determinants of cardiovascular risk factors and metabolic syndrome. BMC cardiovascular disorders 2008;8:6.

40. Steingrimsdottir L, Gunnarsson O, Indridason OS, Franzson L, Sigurdsson G. Relationship between serum parathyroid hormone levels, vitamin D sufficiency, and calcium intake. JAMA 2005;294:2336-41.

41. Gudmundsdottir SL, Indridason OS, Franzson L, Sigurdsson G. Age-related decline in bone mass measured by dual-energy X-ray absorptiometry and quantitative ultrasound in a population-based sample of both sexes: identification of useful ultrasound thresholds for osteoporosis screening. J Clin Densitom 2005;8:80-6.

42. Sigurdsson G, Halldorsson BV, Styrkarsdottir U, Kristjansson K, Stefansson K. Impact of genetics on low bone mass in adults. J Bone Miner Res 2008;23:1584-90.

43. Styrkarsdottir U, Halldorsson BV, Gretarsdottir S, et al. New sequence variants associated with bone mineral density. Nat Genet 2009;41:15-7.

44. Day N, Oakes S, Luben R, et al. EPIC-Norfolk: study design and characteristics of the cohort. European Prospective Investigation of Cancer. British journal of cancer 1999;80 Suppl 1:95-103.

45. Aulchenko YS, Heutink P, Mackay I, et al. Linkage disequilibrium in young genetically isolated Dutch population. Eur J Hum Genet 2004;12:527-34.

46. Pardo LM, MacKay I, Oostra B, van Duijn CM, Aulchenko YS. The effect of genetic drift in a young genetically isolated population. Ann Hum Genet 2005;69:288-95.

47. Willer CJ, Speliotes EK, Loos RJ, et al. Six new loci associated with body mass index highlight a neuronal influence on body weight regulation. Nat Genet 2009;41:25-34.

48. Rolfe Ede L, Loos RJ, Druet C, et al. Association between birth weight and visceral fat in adults. Am J Clin Nutr 2010;92:347-52.

49. Dawber TR, Kannel WB, Lyell LP. An approach to longitudinal studies in a community: the Framingham Study. Ann N Y Acad Sci 1963;107:539-56.

50. Dawber TR, Meadors GF, Moore FE, Jr. Epidemiological approaches to heart disease: the Framingham Study. Am J Public Health Nations Health 1951;41:279-81.

51. Feinleib M, Kannel WB, Garrison RJ, McNamara PM, Castelli WP. The Framingham Offspring Study. Design and preliminary data. Prev Med 1975;4:518-25.

52. Kannel WB, Feinleib M, McNamara PM, Garrison RJ, Castelli WP. An investigation of coronary heart disease in families. The Framingham offspring study. Am J Epidemiol 1979;110:281-90.

53. Lorentzon M, Swanson C, Andersson N, Mellstrom D, Ohlsson C. Free testosterone is a positive, whereas free estradiol is a negative, predictor of cortical bone size in young Swedish men: the GOOD study. J Bone Miner Res 2005;20:1334-41.

54. Koller DL, Ichikawa S, Lai D, et al. Genome-wide association study of bone mineral density in premenopausal European-American women and replication in African-American women. J Clin Endocrinol Metab 2010;95:1802-9.

55. Wichmann HE, Gieger C, Illig T, Group MKS. KORA-gen--resource for population genetics, controls and a broad spectrum of disease phenotypes. Gesundheitswesen 2005;67 Suppl 1:S26-30.

56. Yuan X, Waterworth D, Perry JR, et al. Population-based genome-wide association studies reveal six loci influencing plasma levels of liver enzymes. Am J Hum Genet 2008;83:520-8.

57. Kooner JS, Chambers JC, Aguilar-Salinas CA, et al. Genome-wide scan identifies variation in MLXIPL associated with plasma triglycerides. Nat Genet 2008;40:149-51.

58. Hofman A, Brusselle GG, Darwish Murad S, et al. The Rotterdam Study: 2016 objectives and design update. European journal of epidemiology 2015;30:661-708.

59. Hofman A, Darwish Murad S, van Duijn CM, et al. The Rotterdam Study: 2014 objectives and design update. European journal of epidemiology 2013;28:889-926.

60. Moayyeri A, Hammond CJ, Hart DJ, Spector TD. The UK Adult Twin Registry (TwinsUK Resource). Twin research and human genetics : the official journal of the International Society for Twin Studies 2013;16:144-9.

61. Bertram L, Bockenhoff A, Demuth I, et al. Cohort Profile: The Berlin Aging Study II (BASE-II). Int J Epidemiol 2014;43:703-12.

62. van Wijngaarden JP, Dhonukshe-Rutten RA, van Schoor NM, et al. Rationale and design of the B-PROOF study, a randomized controlled trial on the effect of supplemental intake of vitamin B12 and folic acid on fracture incidence. BMC Geriatr 2011;11:80.

63. Prince RL, Devine A, Dhaliwal SS, Dick IM. Effects of calcium supplementation on clinical fracture and bone structure: results of a 5-year, double-blind, placebo-controlled trial in elderly women. Arch Intern Med 2006;166:869-75.

64. Mosekilde L, Hermann AP, Beck-Nielsen H, Charles P, Nielsen SP, Sorensen OH. The Danish Osteoporosis Prevention Study (DOPS): project design and inclusion of 2000 normal perimenopausal women. Maturitas 1999;31:207-19.

65. Higgins M, Province M, Heiss G, et al. NHLBI Family Heart Study: objectives and design. Am J Epidemiol 1996;143:1219-28.

66. Kilpelainen TO, Zillikens MC, Stancakova A, et al. Genetic variation near IRS1 associates with reduced adiposity and an impaired metabolic profile. Nat Genet 2011;43:753-60.

67. Kraja AT, Vaidya D, Pankow JS, et al. A bivariate genome-wide approach to metabolic syndrome: STAMPEED consortium. Diabetes 2011;60:1329-39.

68. Perttila J, Merikanto K, Naukkarinen J, et al. OSBPL10, a novel candidate gene for high triglyceride trait in dyslipidemic Finnish subjects, regulates cellular lipid metabolism. Journal of molecular medicine 2009;87:825-35.

69. Barker DJ, Osmond C, Forsen TJ, Kajantie E, Eriksson JG. Trajectories of growth among children who have coronary events as adults. N Engl J Med 2005;353:1802-9.

70. Eriksson JG, Osmond C, Kajantie E, Forsen TJ, Barker DJ. Patterns of growth among children who later develop type 2 diabetes or its risk factors. Diabetologia 2006;49:2853-8.

71. Rikkonen K, Pesonen AK, Heinonen K, et al. Infant growth and hostility in adult life. Psychosom Med 2008;70:306-13.

72. Cho YS, Go MJ, Kim YJ, et al. A large-scale genome-wide association study of Asian populations uncovers genetic factors influencing eight quantitative traits. Nat Genet 2009;41:527-34.

73. Chambers JC, Zhang W, Li Y, et al. Genome-wide association study identifies variants in TMPRSS6 associated with hemoglobin levels. Nat Genet 2009;41:1170-2.

74. Chambers JC, Zhang W, Zabaneh D, et al. Common genetic variation near melatonin receptor MTNR1B contributes to raised plasma glucose and increased risk of type 2 diabetes among Indian Asians and European Caucasians. Diabetes 2009;58:2703-8.

75. Chambers JC, Elliott P, Zabaneh D, et al. Common genetic variation near MC4R is associated with waist circumference and insulin resistance. Nat Genet 2008;40:716-8.

76. Stancakova A, Kuulasmaa T, Paananen J, et al. Association of 18 confirmed susceptibility loci for type 2 diabetes with indices of insulin release, proinsulin conversion, and insulin sensitivity in 5,327 nondiabetic Finnish men. Diabetes 2009;58:2129-36.

77. Mellstrom D, Johnell O, Ljunggren O, et al. Free testosterone is an independent predictor of BMD and prevalent fractures in elderly men: MrOS Sweden. J Bone Miner Res 2006;21:529-35.

78. Orwoll E, Blank JB, Barrett-Connor E, et al. Design and baseline characteristics of the osteoporotic fractures in men (MrOS) study--a large observational study of the determinants of fracture in older men. Contemporary clinical trials 2005;26:569-85.

79. Blank JB, Cawthon PM, Carrion-Petersen ML, et al. Overview of recruitment for the osteoporotic fractures in men study (MrOS). Contemporary clinical trials 2005;26:557-68.

80. Gerdhem P, Brandstrom H, Stiger F, et al. Association of the collagen type 1 (COL1A 1) Sp1 binding site polymorphism to femoral neck bone mineral density and wrist fracture in 1044 elderly Swedish women. Calcif Tissue Int 2004;74:264-9.

81. McQuillan R, Leutenegger AL, Abdel-Rahman R, et al. Runs of homozygosity in European populations. Am J Hum Genet 2008;83:359-72.

82. McGuigan FE, Larzenius E, Callreus M, Gerdhem P, Luthman H, Akesson K. Variation in the BMP2 gene: bone mineral density and ultrasound in young adult and elderly women. Calcif Tissue Int 2007;81:254-62.

83. Michaelsson K, Lind L, Frystyk J, et al. Serum adiponectin in elderly men does not correlate with fracture risk. J Clin Endocrinol Metab 2008;93:4041-7.

84. Hills SA, Balkau B, Coppack SW, et al. The EGIR-RISC STUDY (The European group for the study of insulin resistance: relationship between insulin sensitivity and cardiovascular disease risk): I. Methodology and objectives. Diabetologia 2004;47:566-70.

85. Bennett DA, Schneider JA, Buchman AS, Barnes LL, Boyle PA, Wilson RS. Overview and findings from the rush Memory and Aging Project. Current Alzheimer research 2012;9:646-63.

86. Volzke H, Alte D, Schmidt CO, et al. Cohort profile: the study of health in Pomerania. Int J Epidemiol 2011;40:294-307.

87. Ensrud KE, Lipschutz RC, Cauley JA, et al. Body size and hip fracture risk in older women: a prospective study. Study of Osteoporotic Fractures Research Group. The American journal of medicine 1997;103:274-80.

88. Cummings SR, Black DM, Nevitt MC, et al. Appendicular bone density and age predict hip fracture in women. The Study of Osteoporotic Fractures Research Group. JAMA 1990;263:665-8.

89. Bioelectrical impedence analysis in body composition measurement. NIH Technology Assessment Statement. 1994 Dec 12-14:1-35.

90. Lohman TG, ed. Advances in Body Composition Assessment. Champaign, IL: Human Kinetics Books; 1992.

91. Margolis KL, Ensrud KE, Schreiner PJ, Tabor HK. Body size and risk for clinical fractures in older women. Study of Osteoporotic Fractures Research Group. Ann Intern Med 2000;133:123-7.

92. Byberg L, Gedeborg R, Cars T, et al. Prediction of fracture risk in men: a cohort study. J Bone Miner Res 2012;27:797-807.

93. Jackson RD, LaCroix AZ, Cauley JA, McGowan J. The Women's Health Initiative calcium-vitamin D trial: overview and baseline characteristics of participants. Annals of epidemiology 2003;13:S98-106.
